# Recyclable and Degradable Ionic-Substituted Long-Chain Polyesters

**DOI:** 10.1021/acssuschemeng.3c03141

**Published:** 2023-08-09

**Authors:** Anne Saumer, Stefan Mecking

**Affiliations:** Department of Chemistry, University of Konstanz, Universitätsstraße 10, 78457 Konstanz, Germany

**Keywords:** biobased, degradable, ionic polymer, polyethylene-like

## Abstract

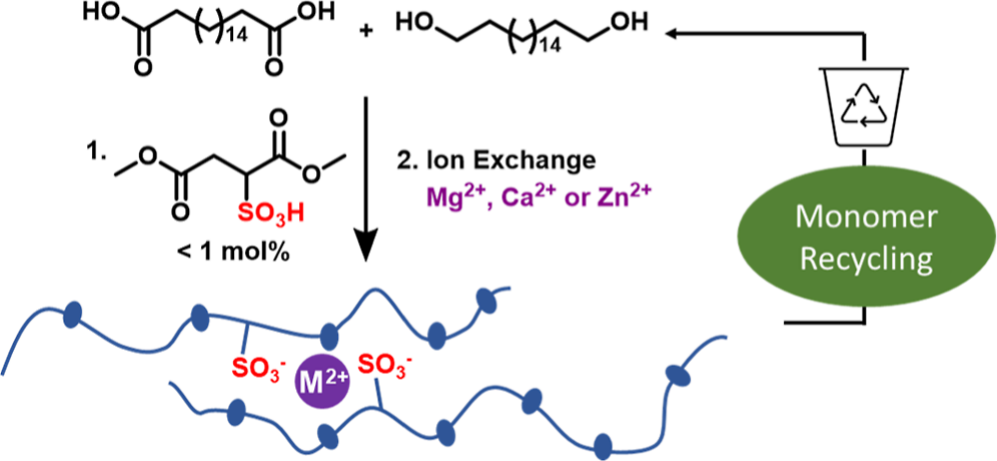

Ionic groups can endow apolar polymers like polyethylene with desirable traits like adhesion with polar compounds. While ethylene copolymers provide a wide range of tunability *via* the carboxylate content and neutralization with different cations, they lack degradability or suitability for chemical recycling due to their all-carbon backbones. Here, we report ion-containing long-chain polyesters with low amounts of ionic groups (*M*_n_ = 50–60 kg/mol, <0.5 mol % of ionic monomers) which can be synthesized from plant oils and exhibit HDPE-like character in their structural and mechanical properties. In the sulfonic acid as well as neutralized sulfonate-containing polyesters, the nature of the cation counterions (Mg^2+^, Ca^2+^, and Zn^2+^) significantly impacts the mechanical properties and melt rheology. Acid-containing polyesters exhibit a relatively high capability to absorb water and are susceptible to abiotic degradation. Enhanced surface wettability is reflected by facilitation of printing on films of these polymers. Depolymerization by methanolysis to afford the neat long-chain monomers demonstrates the suitability for chemical recycling. The surface properties of the neutralized sulfonate-containing polyesters are enhanced, showing a higher adsorption capability. Our findings allow for tuning the properties of recyclable polyethylene-like polymers and widen the scope of these promising materials.

## Abstract 2

We present the synthesis and materials’ properties of ion-containing, long-chain aliphatic polyesters which are degradable and fully recyclable to their biobased monomers.

## Introduction

The introduction of ionic groups into polymers is widely employed to achieve desirable properties.^1,2^ Considering polyethylene as the largest-scale synthetic polymer produced, while the major portion consists of hydrocarbon polymers like high-density polyethylene (HDPE), low-density polyethylene (LDPE), or linear low-density polyethylene (LLDPE), ionic-substituted polyethylenes are also employed in different applications. Such polymers are accessible by ethylene copolymerization or by various postpolymerization methods (*e.g.*, sulfonation), which alter the materials’ properties significantly as a result of Coulombic interactions between the ionic moieties.^3−5^ In case of acid-containing polyethylenes, hydrogen-bond networks emerge and introduce intra- and intermolecular interactions, complementing the van der Waals forces, which are typically decisive for the material properties.^6^ The neutralization of the acid groups with different cations leads to ion-containing polymers, which present an important class of materials with physical cross-links that enhance tensile strength or impact the melt viscosity.^5,7^ Also, an enhanced surface adhesion compared to PE with its low surface free energy can be beneficial.^5^ Ionic-substituted polyethylenes can be accessed by free radical copolymerization under high pressure of ethylene and acrylic comonomers, leading to random copolymers with poor control over the microstructure.^5^

The amount of ionic groups within the polymer can be varied over a broad range, higher concentrations being of interest with regard to proton or ion conductivity as polymer electrolytes, while low amounts of up to 10 mol % find applications in orthotics, prosthetics, as coatings of golf balls or as adhesives, to name a few.^8−11^ An industrially relevant example is Surlyn, a copolymer of ethylene and methacrylic acid developed by DuPont.^12^ Both the amount of polar comonomer and the percentage of acid group neutralization allow for a fine-tuning of the polymer properties.

There is an increasing need for sustainable materials using renewable feedstocks and for these materials to be (bio)degradable.^13,14^ Degradable polymers with ionic groups presented to date are restricted to short-chain polyesters, mainly based on polybutylene succinate (PBS) or polybutylene adipate terephthalate (PBAT).^15−17^ Sulfonated PBS polymers were intensely studied by Han *et al.*, and a significantly increased hydrolytic degradation rate was found with increasing ionic content.^15^ For sulfonated PBAT copolyesters, Wu *et al.* reported decreased thermal stability as well as impaired mechanical properties.^18^ However, the demonstrated higher hydrophilicity and water dispersibility are valuable properties to tune the biodegradation rate of PBAT. Other degradable ion-containing polymers are based on polycarbonates,^19−21^ polyurethanes,^22−24^ or polyphosphoesters.^25^ However, the limited data available on their mechanical properties suggest that applications are limited by a low toughness. Even though PBS- and PBAT-based ion-containing polymers are degradable, their mechanical properties limit their range of applications: while PBS is a brittle material, PBAT is rather soft and similar to LDPE.^26,27^ On the other hand, the large group of poly(ethylene-*co*-methacrylic acid) (PEMA) polymers, including Surlyn, does not exhibit degradability or chemical recyclability. The physical recyclability was shown in zinc-neutralized PEMA by Zhan *et al.*,^28^ yet any form of chemical recycling or indication of (bio)degradability is lacking to date.

We now present ion-containing polymers based on introducing low amounts of ionic groups with different cation counterions into polyester-18.18 (PE18.18), a long-chain aliphatic polyester with HDPE-like properties derived from fatty acid feedstocks. This allows for tuning the mechanical and rheological properties as well as abiotic degradability of these closed-loop recyclable materials. It was previously shown that PE18.18 with properties on par with HDPE is chemically recyclable in a closed-loop approach.^29^

## Results and Discussion

### Synthesis and Characterization

**Scheme 1:**
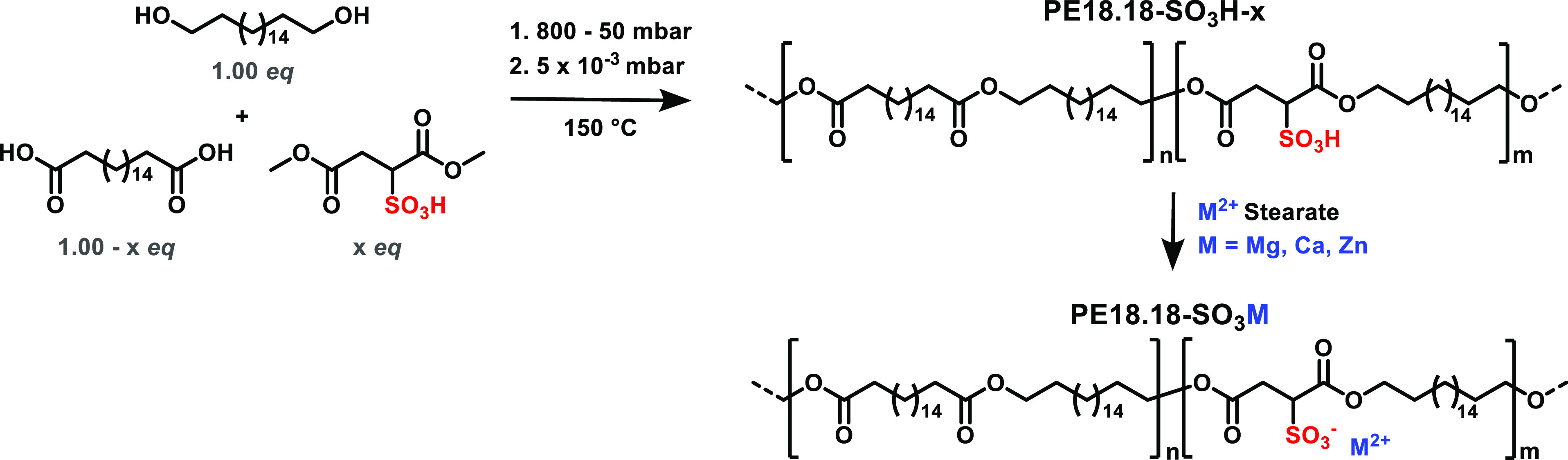
Synthesis of Sulfonate-Containing Polyesters Starting from the Respective Diol, Diacid, and Dimethyl Sulfosuccinic Acid (*x* = 0.005, 0.008 and 0.01); Subsequent Neutralization Reaction of PE18.18-SO_3_H-0.8 with Magnesium, Calcium, and Zinc Stearates

To introduce ionic sulfonate groups in long-chain all-aliphatic polyesters, HMSS (dimethyl sulfosuccinic acid) was covalently incorporated into a polyester-18.18 during the polycondensation synthesis from octadecane-1,18-dicarboxylic acid and octadecane-1,18-diol (cf. Scheme 1). As a reference polymer, polyester-12.12 (PE12.12) was studied. Since HMSS is a Brønsted acid and can function not only as a monomer but also as a catalyst for polycondensation reactions, no additional catalyst was required. For the synthesis of PE12.12-SO_3_H-1.0 (containing 1.0 mol % sulfosuccinic acid with respect to the total dicarboxylate) and PE18.18-SO_3_H-*x* (*x* = 0.5, 0.8, and 1.0 mol %), the mid- or long-chain diacid and diol, respectively, and HMSS were reacted at 150 °C under reduced pressure (see Figure 2a). After 4–6 h of reaction time, the reaction was terminated when the viscosity reached a limit where no more flow of the polymer melt was observed. The degree of polymerization and molecular weight was estimated from ^1^H NMR spectra and ^1^H TOCSY experiments were conducted to gain insight into proton correlations along the polymer chain. This revealed the successful in-chain incorporation of HMSS into the polyester chains. End-group analysis shows that carboxylic acid and alcohol end groups are found in the parent nonionic polyester, as well as methyl ester end groups in the form of mid- or long-chain dicarboxylate, originating from transesterification (for details of the analysis of the amount of sulfonic acid present in the polymers and TOCSY data, cf. the Supporting Information). Number average molecular weights range from *M*_n_ (NMR) 20 to 25 kg/mol (determined by end-group analysis in ^1^H NMR spectra). All determined values *M*_n_ are in a similar range and the molecular weights are sufficiently high to obtain satisfactory mechanical properties.

The presence of sulfonic acid groups in polyesters enables the introduction of other cations *via* neutralization reactions, as previously demonstrated by Lee *et al.* for PBS- and PBAT-containing phosphate groups.^30^ In this study, metal stearates were employed as neutralizing agents. On the one hand, excess metal stearate present in the final polymer is not expected to adversely affect polymer properties, given that it is used in commercially produced polymers as lubricants. For example, stearates facilitate the removal of the mold shape or enhance antistatic properties.^31^ On the other hand, the forming stearic acid can be removed in the workup process when the polymer is precipitated in ^*i*^PrOH from the xylene solution. As counterions, the bivalent cations magnesium, calcium, and zinc were employed. All compounds are commercially available in the form of metal stearates and are known to impact the material properties distinctly depending on their coordination strength to sulfonate groups.^32^ The prepolymer PE18.18-SO_3_H-0.8 containing 0.8 mol % of sulfosuccinic acid (PE12.12-SO_3_H: 1.0 mol %, cf. the Supporting Information for details of synthesis and characterization) as well as the metal stearates were separately dissolved in xylene to promote the neutralization reaction (Scheme 1).

**Figure 1:**
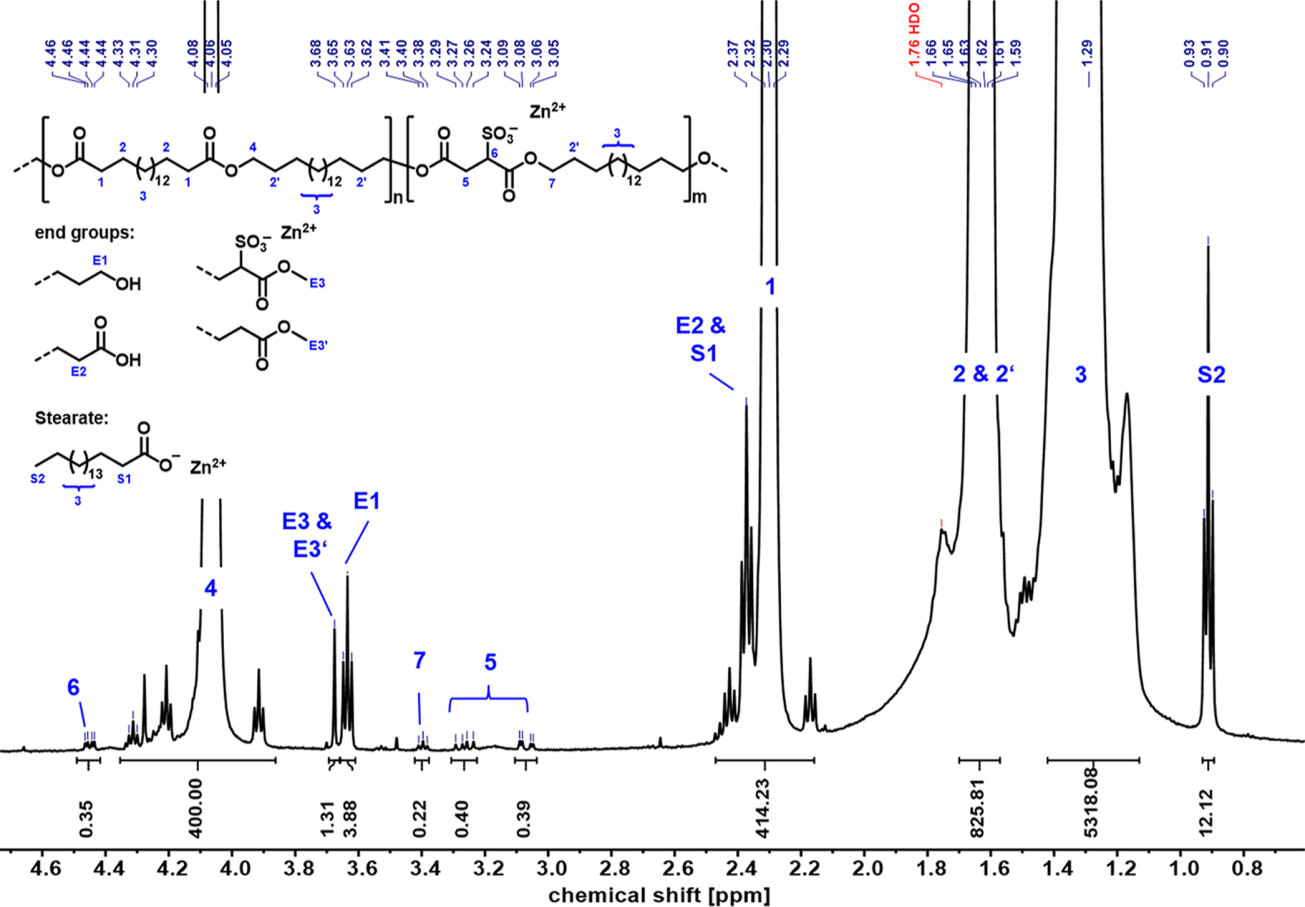
^1^H NMR (500 MHz, 323 K, C_2_D_2_Cl_4_) of PE18.18-SO_3_Zn. Note that protons H-5 are diastereotopic.

The polymer batch was divided into three equally sized fractions and the stearates were added each, upon which the viscosity of the polymer solutions was observed to significantly increase. Subsequently, the solutions were precipitated in cold ^*i*^PrOH, washed, and dried. The ^1^H NMR spectra remain unchanged regarding the prepolymer PE18.18-SO_3_H (or PE12.12-SO_3_H, respectively, see the Supporting Information). One additionally occurring signal at 0.8 ppm corresponds to the ω-methyl group of the stearate, showing that excess stearate is present in the final polymer, which impedes a calculation of the DP_n_ and M_n_ from ^1^H NMR spectra due to overlapping signals (Figure 1). All signals corresponding to the sulfosuccinate unit (H-5, H-6) can be identified, as well as the methylene unit upon esterification of a C_18_-diol and sulfosuccinate unit (H-7).

Additionally, polymer composites were synthesized without ionic groups within the polymer backbone but with metal stearate present in PE12.12 (PE12.12-M_stearate_, synthesis and properties can be found in the Supporting Information). These reference composites were compared to the ion-containing PE12.12-SO_3_M to identify any effects originating from the free stearate present. Molecular weights and dispersities of the neutralized polymers were determined by GPC analysis with linear calibration against polystyrene (Scheme 1a). The molecular weights of the sulfonate-containing polymers are comparable (*M*_n_ (GPC) 48–52 kg/mol for PE12.12-SO_3_M, ∼61 kg/mol for PE18.18-SO_3_M) and are similar to their non-functionalized reference polyesters (53 and 68 kg/mol, respectively). All dispersity indexes are in the range expected for polyesters obtained *via* polycondensation, *M*_w_/*M*_n_ = 2.0–2.2. Rheological data and mechanical testing suggest significant physical cross-linking of the chains, as discussed below. The aforementioned dispersity values give no indication of a corresponding aggregation in the chloroform solutions employed for GPC, which is likely due to the excess stearate coordinating with the polymer-bound metal cations (−SO_3_–M–O_2_C−), present in the solution in relatively high dilutions.

**Figure 2:**
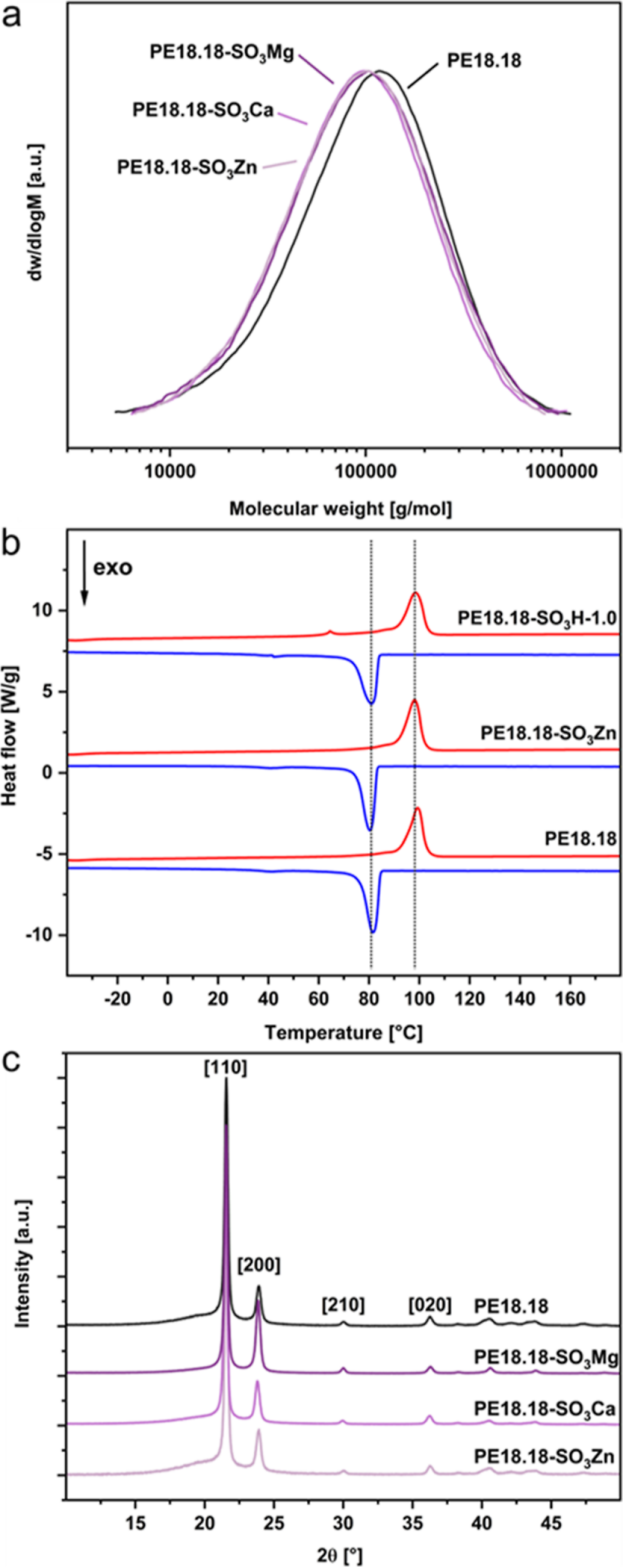
(a) GPC traces of PE18.18 and PE18.18-SO_3_M (chloroform, linear calibration against polystyrene). (b) DSC thermograms of PE18.18, PE18.18-SO_3_Zn, and PE18.18-SO_3_H-1.0. (c) WAXS profiles of PE18.18 and PE18.18-SO_3_M, peaks corresponding to the orthorhombic unit cell of polyethylene are labeled. Data were shifted vertically for clarity.

The amount of metal cations present in the final polymer was determined *via* elemental analysis and accounts for 0.10 wt % (PE18.18-SO_3_Mg), 0.13 wt % (PE18.18-SO_3_Ca), and 0.28 wt % (PE18.18-SO_3_Zn). These values are on par with the input amount of metal stearate in the reaction and underline that the excess metal is present in the final polymer, rather than removed in the workup process. The relative amounts are also well comparable to the reference composites (PE12.12-M_stearate_, for calculation and discussion, cf. the Supporting Information). A further insight into the ionic group distribution was gained by statistical calculations. Assuming a random distribution of the ionic groups along the polymer backbone, supported by the results from ^1^H NMR and TOCSY analysis, the amount of ionic groups per polymer chain was calculated. In the case of PE18.18-SO_3_H-0.8, it can be assumed that ∼60% of the polymer chains in PE18.18-SO_3_M are unfunctionalized, ∼25% are monofunctionalized, and ∼15% contain more than one sulfosuccinate unit. The significant impact on rheological and mechanical properties (as discussed below), despite the low degree of functionalization, makes these polymers of special interest.

The thermal properties of PE18.18, relevant for processing and applications, are not adversely affected upon the introduction of ionic groups, neither sulfonic acid groups nor Mg, Ca, or Zn sulfonates (Figure 2b). Also, the polyethylene-like character of PE18.18 regarding its crystal structure remains unaltered and reflexes corresponding to the orthorhombic unit cell of polyethylene are found in all ion-containing polymers (Figure 2c). The same holds true for all reference polymers PE12.12-SO_3_M and PE12.12-M_stearate_ (see the Supporting Information).

**Figure 3:**
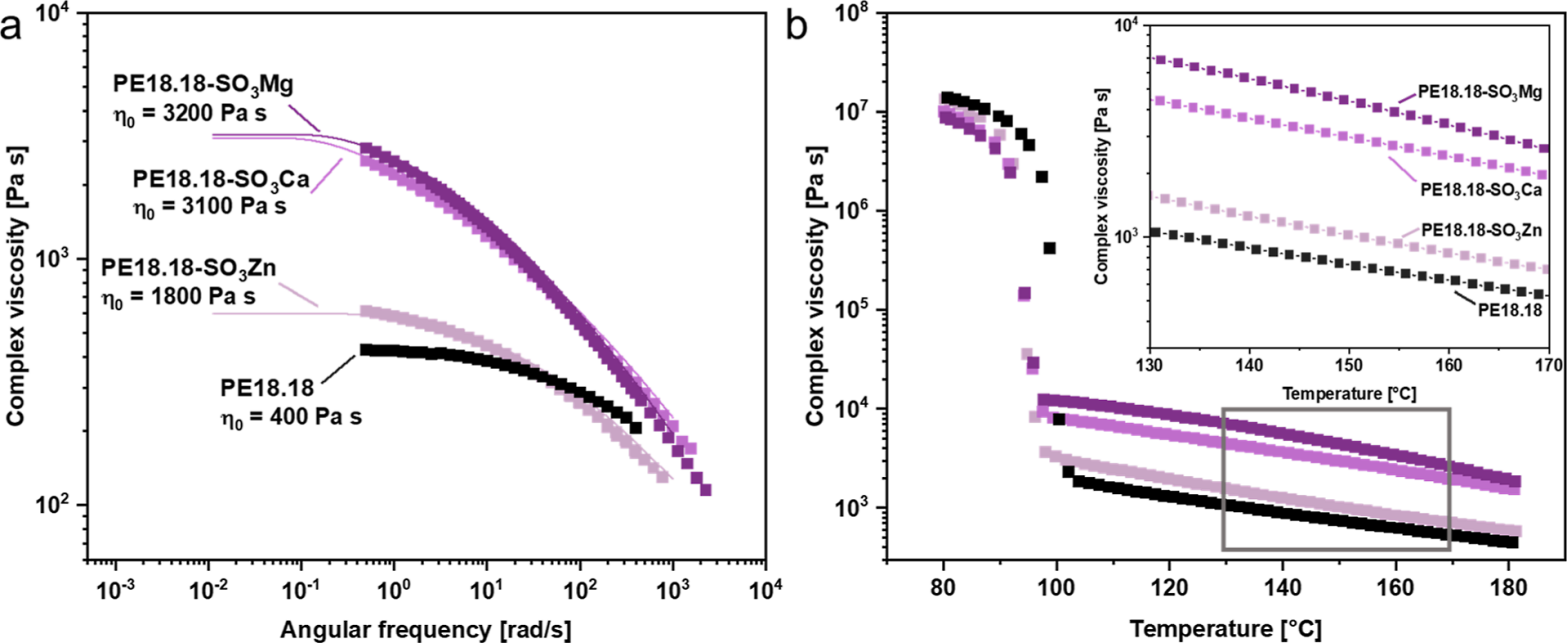
(a) Absolute complex viscosity as a function of the angular frequency of PE18.18 and PE18.18-SO_3_M at 180 °C. The solid lines represent the fit curve calculated with the multimode Maxwell model (10 modes). Zero-shear viscosities (η_0_) are listed. (b) Temperature sweep rheology results of PE18.18 and PE18.18-SO_3_M; inset shows section marked in gray in closer detail (strain 0.1%, angular frequency 6.28 rad/s, and heating rate 10 °C/min).

The zero-shear viscosity (η_0_) is significantly enhanced upon the introduction of ionic groups, where the Mg and Ca derivatives show a 7-fold increase and Zn a 4-fold increase compared to PE18.18 (Figure 3a). The increasing η_0_ in the order Zn–Ca–Mg, with Zn having a significantly lower value than Ca and Mg, shows the dependence of the cross-linking on the nature of the cation. This correlates with Mg being the smallest cation with the highest ionic associations, Ca being similar yet due to the larger ionic radius exhibiting lowered ionic associations, and Zn having a less ionic nature and more covalent character, therefore exhibiting lower Coulombic interaction.^33^ This was previously also observed in ionically cross-linked PBAT materials; however, the magnitude of the effect observed herein is much larger.^30^

Temperature sweep rheology measurements revealed that the strong difference in the melt viscosity prevails throughout the entire temperature range studied (80–180 °C, Figure 3b). No discontinuous changes are observed upon heating in the melt state, showing that the ionic aggregates are present at all temperatures below 180 °C.

### Processing and Materials’ Properties

**Figure 4:**
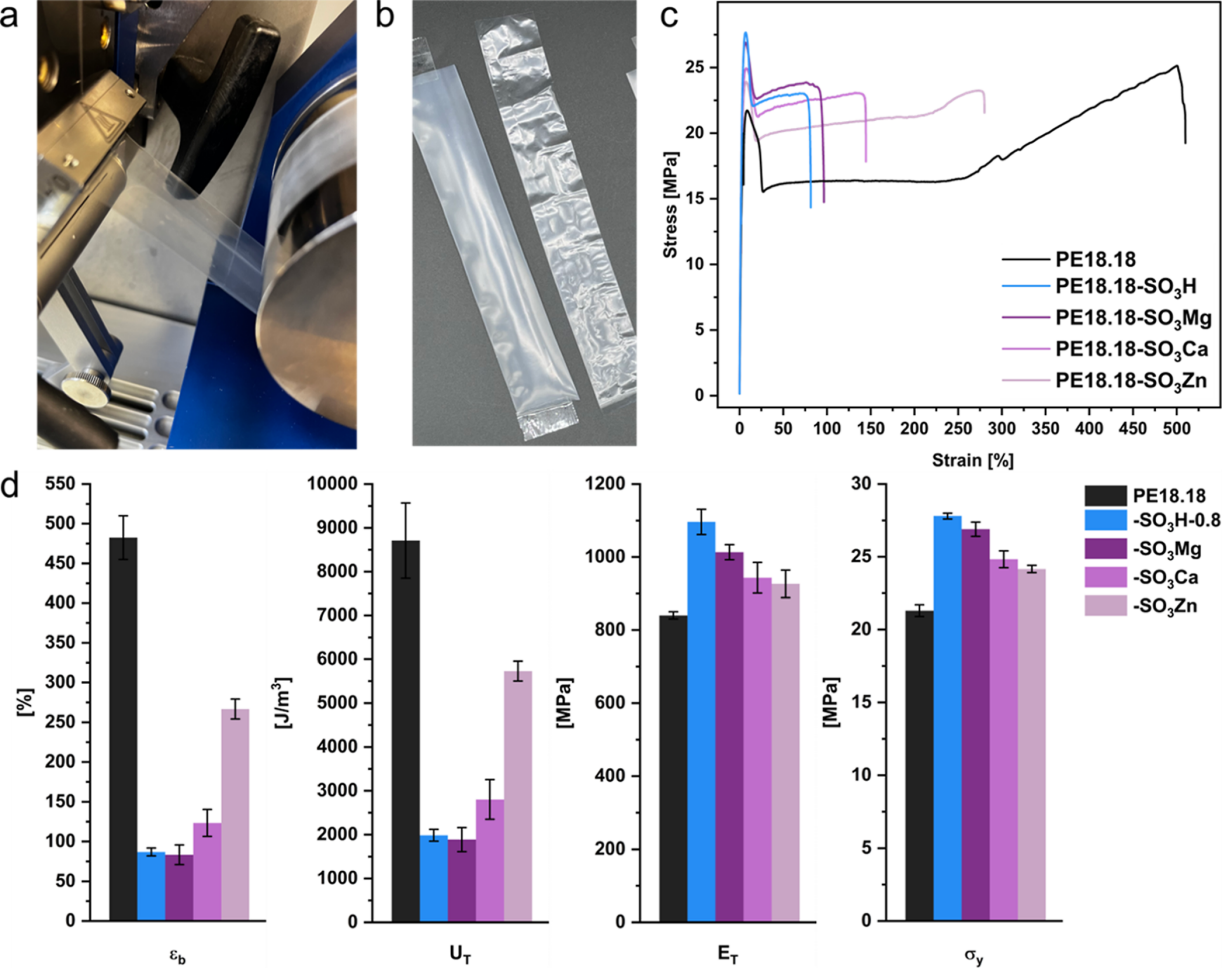
(a) Drawing of films (PE18.18-SO_3_Mg) from a micro-compounder with a micro-cast film line. (b) Exemplary films of different thicknesses (PE18.18-SO_3_Mg) drawn with the film line (left: 60 μm, right: 10 μm thick). (c) Exemplary stress–strain curves of PE18.18, PE18.18-SO_3_H, and PE18.18-SO_3_M. (d) Comparison of elongation at break (ε_tb_), tensile toughness (*U*_T_), Young’s modulus (*E*_T_), and stress at yield point (σ_y_) of PE18.18, PE18.18-SO_3_H-0.8, and PE18.18-SO_3_M. Error bars reflect standard deviation as determined from three test specimens.

Prior to processing the polymers at elevated temperatures, their thermal stability was investigated with thermogravimetric analysis (TGA), and it was found that all polymers, PE18.18, PE18.18-SO_3_H, and PE18.18-SO_3_M, as well as all PE12.12 derivatives, show comparable values for the temperature of 95% residual weight under air (>300 °C in all cases, see the Supporting Information for details). This supports that processing at 160–180 °C in the melt state is possible. All polymers containing sulfonic acid units could be melt-processed in a micro-compounder, extruded, and injection-molded to produce tensile testing specimens. Also, all neutralized polymers PE18.18-SO_3_M (and PE12.12-SO_3_M as well as the reference material PE12.12-M_stearate_) were successfully extruded and injection-molded into tensile testing specimens. Additionally, melt-extruded films were drawn with a micro-cast film line (see Figure 4a,b). Previously reported ion-containing polymers are typically not amenable to melt or solution processing, with the exception of compression molding.^10^ Consequently, the materials reported here are attractive in being compatible with industrial processing techniques.

The mechanical properties of the ion-containing polymers synthesized were investigated by tensile testing of injection-molded specimens (ISO 527-2, type 5A). An increasing sulfonic acid content in PE18.18-SO_3_H polymers leads to a significant decrease in ductility and tensile toughness and, hence, an increasing stiffness (for details, cf. the Supporting Information). For the neutralized materials, a strong dependence on the nature of the cation is observed (see Figure 4c,d). Magnesium- and calcium-containing polymers exhibit similar ductilities and tensile toughnesses as PE18.18-SO_3_H-0.8 (ε_tb_ ∼ 100%, *U*_T_ ∼ 2000 J/m^3^), which are significantly lower compared to PE18.18 (ε_tb_ ∼ 500%, *U*_T_ ∼ 9000 J/m^3^), and reflect the ionic associations also observed in the melt state. However, the zinc-containing polymer PE18.18-SO_3_Zn shows intermediate values (ε_tb_ ∼ 250%, *U*_T_ ∼ 5000 J/m^3^), in line with the lower ionic character of zinc cations’ interactions and, consequently, less strong ionic aggregations. Such an effect was previously discussed by Makowski *et al.* and was ascribed not only to a lower ionic character but also to the excess carboxylate present in the polymer (stearate in this case).^32^ The disulfonates −SO_3_–Zn–O_3_S– in sulfonated ethylene–propylene–diene–monomer terpolymer (EPDM) were stated to be broken up to form monosulfonates, −SO_3_–Zn–O_2_C–, with the excess stearate coordinating the zinc. Even though the carboxylate coordination is weaker than the sulfonate coordination, this is possibly more pronounced due to the lower ionic character of Zn^2+^ as opposed to Mg^2+^ or Ca^2+^.

The yield stress is unchanged for PE18.18-SO_3_M but is significantly enhanced in acid-containing polyesters. Such an effect was previously shown in ethylene-methacrylic acid copolymers and was attributed to the crystal plasticity as well as incomplete mechanical relaxation in the amorphous domains, which are influenced by the ionic aggregates.^34,35^

**Figure 5:**
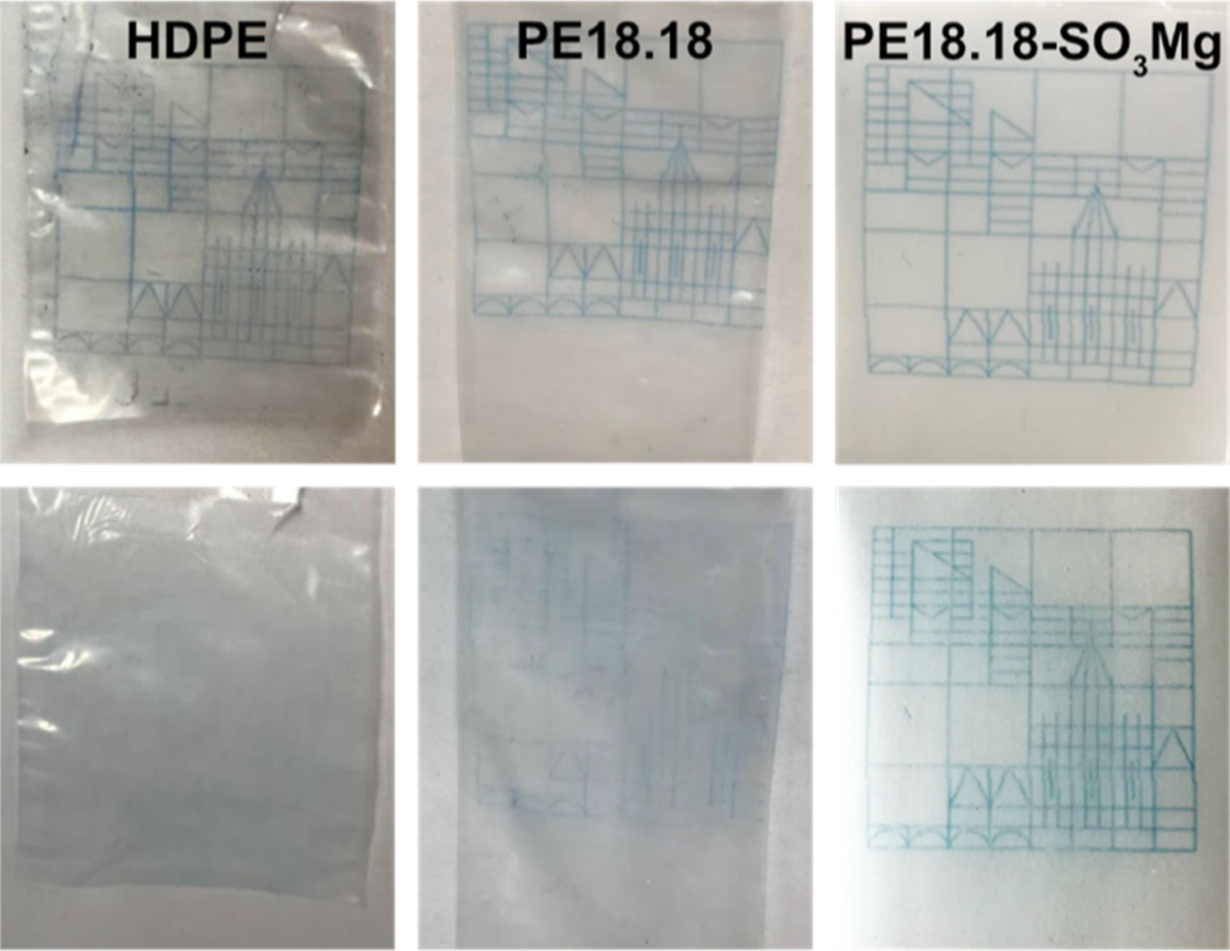
Photographs of imprinted films of HDPE, PE18.18, and PE18.18-SO_3_Mg, before (top row) and after (bottom row) rigorously wiping off the color with a microfiber cloth.

The surface properties of processed PE18.18-SO_3_H and PE18.18-SO_3_M were examined by means of the water contact angle (WCA), which shows a strong dependence on the amount of incorporated acid in case of PE18.18-SO_3_H and a reduced WCA by ∼ 10° compared to PE18.18 in case of PE18.18-SO_3_M, independent of the cation nature (data is given in the Supporting Information). A higher surface energy compared to PE18.18 can be concluded, and this was further probed by the adsorption of hydrophilic ink. Melt-extruded films of PE18.18 and PE18.18-SO_3_M produced with the micro-cast film line were employed as substrates for ink-jet printing. The adhesion of the print on the film surface was tested after a 24 h drying period by wiping it over with strong pressure using a microfiber cloth. Representative photographs of the imprinted films of HDPE, PE18.18, and PE18.18-SO_3_Mg before and after wiping are depicted in Figure 5 (images of PE18.18-SO_3_Ca and PE18.18-SO_3_Zn can be found in the Supporting Information). After rigorously wiping, a clear difference can be observed for the different materials. In the case of HDPE, no residue of the design remained, and on the PE18.18 film, it was significantly blurred. The slightly better adsorption capability of PE18.18 as opposed to HDPE is conclusive, since the ester groups PE18.18 enhance the surface free energy, leading to somewhat better adhesion. In case of PE18.18-SO_3_M, good adsorption of the color can be seen: the difference between the samples before and after wiping off the color is small, and improved adhesion compared to PE18.18 and HDPE is observed. A slight blurring is observed in this case as well but to a much lower extent than in PE18.18. The delicate lines of the logo can be identified after wiping on all ion-containing derivatives. To investigate whether the observed effect derives from the incorporated stearates or the polymers’ ionic groups (or a combination thereof), imprinted films of PE12.12-SO_3_M were compared to polyesters PE12.12-M_stearate_ without sulfonate groups but containing stearates. The results show that the improved adsorption is in close correlation with the covalently attached ionic groups (cf. the Supporting Information for detailed data and discussion). This feature of the polymers reported here is attractive for packaging and other applications like (textile) fibers that require printing or dying, respectively, on the material compared to HDPE, which requires a significant degree of surface modification prior to printing.^36^

### Chain Breakdown

**Figure 6:**
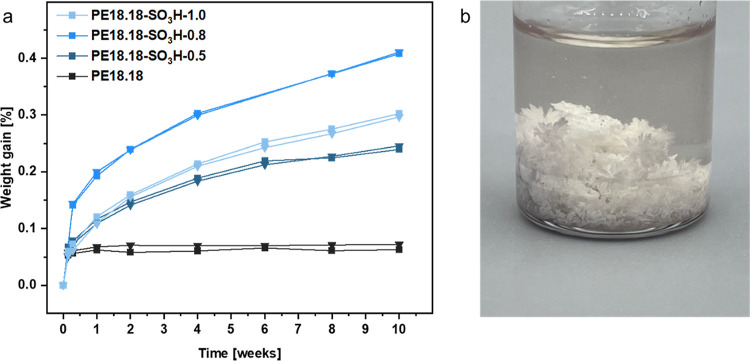
(a) Water uptake of PE18.18-SO_3_H upon storage in water; duplicates were carried out to ensure reproducibility. (b) Recycling to the monomer of PE18.18-SO_3_H-1.0, images display crystallites as obtained from the reaction.

For the acid-containing polyesters PE12.12-SO_3_H and PE18.18-SO_3_H, the absorption of water was investigated to monitor an intrinsically catalyzed polyester chain cleavage. Injection-molded specimens were stored in water for *ca.* 3 months (PE12.12-SO_3_H, 12 weeks and PE18.18-SO_3_H, 10 weeks). The weight gain upon water absorption was monitored on duplicates in parallel over this time, and the decrease in the degree of polymerization was compared (see Figure 6a, data for PE12.12-SO_3_H in the Supporting Information). Note that no water is adsorbed in PE18.18 after an initial small weight gain (0.05 wt %), showing that the incorporated sulfosuccinic acid is accountable for the water uptake. Monitoring the degree of polymerization *via*^1^H NMR spectra (Table S13), the same trend is visible. No major change is observed upon the exposure of PE18.18 to water for 12 and 10 weeks, respectively, whereas a significant decrease in DP_n_ is observed for all sulfonic acid-containing polymers. For PE18.18-SO_3_H, the reduction amounts to ∼50–60% (PE12.12-SO_3_H-1.0 shows a decrease of ∼60%).

It can be concluded that the sulfonic acid groups influence the polymers’ susceptibility to water uptake to a significant extent, thus exposing the incorporated ester groups to water. It is also reasonable to assume that the present acid groups catalyze ester cleavage within the bulk. After the water uptake study, the sulfosuccinate units are still present in the polymers according to ^1^H NMR spectra, which shows that these units are not initially cleaved, washed off, or dissolved and can contribute to the polymer degradation process over a long time period. The reference materials, PE12.12 and PE18.18, do not show significant chain breakdown if exposed to water or humidity on the time scales and under the conditions investigated herein. Thus, a significant impact of the sulfonic acid groups on the hydrolytic degradability of these polyesters is evident.

As reported previously, the long-chain aliphatic polyester PE18.18 can be fully recycled to its monomers in a closed-loop fashion.^29^ The full depolymerization of PE18.18 to its monomers was achieved *via* hydrolysis in water (*ca.* 170 °C) or solvolysis in methanol (*ca.* 120 °C), establishing mild conditions and obtaining highly crystalline monomer mixtures of C_18_-diol and C_18_-diacid (or diester, respectively) in 99% yield. Additionally, repolymerization of the obtained mixture showed that the recycled polymer is on par with the original virgin polymer regarding its thermal as well as mechanical properties. This approach is of interest also for the materials studied in this work, since ion-containing polyethylene-like polymers are typically based on copolymers of ethylene and methacrylic acid, which do not exhibit in-chain functional groups that can be used as breaking points in depolymerizations. Consequently, a recycling-to-monomer approach in ion-containing polymers with polyethylene-like character is lacking to date.

Depolymerization experiments were conducted on PE18.18-SO_3_H-1.0 on a small scale (200 mg of injection-molded polymer sample, solvolysis in methanol, 150 °C, cf. the Supporting Information for details). After cooling to room temperature, a crystallized solid was obtained (Figure 6b). This mixture was recrystallized from methanol once and the resulting crystalline white powder was obtained in 80% yield without further optimization. The monomers are obtained in high purity and in a ratio of 1:0.99 (C_18_-diol/C_18_-diacid) as present in the polymer according to ^1^H NMR spectra. Furthermore, no resonances originating from the sulfosuccinic acid unit are detected, which shows that this minor fraction is removed in the recrystallization process. This is desirable to enable repolymerization to polyesters of different target compositions of sulfonate content (*e.g.*, 1 mol %), though a recovery also of the sulfosuccinate during recycling may also be possible. These results underline the feasibility of complete depolymerization of these ion-containing polymers back to their valuable monomers. A virtually complete cleavage of ester bonds was achieved, and the sulfonic acid was completely removed during the workup process, showing the full recyclability of these polyethylene-like, ion-containing materials.

## Conclusions

Ionic-substituted long-chain polyesters with molecular weights of *M*_n_(GPC) ∼ 50–60 kg/mol are accessible by polycondensation of biobased^29^ long-chain dicarboxylic acids and dimethyl sulfosuccinate with long-chain diols. Despite the anticipated lower reactivity of the sulfosuccinate due to the bulky ionic group, the ionic-substituted repeat units are primarily incorporated in the chains rather than as end groups. Appropriate polycondensation protocols, namely, a limited temperature (160 °C) compared to protocols for the nonionic polyester analogues, avoid any decomposition of the sulfonated monomer or repeat units, respectively. The monomers sulfonic acid groups catalyze the polyesterification without the need for additional catalysts.

Despite the relatively low amounts of ionic repeat units (0.5–1.0 mol %), these can impact the processing and materials properties significantly. Sulfonic acid groups increase the stiffness and decrease the ductility of the materials, presumably due to hydrogen bonding networks. Particularly, they promote water uptake and hydrolytic degradation of the otherwise hydrophobic, stable nonionic analogues. Bivalent counterions result in a significantly increased melt viscosity, which may be beneficial for processing methods requiring high melt stability like film blowing. An increased surface energy also reflects in a much-enhanced adhesion of inks and printability.

By virtue of the in-chain ester groups, the materials can be fully recycled into monomers. While the long-chain monomers are recovered as crystalline materials, the ionic-substituted monomer is separated completely. This is beneficial for reuse of the monomers, to generate desirable compositions of the recycled polymers.

## Supporting Information Available

General remarks and methods; experimental methods; additional GPC, WAXS, ATR-IR, DSC, TGA, tensile testing and rheology data; WCA measurements; NMR spectra of all polymers; and NMR spectrum of the recycling-to-monomer approach (PDF)
