# Supplementary material for: Recyclable and Degradable Ionic-Substituted Long-Chain Polyesters
Source: ACS Sustain Chem Eng. 2023 Aug 9;11(33):12414–22. doi: 10.1021/acssuschemeng.3c03141 (PMC10445281; doi:10.1021/acssuschemeng.3c03141)
Supplement: Supplementary file 1 — sc3c03141_si_001.pdf [file sc3c03141_si_001.pdf]

Supporting Information

Recyclable and Degradable Ionic Substituted Long-Chain Polyesters

*Anne Saumer and Stefan Mecking**

Department of Chemistry, University of Konstanz, Universitätsstraße 10, 78457 Konstanz, Germany

Number of pages: 50

Number of figures: 56

Number of tables: 13

1. Table of Contents

1. Methods 3
   1.1. General 3
   1.2. Determination of DP_n and M_n 5
   1.3. Estimation of the Sulfonic acid Content in PEx.x-SO_3H 7
   1.4. Elemental Analysis 7
   1.5. Amount of Metal Stearate 8
   1.6. TOCSY Results 8
   1.7. Simulation of Sulfonate Distribution 9
   1.8. Recycling to Monomer 12
2. Synthesis 14
   2.1. Synthesis of Polyesters PE12.12-SO_3H-1.0 and PE18.18-SO_3H-x 14
   2.2. Synthesis of Polyesters PE12.12-SO_3M (M = Mg^{2+}, Ca^{2+}, Zn^{2+}) 15
   2.3. Synthesis of Polyesters PE18.18-SO_3M (M = Mg^{2+}, Ca^{2+}, Zn^{2+}) 16
   2.4. Synthesis of Reference Composites Polyesters PE12.12-$M_{stearate}$ 16
   2.5. Processing Conditions 17
3. Additional Figures and Tables 19
   3.1. GPC Data 19
   3.2. ATR-IR Spectra 20
   3.3. X-ray Scattering 21
   3.4. DSC and TGA Thermograms 22
   3.5. Tensile Testing Results 28
   3.6. Water Contact Angle Measurements 32
   3.7. Ink Adsorption on Film Surfaces 33
   3.8. Water Uptake Study 35
   3.9. ^1H NMR Spectra of Polymers PE12.12-SO_3H and PE18.18-SO_3H 36
   3.10. ^1H NMR Spectra of Polymers PE12.12-SO_3M and PE18.18-SO_3M 40
   3.11. ^1H NMR Spectra of Composite Polyesters PE12.12-$M_{stearate}$ 46
   3.12. ^1H NMR Spectrum of Recycling to Monomer 49
4. References 50

1. Methods

1.1. General

Sodium dimethyl sulfosuccinate (SMSS) was synthesized according to a reported procedure.[1] Dimethyl sulfosuccinic (HMSS) acid was synthesized from SMSS by cation exchange in water using Amberlyst IR-120H column. 1,12-Dodecanedioic acid and 1,12-dodecanediol were obtained from tci (> 99 %). 1,18-Octadecanedioic acid was purchased from Elevance Renewable Sciences. 1,18-Octadecanediol was synthesized according to a previously reported procedure.[2] The catalyst [Ti(O^{n}Bu)$_4$] (> 97 %) and the ion exchange resin (Amberlyst IR-120H) were purchased from Merck. Xylene (isomeric mixture, ≥99%) was obtained from Carl Roth. *Iso*-propanol and acetone used for the precipitation and washing procedure of the polymers were used in technical grade.

Deuterated NMR solvents were supplied by Eurisotop.

The characterization of the soluble intermediate products by NMR spectroscopy was performed in chloroform-d_1, 1,1,2,2-tetrachloroethane-d_2 or dimethylsulfoxide-d_6 as solvent at 25 °C or 110 °C, respectively. A Bruker Avance 400 spectrometer was used. ^{1}H chemical shifts were referenced to the residual solvent signal (85%). For spectra evaluation, MestReNova software version 12.0.4 was used. Integration of signals was conducted with the manual multiplet analysis tool.

Molecular weights of the polymers containing metal counterions (PEx.x-SO_3M with M = Na, Mg, Ca, Zn) were determined by gel permeation chromatography on a SECcurity^2 GPC System from PSS with chloroform as solvent at 35 °C and a flow rate of 1 mL/min, using a PSS SDV linear M column and a refractive index detector. The determination of the molecular weight was performed with a linear calibration against narrow polystyrene standards, obtained from Varian. Analysis was carried out using WinGPC UniChrom software (version 8.30).

For differential scanning calorimetry, a Netsch DSC 204 F1 was used. A heating rate of 10 K/min was employed. All data reported are from the second heating cycles. Thermogravimetric analysis was performed on a Netzsch STA 429 F3 Jupiter. All measurements were performed with 80 mL/min flow rate and a heating rate of 10 K/min. For measurements under air, a synthetic 80:20 mixture of N_2:O_2 was used. Samples were dried in a vacuum drying oven for at least 48 h (5 mbar, 50 °C) prior to measurements.

X-ray scattering measurements were performed with a Bruker D8 Avance X-ray diffractometer with scintillation counter, Bruker D8 Discover. As a Cu-K$_\alpha$ source, a Bruker IμS Diamond was

used. As a detector, a Vantec-500 or a Lynxeye XE-T was used. Measured samples were taken from injection molding.

For tensile testing, polymers were processed in a micro compounder (Xplore MC 5). The melt was filled into a micro-injection-moulder (Xplore IM 5.5). The temperature of the cylinder and mould was varied (detailed in section 2.5). An injection pressure of 16 bar (10 s) and 12 bar (15 s) was applied. Tensile tests were performed on a Zwick/005/1446 Retroline tC II instrument, with a crosshead speed of 5 mm/min (ISO 527-2, type 5A). To determine the Young's modulus, a crosshead speed of 1 mm/min was applied. All samples were stored at room temperature for 24 h before tensile tests were performed. For data evaluation, Zwick Roell testXpert software version 11.0 was used.

To determine the water contact angle, samples were taken from injection moulded specimens and washed with *iso*-propanol to remove impurities from the surface. They were measured after storage in a vacuum drying oven for 3 days (50 °C, 50 mbar). The contact angle of a sessile drop of Milli-Q water was determined with a Krüss DSA25 drop shape analyzer using KRÜSS ADVANCE software for data evaluation (version 1.8.0.4).

Rheological data was acquired on an ARES-G2 rheometer (TA instruments) using either 13 mm or 8 mm parallel plates made of Invar. All measurements were performed with an ARES-G2 forced convection oven and flushed with nitrogen during the measurements. Oscillation strain sweeps (γ_0 = 0.1 – 100%) at an angular frequency of 100 rad/s were performed at 140 °C to determine the linear viscoelastic regime, followed by oscillatory frequency sweeps at 160 and 180 °C and a temperature ramp. For the frequency sweeps, a strain amplitude of γ_0 = 10 % was applied. For the temperature ramp, a rate of 10 K/min was applied from 80 to 180 °C with a strain amplitude of γ_0 = 0.1 %. The master curves for PE18.18-SO_3M derivatives were obtained by shifting G' (storage modulus) and G'' (loss modulus) to 180 °C as a reference temperature (by making use of the time-temperature superposition principle). For data acquisition and evaluation, TRIOS software (version 5.5.1.5) was used.

Infrared (IR) spectra were measured on a Perkin Elmer 100 FT-IR spectrometer with an attenuated total reflection (ATR) unit and analyzed with the software Perkin Elmer Inc. (version 6.2.0).

For the water uptake study of PE12.12-SO_3H and PE18.18-SO_3H, injection moulded specimens were immersed in MilliQ water (10 mL). They were weighed on a Sartorius ME36S balance (error: 1 µg). Samples were patted dry, weighed and re-immersed in water between individual measurements. This was repeated until three values were obtained with a deviation of less than 10 µg. A mean value of these three measurements was taken for the data evaluation.

Elemental analysis of polymers PE18.18-SO_3M was carried out by MIKROLAB Kolbe.

For printing on films, an ink-jet printer Canon Pixma TS705 with ink cartridges IC-Office XXL was used. Only the blue color of the cartridge was used, the other printer cartridges were masked to ensure no leakage of their color and to prevent color mixing. The printing was performed on films which were produced with a micro-cast film line. The thicknesses of the films ranged from 50 to 100 µm. The respective films were attached to a Sellotape line on a sheet of paper before introduction to the printer. The logo of the University of Konstanz was printed on the film in blue color. After printing, the films were dried for 24 hours in a drying cabinet at 50 °C. A microfiber cloth was used to wipe the film with even pressure. Similar pressure was applied on the samples to wipe away the design on the films.

1.2. Determination of DP_n and M_n

The degree of polymerization (DP_n) and the number weighted molecular weight (M_n) of the PE12.12-SO_3H-x and PE18.18-SO_3H-x materials were determined by end group analysis from ^1H NMR spectra.

A signal of the backbone of the oligomethylene segment (H-4 and H-7, multiplet at 4.27 – 3.87 ppm) was integrated and referenced to 100H. The end group signals for the hydroxymethyl group (E1, 2H, triplet at 3.63 ppm), the methyl ester (E3 and E3', 3H, singlet at 3.68 ppm) and the acid end group (E2, 2H, triplet at 2.38 ppm) were integrated. In case of the acid end group, the integral cannot be taken directly due to a strong overlap of the signal with the backbone signal H-1. Therefore, only the most downfield shifted and best isolated line of the triplet was integrated and multiplied by a factor of 4. All end groups are assigned representatively for PE18.18-SO_3H-1.0 in **Figure S1**.

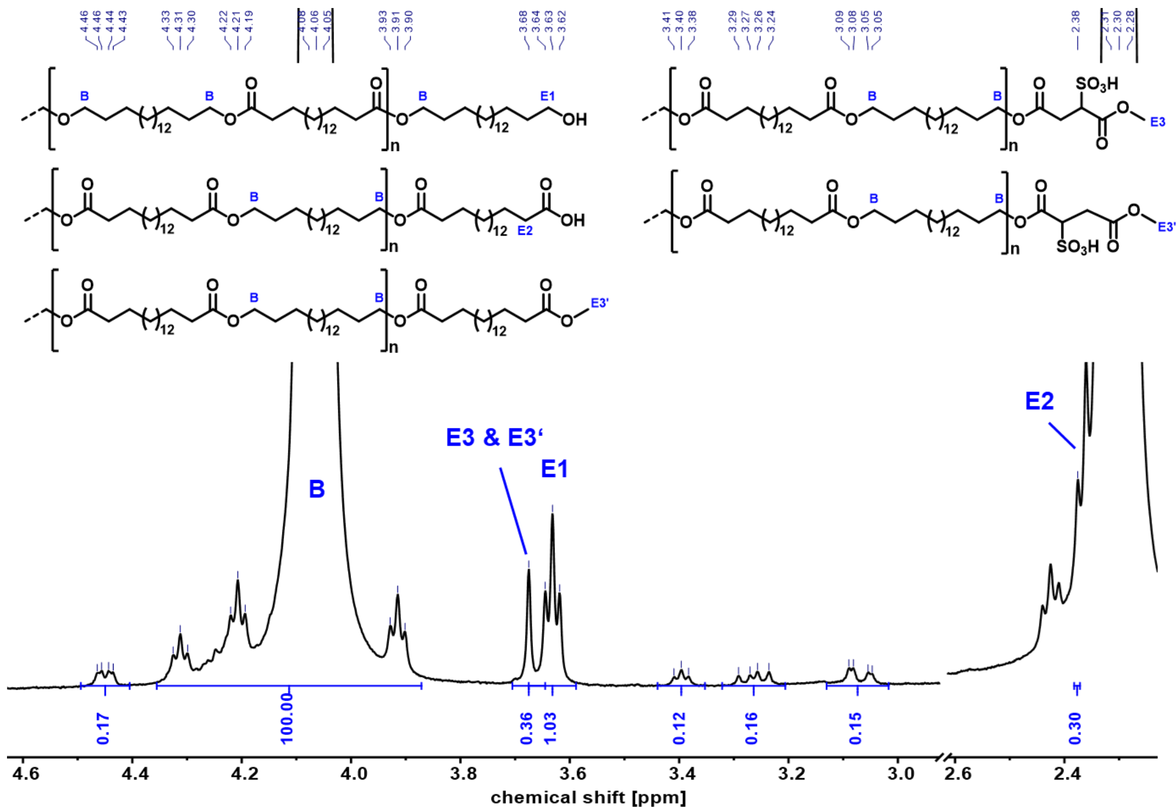

Figure S1 Detailed section of ^{1}H NMR spectra (500 MHz, 323 K, $C_2D_2Cl_4$) of **PE18.18-SO$_3$H-1.0** with end group assignment. Note that the repeat unit can also contain sulfosuccinate units instead of C_{18}-diester units.

The DP_n was calculated according to

$$DP_n = \frac{\int Backbone\ (\mathrm{B})}{\frac{\int E1}{2} + \frac{\int E2}{2} + \frac{\int E3\ \&\ E3'}{3}} + 1 = \frac{100}{\frac{\int E_1}{3} + \frac{\int E_2}{2} + \frac{\int E3\ \&\ E3'}{3}} + 1$$

and the number weighted molecular weights (M_n) were calculated with the DP_n and the molecular weight of the repeat unit (M_{rep})

$$M_n = M_{rep} \times \frac{DP_n}{2}$$

Since the molecular weight differences of the two copolymer repeating units (C_{18}-diol & C_{18}-diester *vs.* C_{18}-diol & sulfosuccinate) are small (**PE12.12-SO$_3$H-1.0:** 397 g/mol *vs.* 364 g/mol; **PE18.18-SO$_3$H-x**: 551 g/mol *vs.* 449 g/mol) and the amounts of sulfonic acid repeating units are low, the molecular weights M_n were calculated from M_{rep} of the non-ionic **PE12.12** or **PE18.18** polymer repeating unit, respectively.

The respective integrals and calculated values are listed in Table S1.

Table S1 Calculation of the DP_n and the M_n of PE12.12-SO_3H-1.0 and PE18.18-SO_3H-x polymers from the end group integrals as obtained from ^{1}H NMR spectra. The integrals of the end groups are referenced to the backbone signal (B), being fixed to 100H.

	∫CH2OH (E1)	∫CH2COOH (E2)	∫COOCH3 (E3 & E3')	DP_n	M_n [kg/mol]
**PE12.12**	0.46	1.12	0.08	123	24.4
**PE12.12-SO3H-1.0**	0.79	1.08	0.39	95	17.3
**PE18.18**	1.13	0.00	1.82	86	23.8
**PE18.18-SO3H-0.5**	1.02	1.28	0.17	84	23.1
**PE18.18-SO3H-0.8**	0.84	1.20	0.31	90	24.8
**PE18.18-SO3H-1.0**	1.03	1.20	0.36	82	22.6

1.3. Estimation of the Sulfonic acid Content in PEx.x-SO_3H

The amount of HMSS incorporated into the polymer was calculated from ^{1}H NMR spectra. It is defined as the molar percentage of sulfosuccinate containing repeat unit in the copolymer. The molar input into the polymerizationreaction mixture is denoted in the polymer nomenclature (PE12.12-SO_3H-1.0 and PE18.18-SO_3H-x, respectively).

The signal of the backbone of the oligoethylene segment (H-4 and H-7, multiplet at 4.27-3.87 ppm) was integrated and referenced to 400H. A mean value of the three protons corresponding to the HMSS unit (δ = 4.45 ppm, H-6; δ = 3.26 ppm, H-5; δ = 3.07 ppm, H-5) was calculated.

The amounts of sulfonic acid determined by analysis of the polymers are generally somewhat lower than the amounts introduced to the polycondensation synthesis (PE12.12-SO_3H-1.0: 0.64 mol%, PE18.18-SO_3H-0.8: 0.57 mol%, PE18.18-SO_3H-1.0: 0.64 mol%), but are conclusive within the expected margin of error. Higher input amounts result in higher incorporated values, and the signals originating from the sulfosuccinate units can be identified in all polymer spectra. Note that for the lowest amount of HMSS input (PE18.18-SO3H-0.5), the estimation of the sulfonic acid content was impeded by the low signal-to-noise ratio.

1.4. Elemental Analysis

Elemental analysis was carried out by MikroLab Kolbe. The samples were dried for 48 h at 50 °C prior to the measurements. Two independent measurements were taken, of which the medium value is listed in Table S2.

Table S2 Results from elemental analysis of polymers PE12.12-SO_3M and PE18.18-SO_3M. C, H and the respective metal cation (M = Mg, Ca, Zn). The theoretical amount of metal (M_{theo}) according to the amount of metal stearate introduced to the pre-polymer is listed.

	**C**	**H**	**M**	**M_{theo}**
**PE12.12-SO_3Mg**	70.81	10.66	0.31	0.26
**PE12.12-SO_3Ca**	71.51	9.92	0.32	0.44
**PE12.12-SO_3Zn**	71.29	10.99	0.72	0.71
**PE18.18-SO_3Mg**	75.26	11.82	0.10	0.10
**PE18.18-SO_3Ca**	75.29	12.00	0.13	0.16
**PE18.18-SO_3Zn**	75.33	11.89	0.28	0.27

1.5. Amount of Metal Stearate

A calculation of the molar amount of stearate present is possible from ^{1}H NMR spectra and accounts to 4 mol% in all PE18.18-SO_3M (2 mol% for PE12.12-SO_3M) with respect to the repeating unit. This corresponds to weight contents of 2 wt.-% for PE18.18-SO_3M and 1.4 wt.-% for PE12.12-SO_3M. All reference composites (PE12.12-$M_{stearate}$) contain ~ 7 mol% of stearate (~ 4 wt.-%), which is higher than the content in the polyesters of interest (PE18.18-SO_3M). Thus, any effects on the material properties originating from the stearates present rather than the incorporated ions should be clearly evident from the reference composites.

1.6. TOCSY Results

Since the Brønstedt acid catalyst (HMSS) can catalyze both, esterification and transesterification, the methyl ester end group can be found on either the HMSS unit or the C_{18}-diacid after the polycondensation procedure (or C_{12}-diacid, respectively). To further investigate the nature of incorporation of the sulfonic-substituted repeat units into the polymer chain vs. endgroups, ^{1}H TOCSY experiments were performed to show that the ^{1}H signals originating from the sulfonic acid containing unit are not correlating with the ester end group at 3.67 ppm (see **Figure S2**).

This experiment shows a correlation of the methylene group (H-5) next to the sulfonic acid containing carbon with the methylene groups in the backbone of the C18-diol (H-3). Also, a clear response from the methylene group of the reacted ester is obtained (H-7). This indicates

that the chosen measurement conditions are sufficient to capture correlations over the ester groups. Regarding the end groups, a very low response from alcohol end-group E2 is obtained, but no clear response from E3 or E3'. This result supports the assumption that transesterification has occurred to a major extent and that the methyl ester end group is mainly attached to the C18-diacid, which is present to a much larger extent than sulfosuccinic acid. This finding underlines the successful synthesis of sulfonic acid containing all-aliphatic polyesters. A statistical distribution of sulfonic acid containing repeat units in the polyester chains can be assumed from this data.

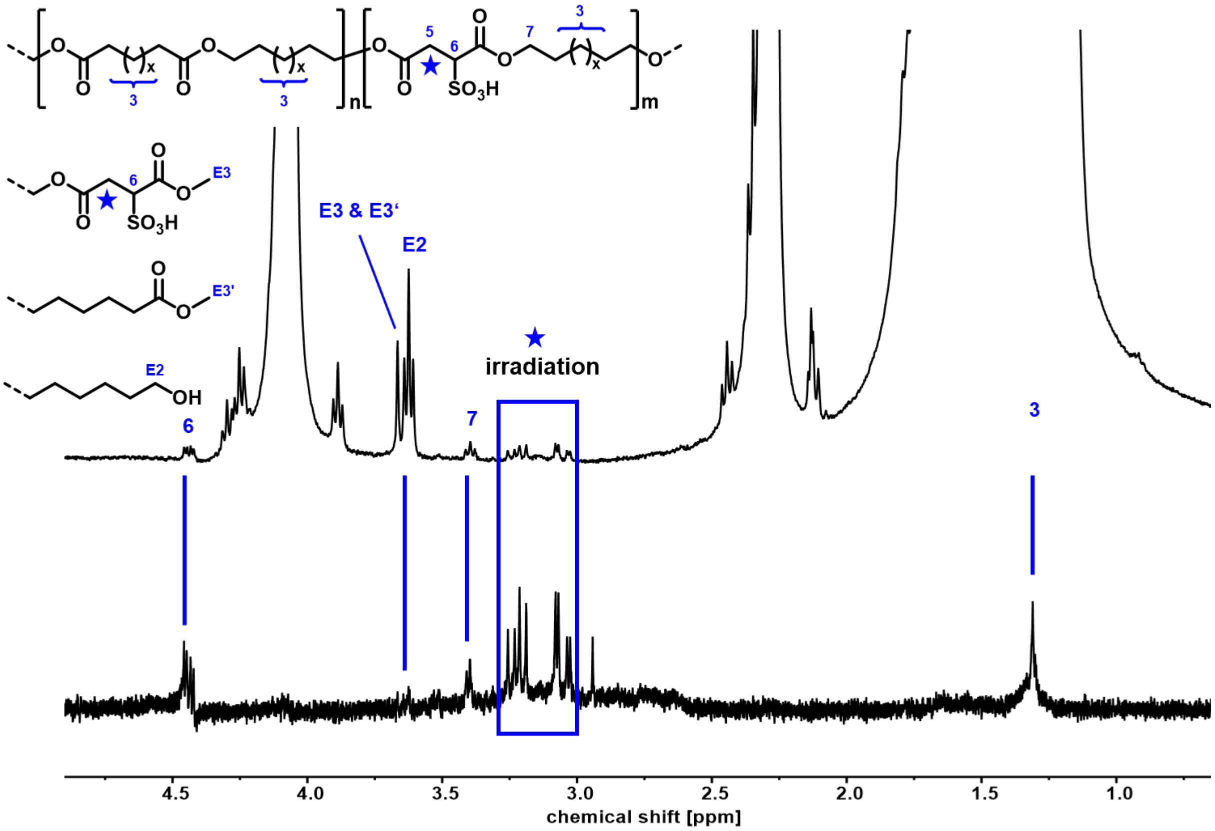

Figure S2 1D Selective gradient TOCSY NMR spectrum (bottom) and ^{1}H NMR spectrum (top, 400 MHz, 383 K, $C_2D_2Cl_4$) of PE18.18-SO_3H-1.0. Irradiation at 3.00 - 3.27 ppm with a mixing time of 250 ms results in a response of H-6, H-7, H-3 but not to a significant extent of E3.

1.7. Simulation of Sulfonate Distribution

To estimate the amount of sulfonate units per polymer chains, a statistical simulation approach was used as previously established by Wu et al.[3], and applied by Odenwald et al.[4] to

acrylate-containing vitrimers. The same calculation method and script as described by Odenwald et al. was used herein.

A Schulz-Flory distribution is assumed in these calculations. Note that for simplicity, a Stirling approximation for large factorials was implemented to limit the amount of numerical calculations.

Considering that the DP_n values of PE18.18-SO_3H-x are ~80-90 (defined as the amount of monomers connected), and the input amounts of sulfosuccinic acid monomers are low (0.5, 0.8 and 1.0 mol% with regard to overall diol), it is expected a relevant fraction of formed polymer chains contains no incorporated sulfonate unit, in addition to chains with one, or several sulfonate units.

For the calculation, the degree of polymerization and the molar amount of sulfosucccinic acid employed in the synthesis of the PE18.18-SO_3H-x polymers were considered. First, a fully statistical incorporation of the sulfosuccinic acid within the polymer chains without any end group enrichment or depletion was considered (**Figure S3**).

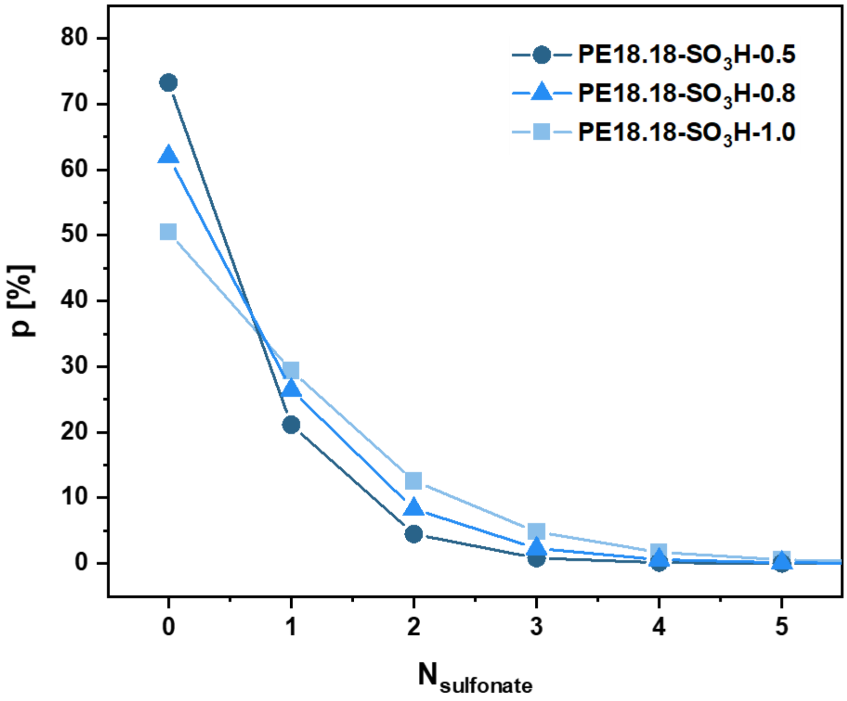

Figure S3 Statistical distribution of the amount of sulfonate groups per polymer chain ($N_{sulfonate}$) and the corresponding probability (p).

In case of PE18.18-SO_3H-0.8, ~ 62 % of the polymer chains are unfunctionalized. As this material is the basis for the PE18.18-SO_3M materials, an alternative calculation under consideration of the end group enrichment of the sulfosuccinate unit was performed.

Note that a full transesterification is assumed for the following calculations, meaning that the methyl ester end groups detected in the ^{1}H NMR spectra are placed to 99.2 % at the C_{18}-dioate and 0.8 % are placed at the sulfosuccinate unit. This assumption is supported by the TOCSY results discussed above (section 1.5).

For this purpose, a detailed analysis of the ester groups from ^{1}H NMR spectra is required (see **Figure S4**). A successful esterification of sulfosuccinate with C_{18}-diol is visible from the signal H-7 (3.40 ppm). A full incorporation into the chain would mean a 2-fold integral opposed to H-5. From the ratio I(H-7)/(2*I(H-5)), the amount of esterification of one end of the sulfosuccinate can be calculated. A successful esterification of other end results in a signal (H-7') that overlaps with the backbone signal (B). From the intensity, a full incorporation can be assumed, as this side is much more reactive since the sulfonic acid group is placed in *β*-position to the reactive ester.

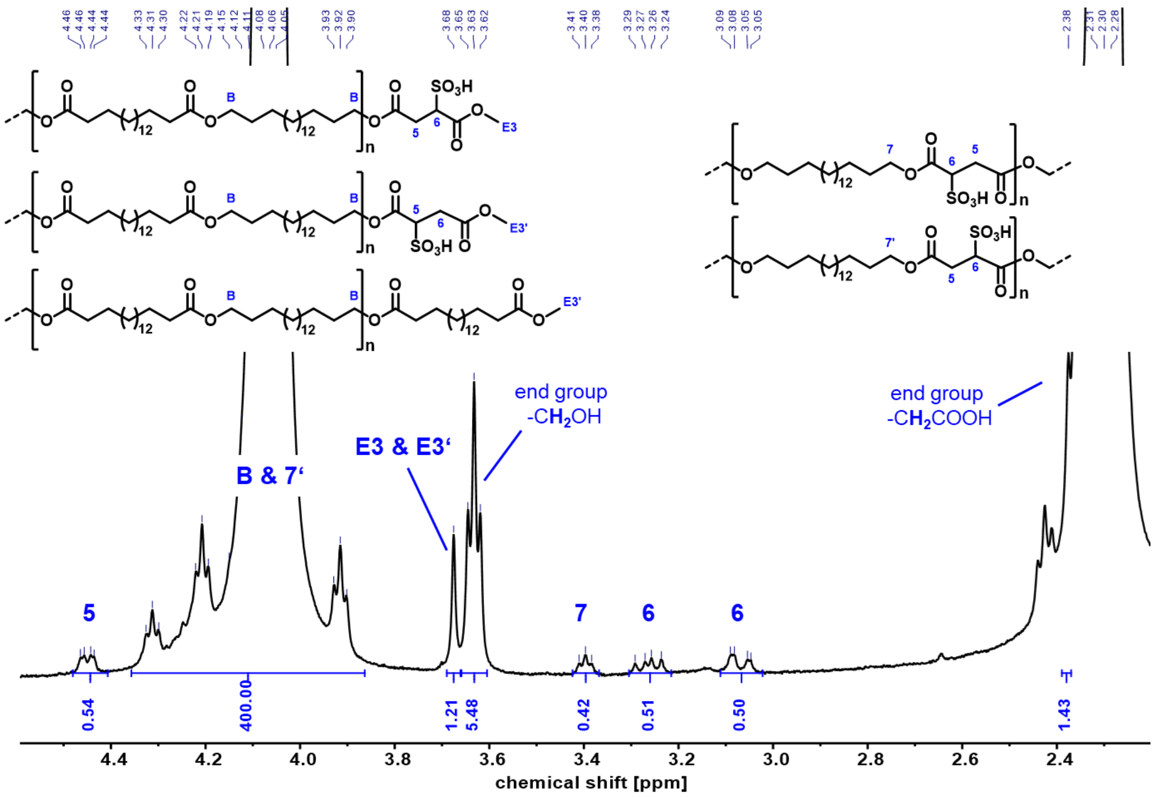

Figure S4 ^{1}H NMR spectrum (500 MHz, 323 K, $C_2D_2Cl_4$) of **PE18.18-SO3H-0.8** with assignment of the end groups and the signals deriving from the sulfosuccinate unit.

From these incorporations (accounting for a total of 71% incorporation of the sulfosuccinate ester groups), and the total amounts of acid and ester end groups, the probabilities to obtain a sulfonate ester end group was calculated. A value of 6.7 % of the ester and acid end groups

were found to derive from the sulfosuccinate. Upon consideration of the probability to find an acid or ester end group rather than an alcohol end group, it was found that 3.7 % of the total amount of end groups are sulfosuccinate end groups.

From this result, the probabilities of zero, one or two sulfosuccinate end groups were calculated with

$$p_0 = (1 - 0.037) \times (1 - 0.037)$$

$$p_1 = 2 \times 0.037 \times (1 - 0.037)$$

$$p_2 = 0.037 \times 0.037$$

These values were used to calculated the end group enrichment according to the method used by Odenwald et al., the results are shown graphically in **Figure S5**. The probability (p) depending on the number of sulfosuccinate units found in a polymer chain ($N_{sulfonate}$).

It can be seen that the differences between an entirely statistical incorporation and an end group enrichment is marginal.

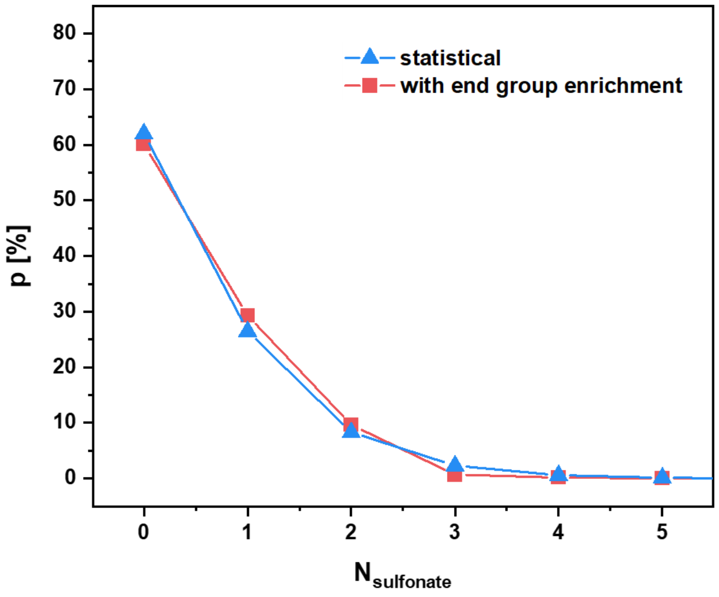

Figure S5 Probability (p) of N sulfonate groups per polymer chain with and without consideration of end group enrichment.

1.8. Recycling to Monomer

For the recycling to monomer of PE18.18-SO_3H-1.0, a stainless-steel reactor with a glass inlet (total volume of 20 mL) was charged with 200 mg of injection moulded specimen and methanol

(8 mL, > 99.8 % purity) as well as a stir bar. The reaction mixture was heated to 150 °C for 6 days and stirred at 300 rpm, upon which a pressure of 12 bar developed. After cooling to room temperature without further stirring, a crystallized solid was obtained. The supernatant was removed, and methanol (5 mL) was added. The mixture was heated to reflux until a clear solution was obtained and subsequently was cooled to 4 °C. The crystallized solid was centrifuged off, washed with methanol and dried. A 1:0.99 mixture of C_{18}-diol:C_{18}-diester was obtained according to the ^{1}H NMR spectrum (*cf.* section 3.11, **Figure S56**) in 80 % yield.

2. Synthesis

2.1. Synthesis of Polyesters PE12.12-SO_3H-1.0 and PE18.18-SO_3H-x

Different polymers were synthesized according to the same synthesis procedure, where the molar amount of dimethyl sulfosuccinic acid (HMSS) was varied (as listed in **Table S3**). The total amount of diester/diacid (HMSS and 1,18-octadecanedioic acid, or 1,12-dodecanedioic acid, respectively) accounts for one equivalent with respect to the used 1,18-dodecanediol (or 1,12-dodecanediol, respectively).

Table S3 Molar feed ratio in polycondensation reactions of PE12.12-SO_3H-x and PE18.18-SO_3H-x.

	diol	diacid	HMSS
**PE12.12**	1.00	1.00	0.00
**PE12.12-SO₃H-1.0**	1.00	0.99	0.01
**PE18.18**	1.00	1.00	0.00
**PE18.18-SO₃H-0.5**	1.00	0.995	0.005
**PE18.18-SO₃H-0.8**	1.00	0.992	0.008
**PE18.18-SO₃H-1.0**	1.00	0.99	0.01

For the synthesis of PE18.18-SO_3H, 1,18-octadecanediol (10 g, 34.90 mmol, 1.0 eq.), dimethyl sulfosuccinic acid and 1,18-octadecanedioic acid were weighed into a 250 mL round bottom flask equipped with a distillation bridge and a collecting flask. To stir the mixture, an elliptical PTFE coated stir bar with a rare earth magnetic core was used. The reaction mixture was heated to 145 °C and stirred at 350 rpm in a 250 mL sandwich-type aluminum block, so that the flask was heated up to the top. The collecting flask was cooled with liquid nitrogen and connected to a membrane pump. Within 3 hours, the pressure was gradually reduced from 900 mbar to 50 mbar, upon which the viscosity increased significantly. The stirring speed was reduced at the same time, until the stir bar no longer moved. The membrane pump was replaced by an oil pump, and vacuum (5×10^{-3} mbar) was applied for another 2 h. The obtained polymer was isolated by scratching from the flask in the melt state.

The same procedure was followed for polycondensation to PE12.12-SO_3H-1.0, with 1,12-dodecanediol (13 g, 64.26 mmol, 1.00 eq.), HMSS (0.01 eq.) and 1,12-dodecanedioic acid (0.99 eq.).

For PE12.12 and PE18.18, the same procedure was applied with the use of 0.3 mol% of [Ti(O^{n}Bu)$_4$] as catalyst. The reaction time at a vacuum of 5 x 10^{-3} mbar was 12 hours.

All polymers containing free sulfonic acid were stored in a glovebox under a nitrogen atmosphere.

Assignment of ^{1}H NMR signals according to Figure S30 - Figure S37.

PE12.12 ^{1}H NMR (400 MHz, $C_2D_2Cl_4$, 323 K) δ [ppm] = 4.11 (t, $^3J_{HH}$ = 6.7 Hz, H-4 and H-4‘), 3.74 and 3.71 (s, E3 and E3’), 3.67 (t, $^3J_{HH}$ = 6.5 Hz, E1), 2.66 (s, H-5), 2.39 (t, $^3J_{HH}$ = 7.4 Hz, E2), 2.33 (t, $^3J_{HH}$ = 7.4 Hz, H-1), 1.79 – 1.60 (m, H-2 and H-2‘), 1.56 – 1.31 (m, H-3).

PE12.12-SO$_3$H-1.0 ^{1}H NMR (400 MHz, $C_2D_2Cl_4$, 323 K) δ [ppm] = 4.45 (dd, $^3J_{HH}$ = 10.5, 4.2 Hz, H-6), 4.31 (t, $^3J_{HH}$ = 6.7 Hz, H-7) 4.28 – 3.82 (m, H-4), 3.69 & 3.67 (s, E3 and E3‘), 3.63 (t, $^3J_{HH}$ = 6.6 Hz, E1), 3.39 (t, $^3J_{HH}$ = 6.7 Hz, S1), 3.26 (dd, $^3J_{HH}$ = 17.3, 10.5 Hz, H-5), 3.07 (dd, $^3J_{HH}$ = 17.3, 4.2 Hz, H-5), 2.36 (t, $^3J_{HH}$ = 7.4 Hz, E2), 2.30 (t, $^3J_{HH}$ = 7.5 Hz, H-1), 1.70 – 1.54 (m, H-2 and H-2‘), 1.42 – 1.20 (m, H-3).

PE18.18-SO$_3$H-0.8 ^{1}H NMR (500 MHz, $C_2D_2Cl_4$, 323 K) δ [ppm] = 4.45 (dd, $^3J_{HH}$ = 10.6, 4.0 Hz, H-6), 4.31 (t, $^3J_{HH}$ = 6.8 Hz, H-7), 4.27 – 3.85 (m, H-4), 3.68 (s, E3 & E3‘), 3.63 (t, $^3J_{HH}$ = 6.6 Hz, E1), 3.40 (t, $^3J_{HH}$ = 6.6 Hz, S1), 3.26 (dd, J = 17.4, 10.6 Hz, H-5), 3.07 (dd, J = 17.4, 4.0 Hz, H-5), 2.38 (t, $^3J_{HH}$ = 7.4 Hz, E2), 2.46 – 2.14 (m, H-1), 1.71 – 1.53 (m, H-2 and H-2‘), 1.51 – 1.11 (m, H-3).

2.2. Synthesis of Polyesters PE12.12-SO$_3$M (M = Mg^{2+}, Ca^{2+}, Zn^{2+})

For the synthesis of PE12.12-SO$_3$M, the same polycondensation procedure as for PE12.12-SO$_3$H-1.0 was followed. A reaction scale of 50.00 g 1,12-dodecanediol (247 mmol, 1.00 eq.), 56.34 g 1,12-dodecanedioic acid (245 mmol, 0.99 eq.) and 0.56 g HMSS (2.5 mmol, 0.01 eq.) was used. When the viscosity reached a limit where no more melt flow was observed, the polymer was dissolved in xylene (250 mL) at 130 °C. After a homogeneous solution was obtained, the batch was divided into three parts (by weight). To each aliquot, a solution of zinc, calcium or magnesium stearate, respectively, in xylene (50 mL) was added (3.71 mmol, 0.015 eq.). The mixtures were stirred for 10 min and precipitated in cold iPrOH (-30 °C). After filtration, the polymers were washed with acetone and dried in a vacuum drying oven for three days.

Assignment of ^{1}H NMR signals according to Figure S38 - Figure S42.

PE12.12-SO_3Mg ^1H NMR (500 MHz, $C_2D_2Cl_4$, 323 K) δ [ppm] = 4.45 (dd, $^3J_{HH}$ = 10.5, 4.2 Hz, H-6), 4.34 – 3.87 (m, H-4), 3.67 (s, *end groups* E3 or E3'), 3.63 (t, $^3J_{HH}$ = 6.7 Hz, *end group* E1), 3.39 (t, $^3J_{HH}$ = = 6.7 Hz, H-7), 3.26 (dd, $^3J_{HH}$ = 17.3, 10.5 Hz, H-5), 3.07 (dd, $^3J_{HH}$ = 17.3, 4.2 Hz, H-5), 2.32 (t, $^3J_{HH}$ = 5.4 Hz, *stearate* S1 and *end group* E2), 2.30 (t, $^3J_{HH}$ = 7.6 Hz, H-1), 1.68 – 1.58 (m, H2 & H2'), 1.40 – 1.25 (m, H-3), 0.91 (t, $^3J_{HH}$ = 6.8 Hz, *stearate* S2).

2.3. Synthesis of Polyesters PE18.18-SO_3M (M = Mg^{2+}, Ca^{2+}, Zn^{2+})

For the synthesis of PE18.18-SO_3M, the same polycondensation procedure as for PE18.18-SO_3H was followed. A reaction scale of 45 g 1,18-octadecanediol (157 mmol, 1.00 eq.), 49.00 g 1,18-octadecanedioic acid (156 mmol, 0.992 eq.) and 0.28 g HMSS (1.3 mmol, 0.008 eq.) was used. When the viscosity reached a limit where no more melt flow as observed, the polymer was dissolved in xylene (350 mL) at 130 °C. After a homogeneous solution was obtained, the batch was divided into three parts (by weight). To each aliquot, a solution of zinc, calcium or magnesium stearate, respectively, in xylene (50 mL) was added (1.3 mmol, 0.008 eq.). The mixtures were stirred for 10 min and precipitated in cold iPrOH (-30 °C). After filtration, the polymers were washed with acetone and dried in a vacuum drying oven for three days.

Assignment of ^1H NMR signals according to Figure S44 - Figure S48.

PE18.18-SO_3Mg ^1H NMR (500 MHz, $C_2D_2Cl_4$, 323 K) δ [ppm] = 4.45 (dd, $^3J_{HH}$ = 10.5, 4.2 Hz, H-6), 4.3 – 3.89 (m, H-4), 3.70 & 3.68 (s, *end groups* E3 & E3'), 3.63 (t, $^3J_{HH}$ = 6.7 Hz, *end group* E1), 3.39 (t, $^3J_{HH}$ = 6.7 Hz, H-7), 3.26 (dd, $^3J_{HH}$ = 17.3, 10.5 Hz, H-5), 3.07 (dd, $^3J_{HH}$ = 17.3, 4.2 Hz, H-5), 2.38 (t, $^3J_{HH}$ = 6.7 Hz, *stearate* S1 and *end group* E2), 2.30 (t, $^3J_{HH}$ = 7.5 Hz, H-1), 1.70 – 1.55 (m, H 2 & H2'), 1.41 – 1.17 (m, H -), 0.91 (t, , $^3J_{HH}$ = 6.8 Hz, *stearate* S2).

2.4. Synthesis of Reference Composites Polyesters PE12.12-$M_{stearate}$

For the synthesis of PE12.12-$M_{stearate}$, 1,12-dodecanediol (50.00 g, 247 mmol, 1.00 eq.), 1,12-dodecanedioic acid (56.35 g, 245 mmol, 0.99 eq.) and dimethyl succinate (0.36 g, 2.47 mmol, 0.01 eq.) were weighed into a 1 L round bottom flask equipped with a distillation bridge and a collecting flask. The reaction mixture was degassed, and the collecting flask cooled with liquid nitrogen. To stir the mixture, an elliptical PTFE coated stir bar with a rare earth magnetic core was used. The reaction mixture was heated to 145 °C and stirred at 350 rpm in a 250 mL sandwich-type aluminum block, so that the flask was heated up to the top. When a

homogeneous melt was obtained, the catalyst [Ti(O^{n}Bu)$_4$] (0.25 g, 0.74 mmol, 0.3 mol%) was added and the collecting flask was connected to a membrane pump. Within 3 hours, the pressure was gradually reduced from 900 mbar to 50 mbar, upon which the viscosity increased significantly. The stirring speed was reduced at the same time, until the stir bar no longer moved. The membrane pump was replaced by an oil pump, and vacuum (5 x 10^{-3} mbar) was applied for another 36 h, until a sufficiently high melt viscosity was reached. The polymer was dissolved in xylene (200 mL) at 130 °C until a homogeneous solution as obtained. The batch was divided into three equally sized parts by weight, and to each part, a solution of zinc, calcium or magnesium stearate, respectively, in xylene (50 mL) was added (3.7 mmol, 0.015 eq.). The mixtures were stirred for 10 min at 130 °C and precipitated in cold iPrOH (-30 °C). After filtration, the polymers were washed with acetone and dried in a vacuum drying oven for three days.

Assignment of ^{1}H NMR signals according to Figure S50 - Figure S54.

PE12.12-Mg$_{\text{stearate}}$ ^{1}H NMR (500 MHz, $C_2D_2Cl_4$, 323 K) δ [ppm] = 4.06 (t, $^3J_{HH}$ = 6.8 Hz, H-4 & H-4’), 3.71 & 3.67 (s, end groups E3 & E3’), 3.63 (t, $^3J_{HH}$ = 6.6 Hz, *end group* E1), 2.63 & 2.62 (s, H-5) 2.36 (t, $^3J_{HH}$ = 7.2 Hz, *stearate* S1 and *end group* E2), 2.30 (t, $^3J_{HH}$ = 7.5 Hz, H-1), 1.68 – 1.58 (m, H2 & H2’), 1.40 – 1.25 (m, H-3), 0.91 (t, $^3J_{HH}$ = 6.8 Hz, *stearate* S2).

2.5. Processing Conditions

All polymers were processed in a micro-compounder to homogenize the polymer melt and were subsequently injection-moulded into test specimens for tensile testing (ISO 527-2, type 5A). At least five test specimens were prepared for each polymer. The temperature of the micro-compounder (T_{melt}) and of the injection mould (T_{mould}) were set to different values depending on the nature of the polymer. An overview of the processing conditions is listed in **Table S4**. The stirring speed was set to 10 rpm and the mixing time before the first extrusion to homogenize the melt was 10 min in all cases.

Table S4 Conditions for processing of the ion-containing polyesters.

	T_{melt} [°C]	T_{mould} [°C]
**PE12.12-SO_3H-1.0**	160	55
**PE18.18-SO_3H-x**	180	60
**PE12.12-SO_3M-x**	160	55
**PE18.18-SO_3M-x**	180	60

Film drawing employed a micro-cast film line, with varying speed of the film line to obtain different film thicknesses. A film drawing was possible with all polymers PE12.12-SO_3M and PE18.18-SO_3M, as well as with the reference polymers PE12.12, PE18.18 and HDPE. For PE12.12-$M_{stearate}$, films were only possible to draw for PE12.12-$Mg_{stearate}$ and PE12.12-$Ca_{stearate}$. The melt viscosity of PE12.12-$Zn_{stearate}$ was too low, and no continuous film could be drawn.

3. Additional Figures and Tables

3.1. GPC Data

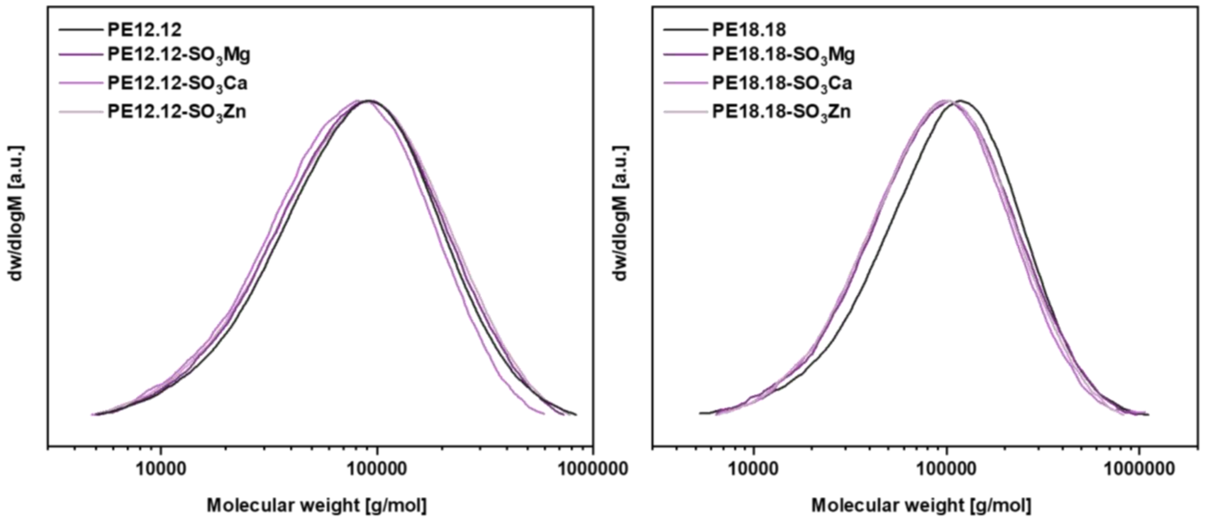

Figure S6 GPC traces of PE12.12-SO_3M (left) and PE18.18-SO_3M (right) and their reference polymers PE12.12 and PE18.18, respectively. Linear calibration against PS standards was applied ($CHCl_3$, 35 °C).

Table S5 Number average molecular weights (M_n) and polydispersity indexes (Đ) as determined by GPC analysis.

	**M_n [kg/mol]**	**Đ**		**M_n [kg/mol]**	**Đ**
**PE12.12**	53	2.2	**PE18.18**	68	2.1
**PE12.12-SO_3Mg**	52	2.1	**PE18.18-SO_3Mg**	61	2.1
**PE12.12-SO_3Ca**	48	2.0	**PE18.18-SO_3Ca**	61	2.2
**PE12.12-SO_3Zn**	52	2.2	**PE18.18-SO_3Zn**	61	2.1

GPC measurements were performed in chloroform at 35 °C, refractive index detection. Linear calibration against polystyrene applied.

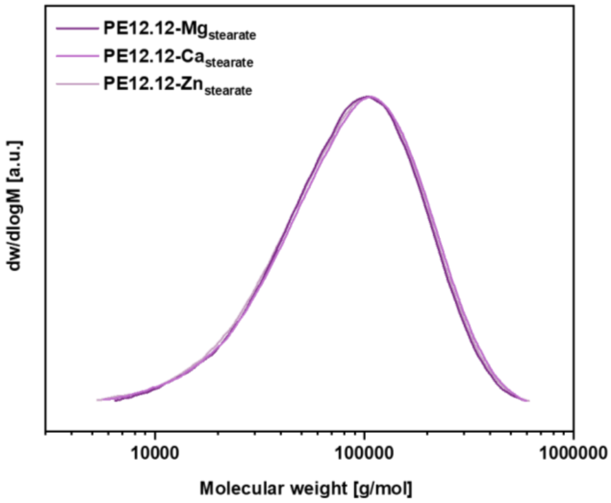

Figure S7 GPC traces of reference composites PE12.12-$M_{stearate}$. Linear calibration against PS standards was applied.

Table S6 Number average molecular weights (M_n) and polydispersity indexes (Đ) of reference composites PE12.12-$M_{stearate}$ as determined by GPC analysis.

	M_n [kg/mol]	Đ
**PE12.12-Mg$_{stearate}$**	61	1.8
**PE12.12-Ca$_{stearate}$**	60	1.9
**PE12.12-Zn$_{stearate}$**	58	1.9

3.2. ATR-IR Spectra

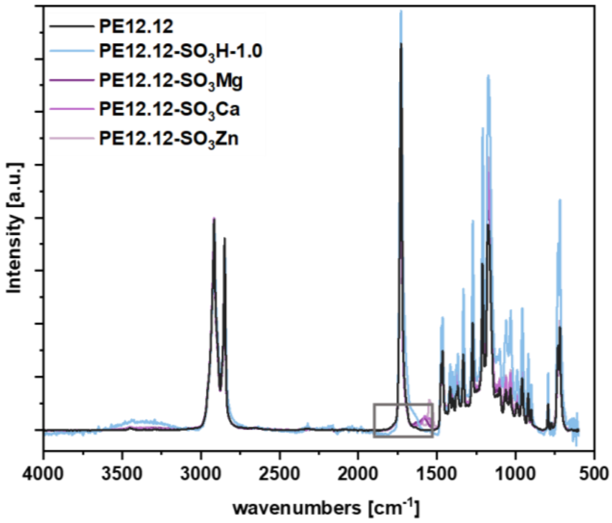

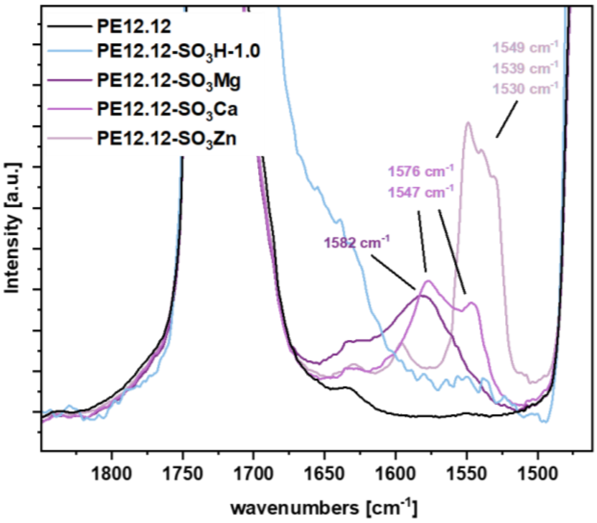

Figure S8 ATR-IR spectra of PE12.12, PE12.12-SO_3H-1.0 and PE12.12-SO_3M. Left graph shows full range; right graph shows the grey marked carbonyl region.

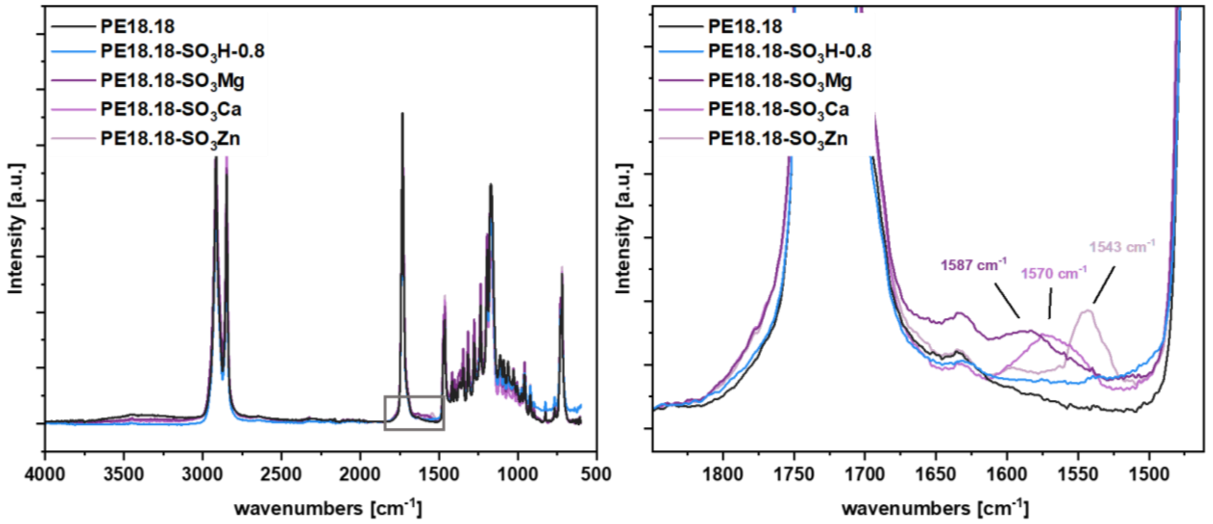

Figure S9 ATR-IR spectra of PE18.18, PE18.18-SO_3H-0.8 and PE18.18-SO_3M. Left graph shows full range; right graph shows the grey marked carbonyl region.

3.3. X-ray Scattering

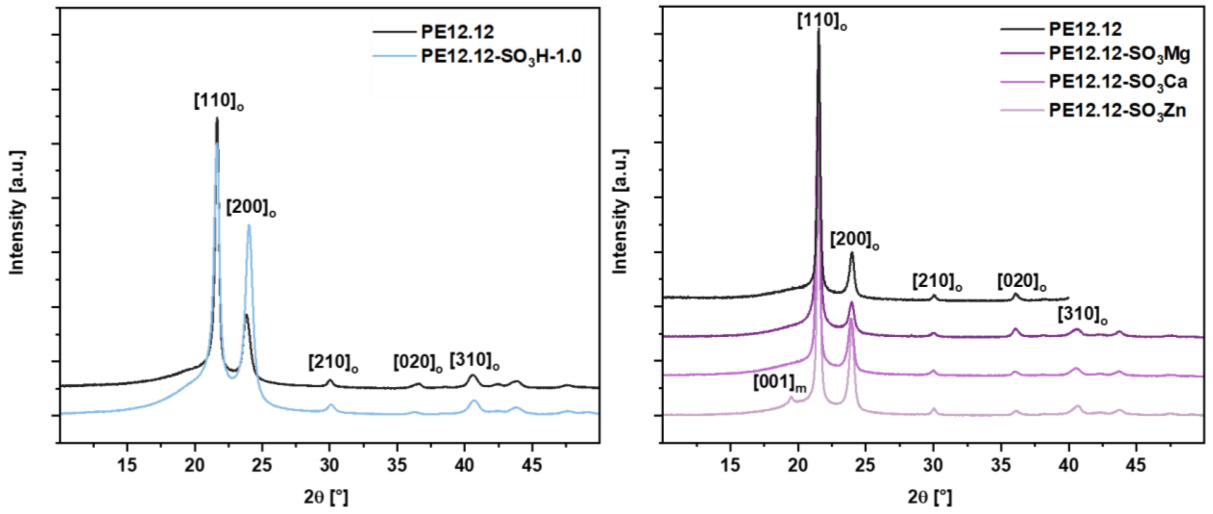

Figure S10 X-ray scattering profiles of PE12.12-SO_3H-1.0 (left) and PE12.12-SO_3M (right) and their corresponding non-ion containing polyester PE12.12.

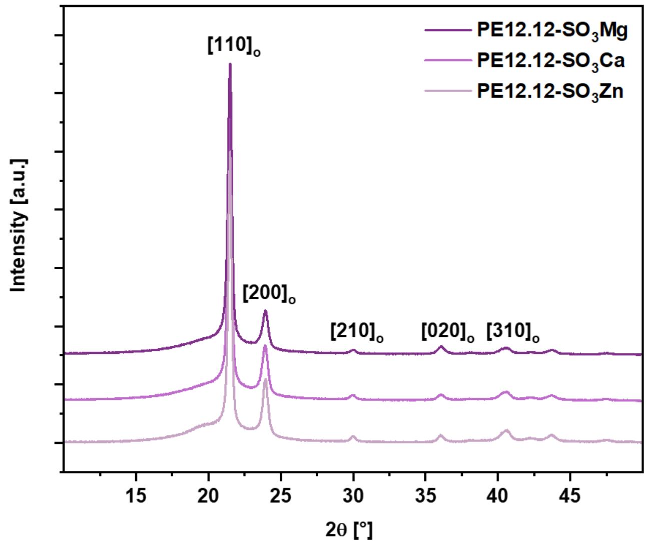

Figure S11 X-ray scattering profiles of PE12.12-$M_{stearate}$.

3.4. DSC and TGA Thermograms

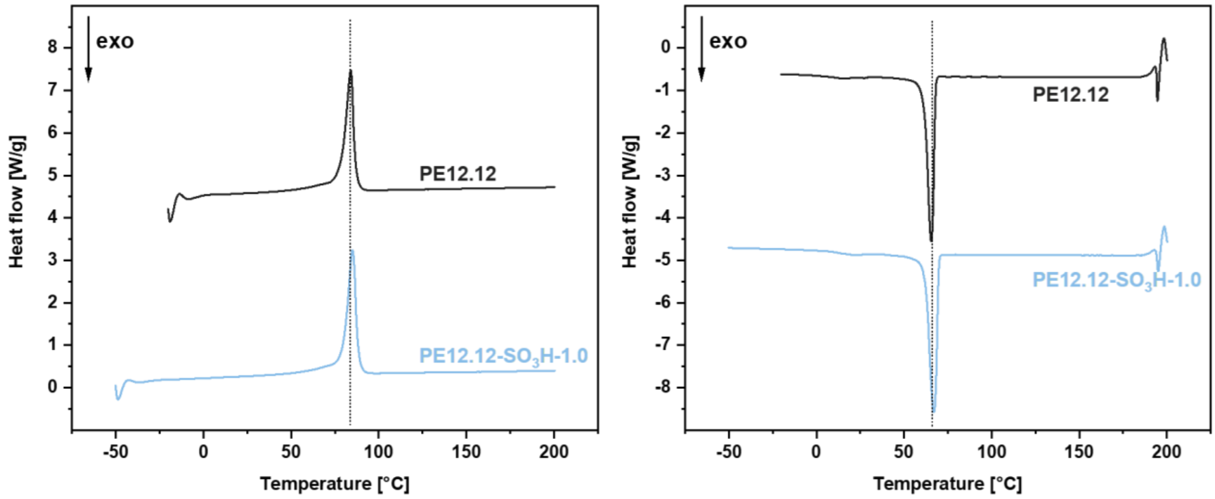

Figure S12 DSC thermograms of PE12.12 and PE12.12-SO_3H-1.0 upon heating (left) and cooling (right). Data from the second cycle with a heating/cooling rate of 10 K/min are displayed. Data was shifted vertically for clarity.

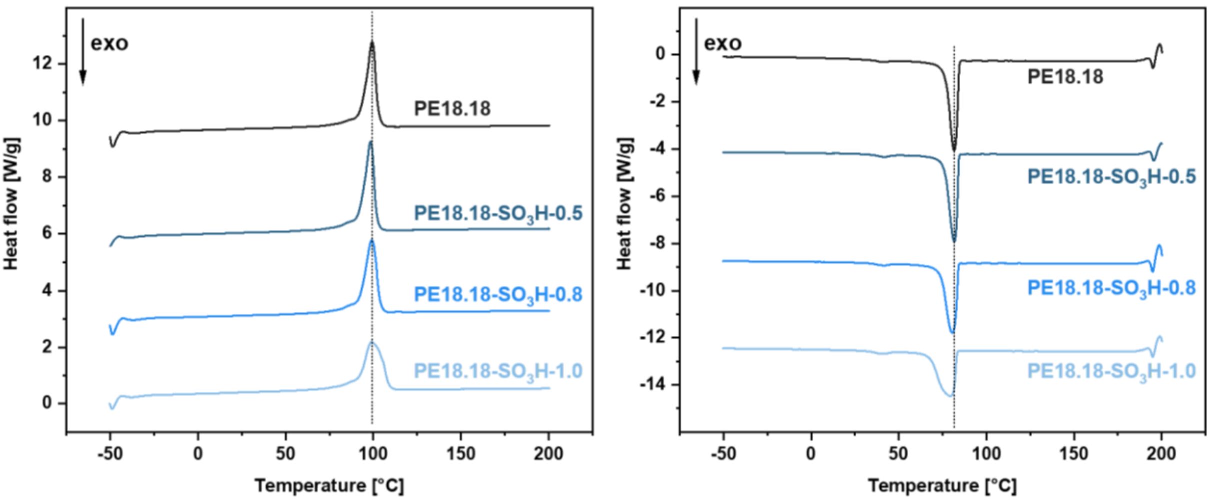

Figure S13 DSC thermograms of PE18.18 and PE18.18-SO_3H upon heating (left) and cooling (right). Data from the second cycle with a heating/cooling rate of 10 K/min are displayed. Data was shifted vertically for clarity.

Table S7 Thermal properties of PE12.12-SO_3H-1.0 and PE18.18-SO_3H polymers as obtained from DSC measurements. Peak melting temperatures (T_m), enthalpies of fusion (ΔH_m), peak crystallization temperatures (T_c) and enthalpies of crystallization (ΔH_c) are listed. Data were taken from the second heating/cooling cycle (10 K/min heating/cooling rate).

	T_m [°C]	ΔH_m [J/g]	T_c [°C]	ΔH_c [J/g]
**PE12.12**	84.9	136	66.2	-114
**PE12.12-SO_3H-1.0**	85.0	136	66.9	-126
**PE18.18**	99.4	145	81.6	-131
**PE18.18-SO_3H-0.5**	98.4	151	81.2	-124
**PE18.18-SO_3H-0.8**	99.1	139	80.4	-124
**PE18.18-SO_3H-1.0**	99.1	142	79.5	-127

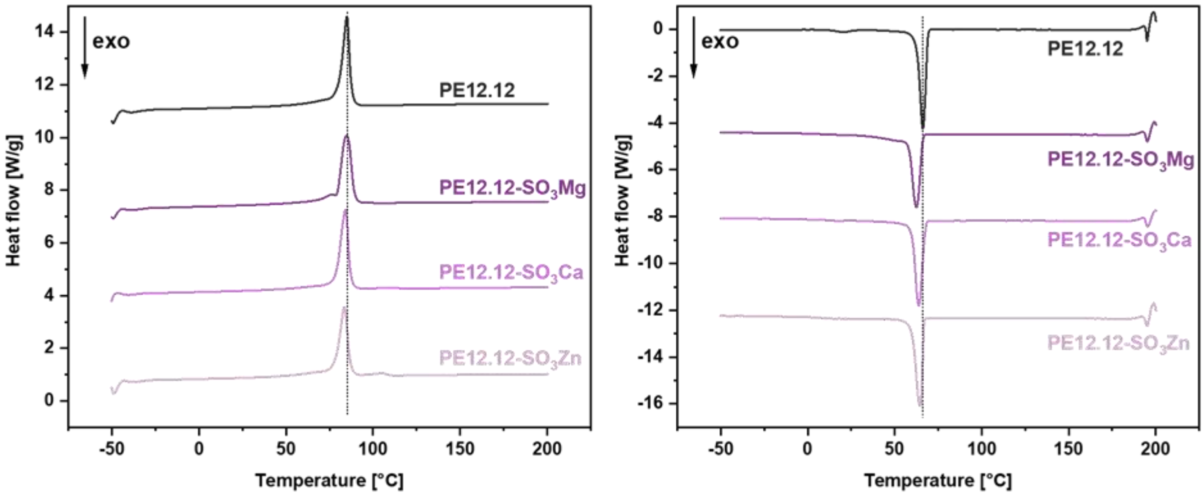

Figure S14 DSC thermograms of PE12.12 and PE12.12-SO3M upon heating (left) and cooling (right). Data from the second cycle with a heating/cooling rate of 10 K/min are displayed. Data was shifted vertically for clarity.

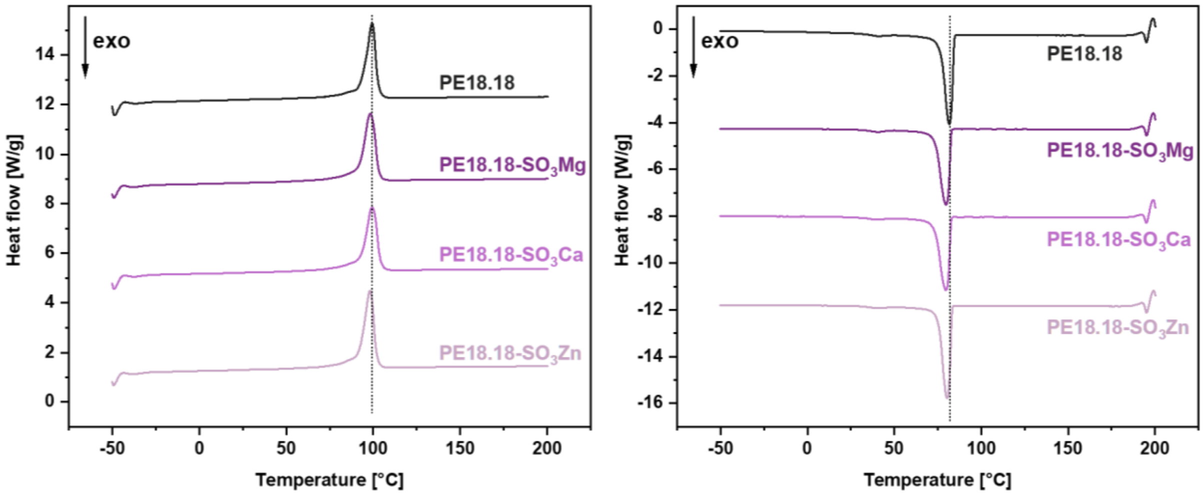

Figure S15 DSC thermogram of PE18.18 and PE18.18-SO3M upon heating (left) and cooling (right). Data from the second cycle with a heating/cooling rate of 10 K/min are displayed. Data was shifted vertically for clarity.

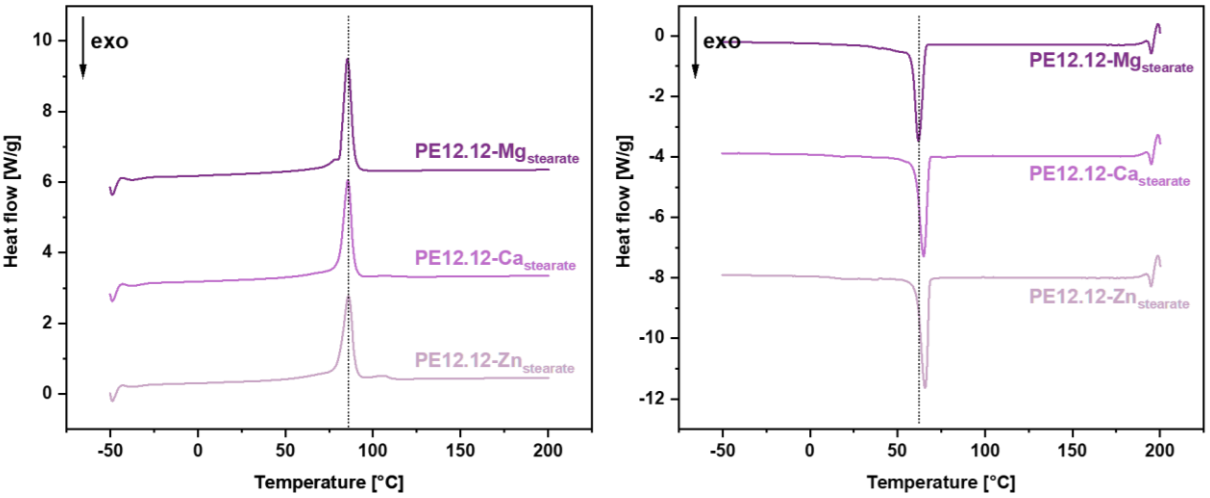

Figure S16 DSC thermogram of PE12.12-$M_{stearate}$ upon heating (left) and cooling (right). Data from the second cycle with a heating/cooling rate of 10 K/min are displayed. Data was shifted vertically for clarity.

Table S8 Thermal properties of PE12.12-SO_3M, PE12.12-$M_{stearate}$ and PE18.18-SO_3M polymers as obtained from DSC measurements. Peak melting temperatures (T_m), enthalpies of fusion (ΔH_m), peak crystallization temperatures (T_c) and enthalpies of crystallization (ΔH_c) are listed. Data were taken from the second heating/cooling cycle (10 K/min heating/cooling rate).

	T_m [°C]	ΔH_m [J/g]	T_c [°C]	ΔH_c [J/g]
**PE12.12**	84.9	136	66.2	-114
**PE12.12-SO_3Mg**	84.8	134	62.4	-115
**PE12.12-SO_3Ca**	84.1	122	63.8	-113
**PE12.12-SO_3Zn**	83.5	111	64.5	-104
**PE12.12-$Mg_{stearate}$**	85.4	127	62.6	-94
**PE12.12-$Ca_{stearate}$**	85.6	115	64.9	-106
**PE12.12-$Zn_{stearate}$**	85.3	108	65.8	-106
**PE18.18**	99.4	145	81.6	-131
**PE18.18-SO_3Mg**	98.3	151	79.6	-121
**PE18.18-SO_3Ca**	99.4	142	79.9	-116
**PE18.18-SO_3Zn**	98.1	151	80.3	-122

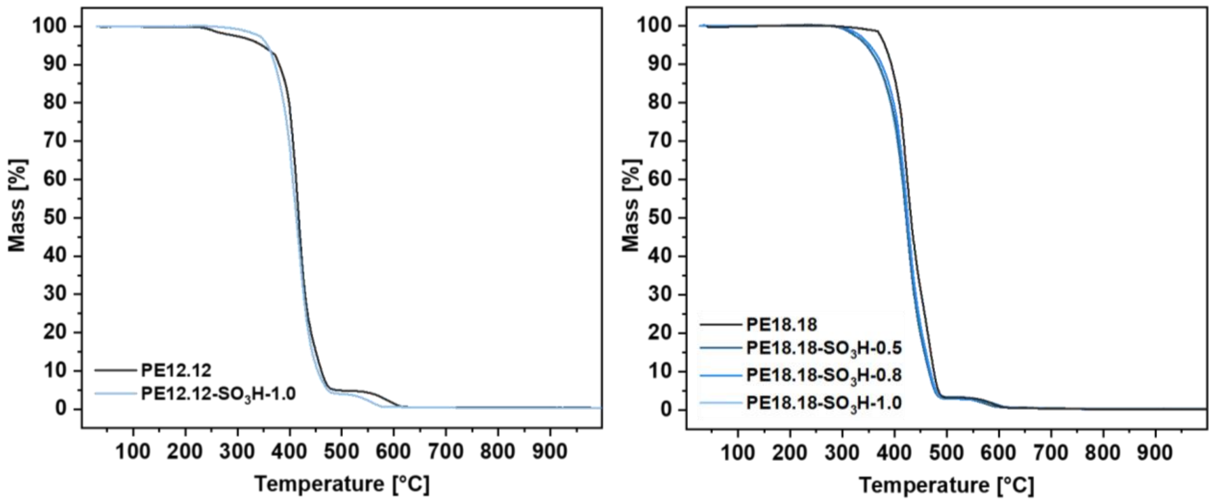

Figure S17 TGA traces of PE12.12 and PE12.12-SO_3H-1.0 (left) and PE18.18 and PE18.18-SO_3H (right) under air, measured from 30 to 1000 °C with a heating rate of 10 K/min.

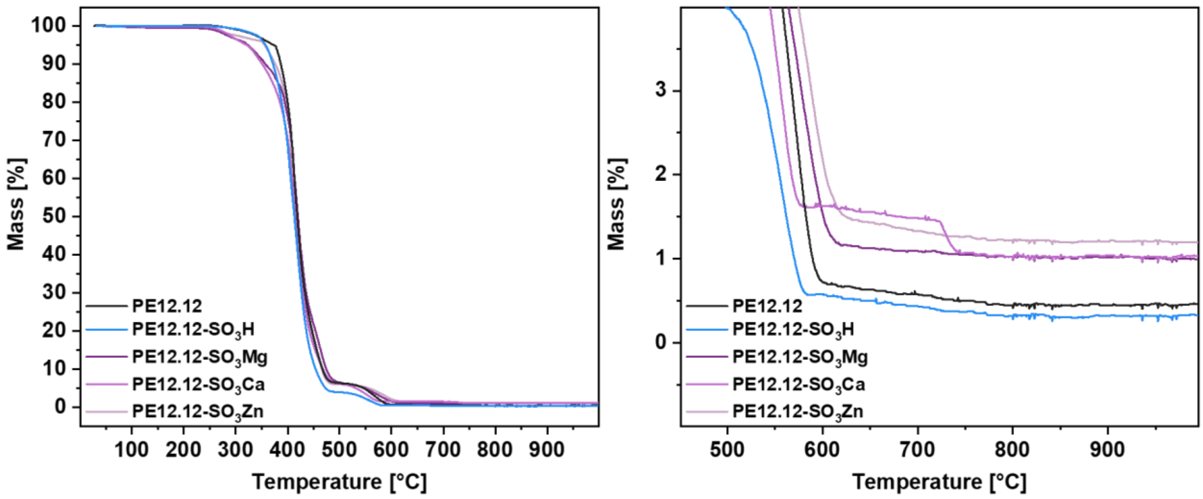

Figure S18 TGA traces of PE12.12, PE12.12-SO_3H-1.0 and PE12.12-SO_3M under air, measured from 30 to 1000 °C with a heating rate of 10 K/min. Left: full temperature range; right: detailed view on residual masses.

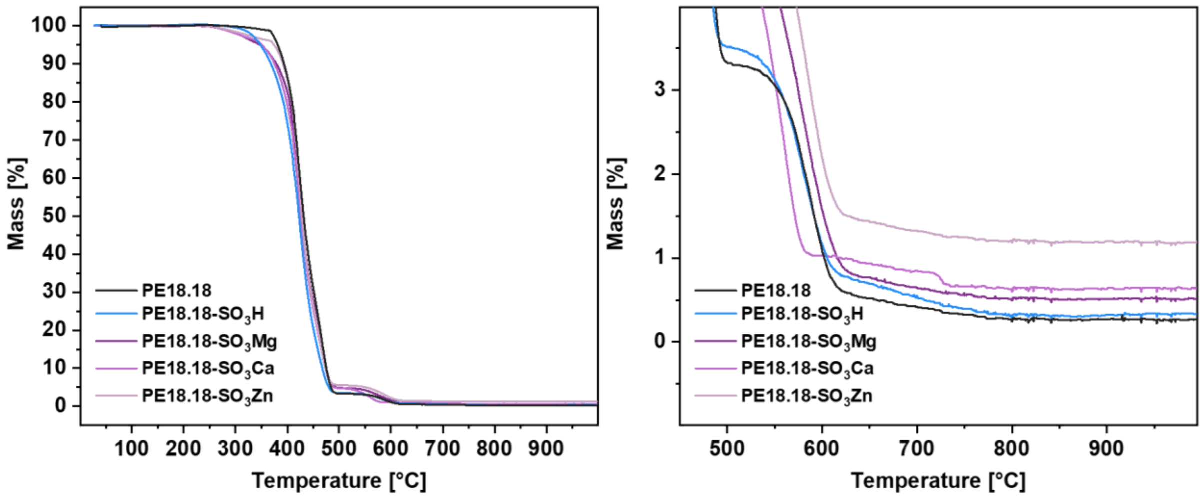

Figure S19 TGA traces of PE18.18, PE18.18-SO_3H-0.8 and PE18.18-SO_3M under air, measured from 30 to 1000 °C with a heating rate of 10 K/min. Left: full temperature range; right: detailed view on residual masses.

Table S9 Results from TGA (30 – 1000 °C, 10 K/min heating rate) of polyesters PE12.12-SO_3H-1.0 and PE18.18-SO_3H and their reference polyesters PE12.12 and PE18.18.

	T_{95} [°C] [a)]		T_{95} [°C] [a)]
**PE12.12**	374	**PE18.18**	381
		**PE18.18-SO_3H-0.5**	345
		**PE18.18-SO_3H-0.8**	352
**PE12.12-SO_3H-1.0**	358	**PE18.18-SO_3H-1.0**	348

a) Temperature at which a 5% weight loss was observed at 10 K/min.

Table S10 Results from TGA (30 – 1000 °C, 10 K/min heating rate) of polyesters PE12.12-SO_3M and PE18.18-SO_3M and their reference polyesters PE12.12 and PE18.18.

	**T_{95} [°C]** [a)]	**remaining weight [%]** [b)]
**PE12.12**	374	0.46
**PE12.12-SO_3Mg**	324	0.99
**PE12.12-SO_3Ca**	322	1.03
**PE12.12-SO_3Zn**	356	1.20
**PE18.18**	381	0.26
**PE18.18-SO_3Mg**	347	0.51
**PE18.18-SO_3Ca**	351	0.65
**PE18.18-SO_3Zn**	374	1.19

a) Temperature at which a 5% weight loss was observed at 10 K/min. b) Remaining weight at 1000 °C.

3.5. Tensile Testing Results

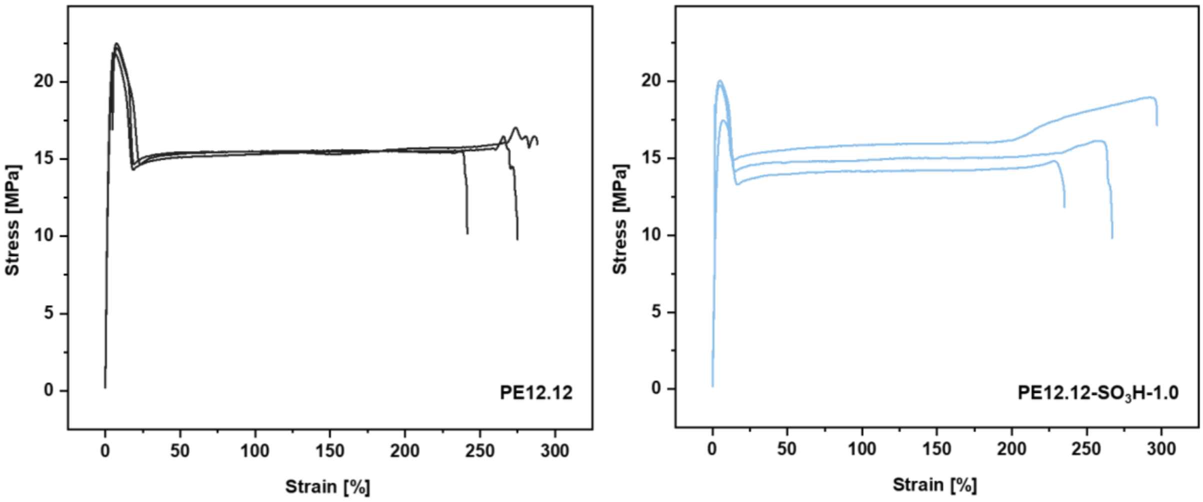

Figure S20 Stress-strain curves of PE12.12 and PE12.12-SO_3H-1.0. Three curves are shown each to demonstrate reproducibility.

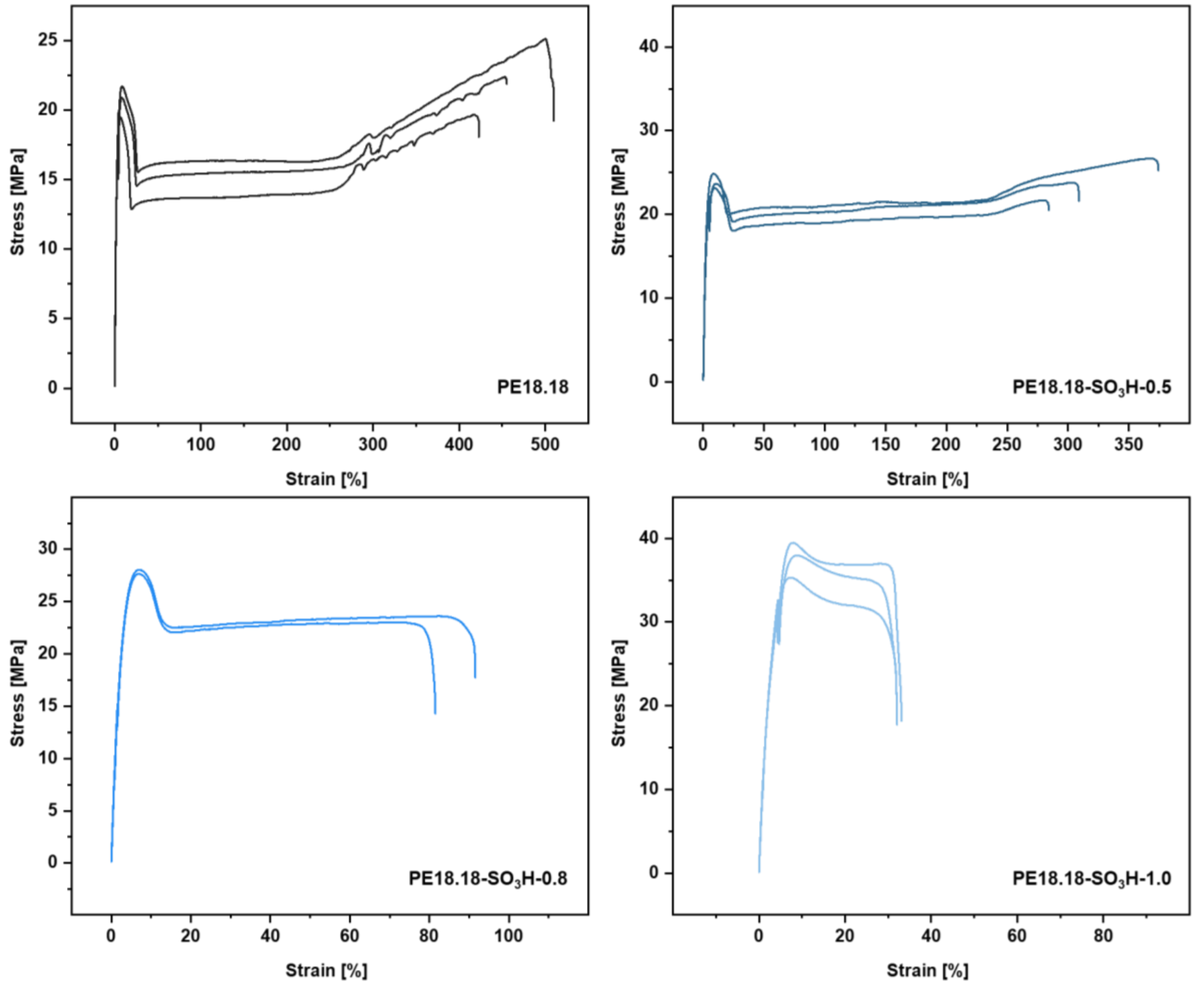

Figure S21 Stress-strain curves of PE18.18 and polymers PE18.18-SO_3H. Three curves are shown each (two curves in case of PE18.18-SO_3H-0.8) to demonstrate reproducibility.

Table S11 Tensile testing results of PE12.12-SO_3H and PE18.18-SO_3H.

	ε_{tb} a) [%]	U_t b) [J/m^3]	E_t c) [MPa]	σ_y d) [MPa]
**PE12.12**	270 ± 20	4300 ± 330	860 ± 30	22.2 ± 1.1
**PE12.12-SO_3H-1.0**	270 ± 25	4100 ± 640	595 ± 25	19.7 ± 1.0
**PE18.18**	490 ± 30	8700 ± 630	840 ± 10	21.3 ± 0.4
**PE18.18-SO_3H-0.5**	330 ± 40	6900 ± 1200	940 ± 30	23.9 ± 0.7
**PE18.18-SO_3H-0.8**	90 ± 5	2000 ± 140	1100 ± 35	27.8 ± 0.2
**PE18.18-SO_3H-1.0**	40 ± 3	1000 ± 40	1200 ± 40	35.4 ± 2.0

a) elongation at break, b) toughness, c) Young's modulus, d) stress at yield point.

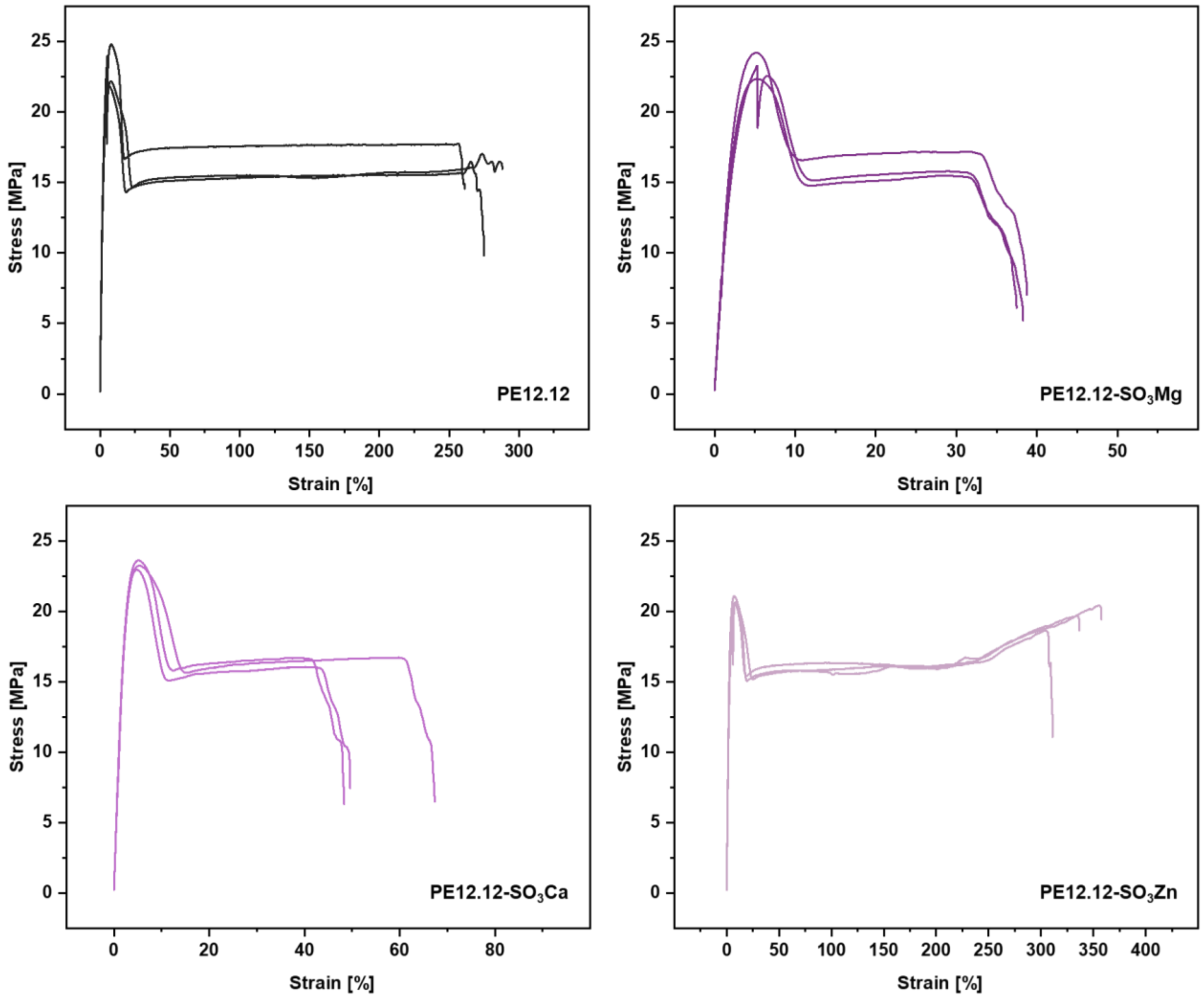

Figure S22 Tensile testing curves of PE12.12 and polymers PE12.12-SO_3M. Three curves are shown each to demonstrate reproducibility.

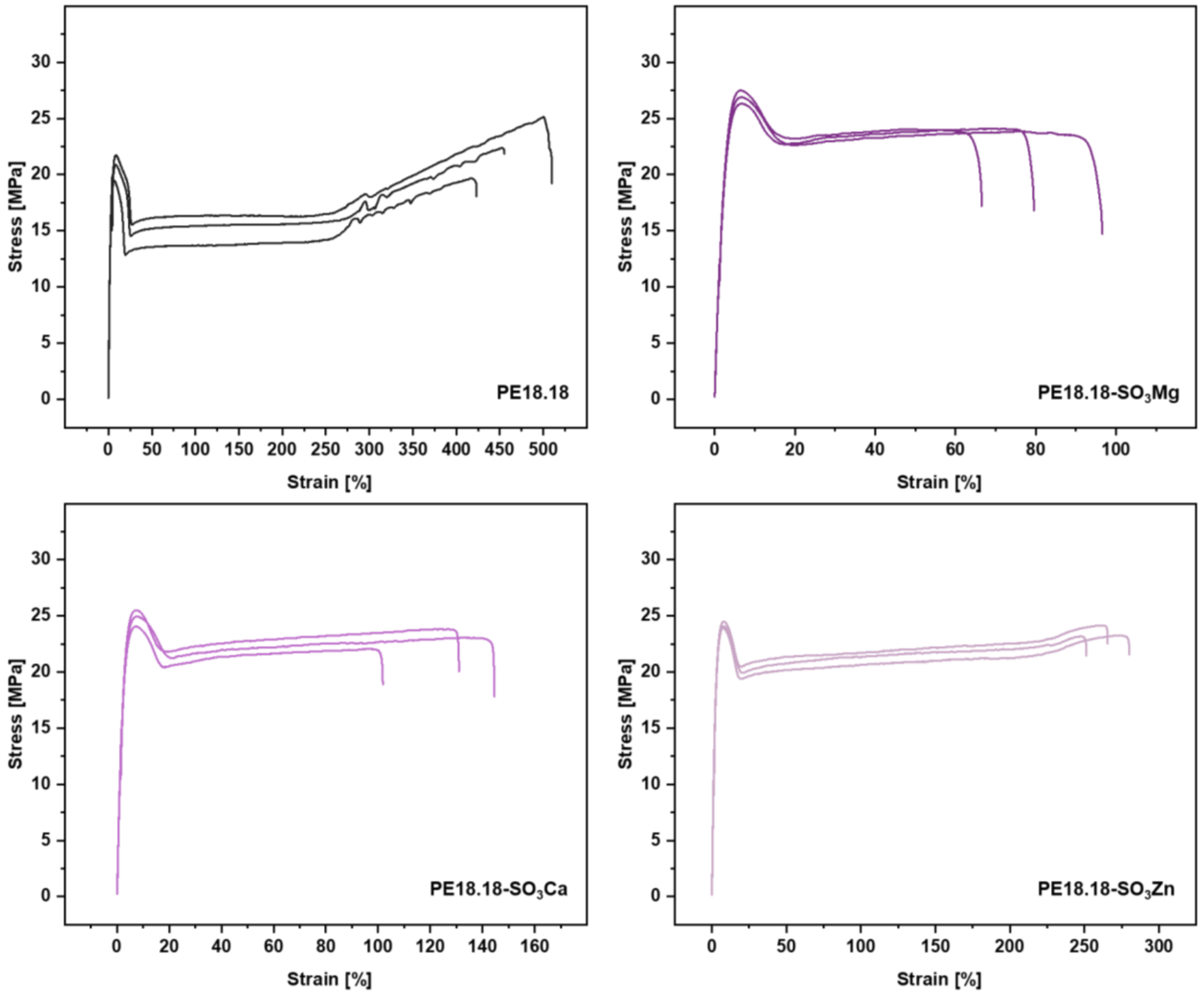

Figure S23 Tensile testing curves of PE18.18 and polymers PE18.18-SO_3M. Three curves are shown each to demonstrate reproducibility.

Table S12 Tensile testing results of PE12.12-SO_3M and PE18.18-SO_3M.

	ε_{tb} a) [%]	U_t b) [J/m^3]	E_t c) [MPa]	σ_y d) [MPa]
**PE12.12**	270 ± 20	4300 ± 330	860 ± 30	22.2 ± 1.1
**PE12.12-SO_3Mg**	40 ± 5	610 ± 30	900 ± 40	23.3 ± 0.8
**PE12.12-SO_3Ca**	60 ± 10	1000 ± 100	1020 ± 40	24.0 ± 0.9
**PE12.12-SO_3Zn**	340 ± 20	5600 ± 410	750 ± 20	20.6 ± 0.4
**PE18.18**	490 ± 30	8700 ± 630	840 ± 10	21.3 ± 0.4
**PE18.18-SO_3Mg**	80 ± 10	1900 ± 270	1010 ± 20	26.9 ± 0.5
**PE18.18-SO_3Ca**	120 ± 20	2800 ± 450	950 ± 50	24.8 ± 0.6
**PE18.18-SO_3Zn**	270 ± 10	5700 ± 230	930 ± 20	26.8 ± 0.2

a) elongation at break, b) toughness, c) Young's modulus, d) stress at yield point.

3.6. Water Contact Angle Measurements

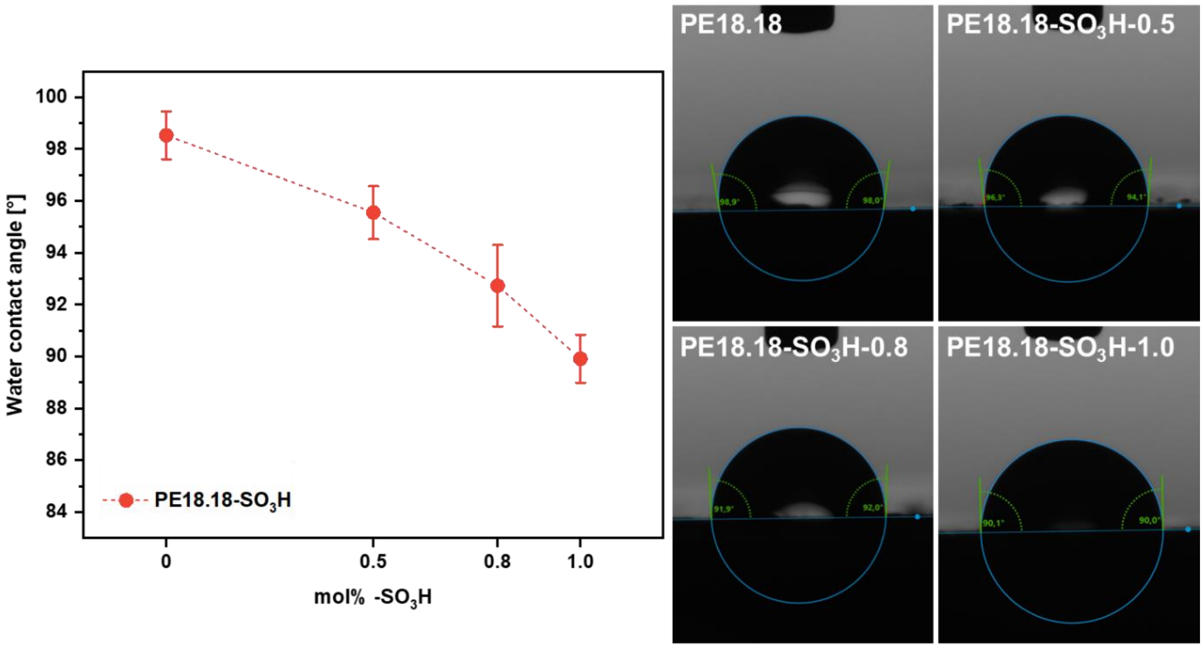

Figure S24 Water contact angle of PE18.18-SO_3H as well as the non-ion containing reference polyester PE18.18. Error bars represent standard deviations calculated from three different droplets (six angles). Samples were surface cleaned with ^{i}PrOH and dried for 48 h prior to the measurements. Images show representative measurements of each polymer from the series PE18.18-SO_3H.

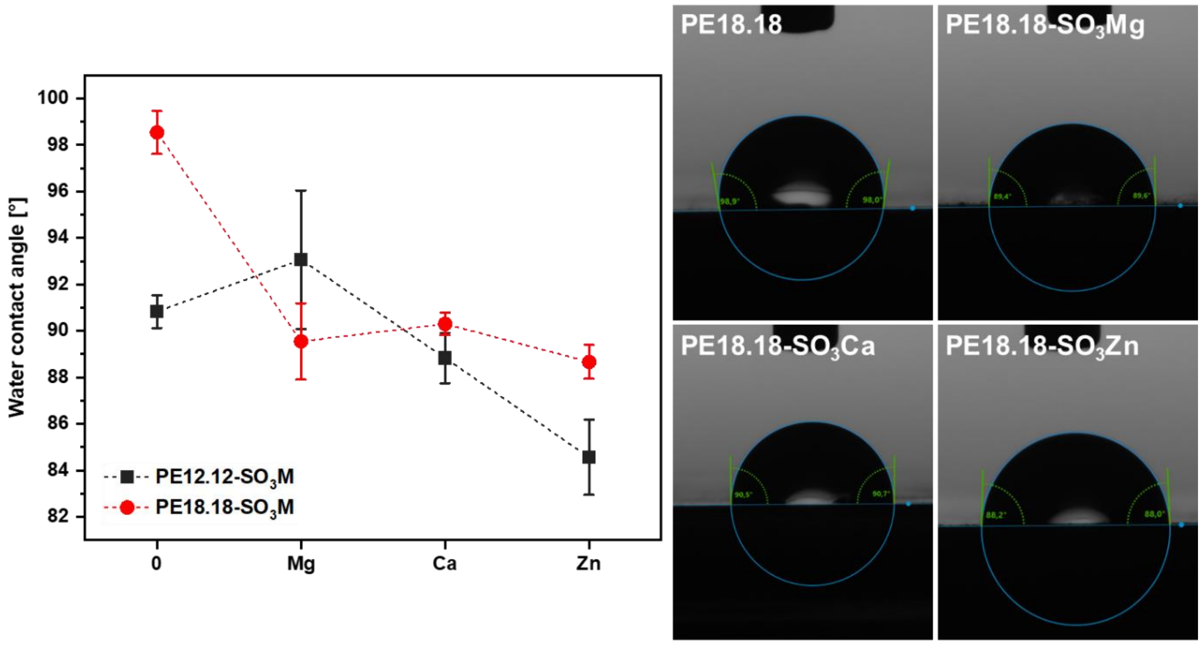

Figure S25 Water contact angle of PE12.12-SO_3M and PE18.18-SO_3M as well as their non-ion containing reference polyesters, PE12.12 and PE18.18. Error bars represent standard deviations calculated from three different droplets (six angles). Samples were surface cleaned with iPrOH and dried for 48 h prior to the measurements. Images show representative measurements of each polymer from the series PE18.18-SO_3M.

3.7. Ink Adsorption on Film Surfaces

The results before and after wiping off the color on all PE18.18-SO_3M films are depicted in Figure S26.

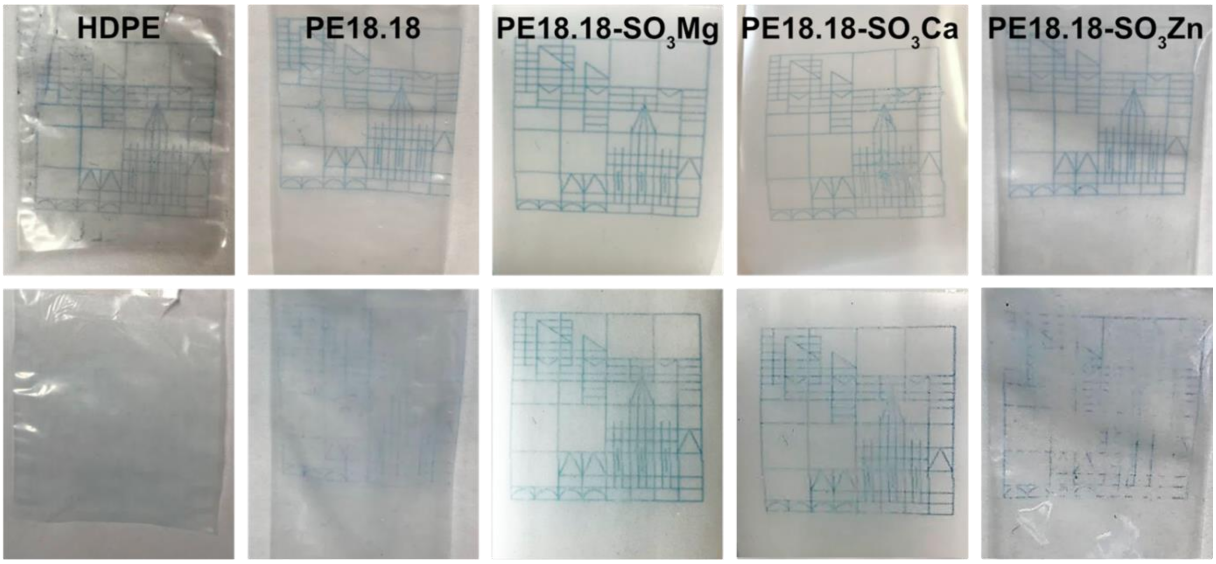

Figure S26 Photographs of imprinted films of HDPE, PE18.18 and PE18.18-SO_3M. before (top row) and after (bottom row) wiping off the color.

To test whether the observed effect originates from the excess stearate or from the incorporated ionic groups, PE12.12, PE12.12-SO_3Mg and PE12.12-$Mg_{stearate}$ were compared (Figure S27), as well as PE12.12, PE12.12-SO_3Ca and PE12.12-$Ca_{stearate}$ (Figure S28). A significant blurring effect in case of PE12.12 is visible, yet the PE12.12-SO_3Mg shows a high persistency of the ink. However, the reference polyester PE12.12-$Mg_{stearate}$, which also contains excess stearate but has no ionic groups incorporated in the polymer backbone, shows a significant blurring effect, comparable to that seen in PE12.12. Therefore, the improved adhesion capability of the investigated polyesters cannot be concluded from the incorporated stearate, but mainly derives from the incorporated ionic groups.

Note that film drawing was possible with all polymers PE12.12-SO_3M and PE18.18-SO_3M, as well as with the reference polymers PE12.12, PE18.18 and HDPE. For PE12.12-$M_{stearate}$, films were only possible to draw for PE12.12-$Mg_{stearate}$ and PE12.12-$Ca_{stearate}$. The melt viscosity of PE12.12-$Zn_{stearate}$ was too low and no continuous film could be drawn. Consequently, only magnesium and calcium containing polymers are compared.

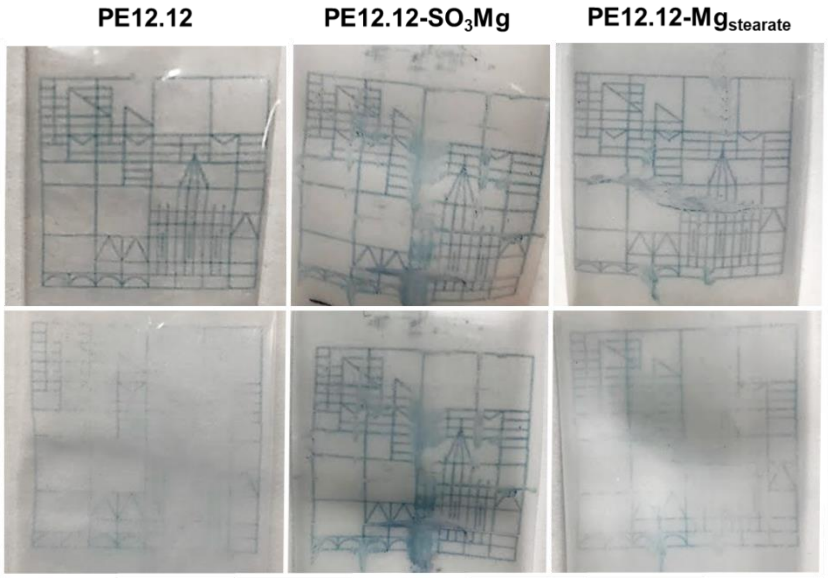

Figure S27 Photographs of imprinted films of PE12.12, PE12.12-SO_3Mg and PE12.12-$Mg_{stearate}$ before (top row) and after (bottom row) wiping.

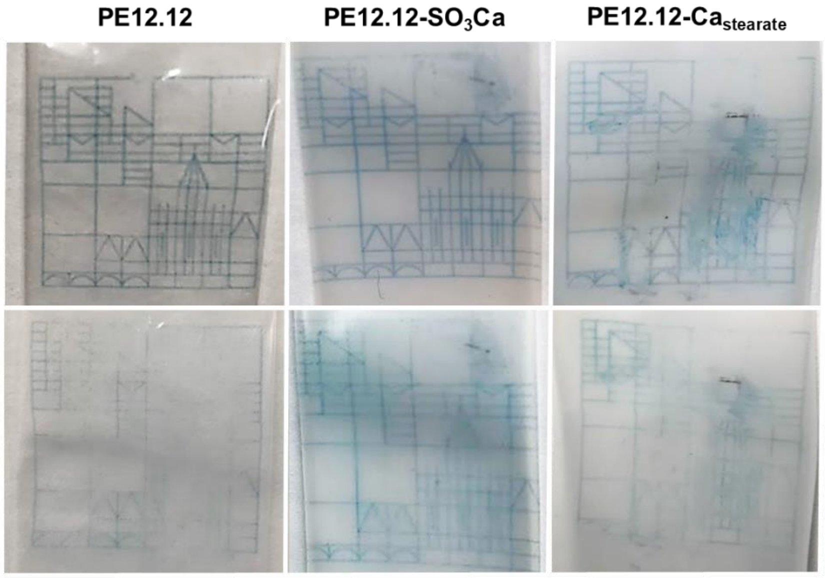

Figure S28 Photographs of imprinted films of PE12.12, PE12.12-SO_3Ca and PE12.12-$Ca_{stearate}$ before (top row) and after (bottom row) wiping.

3.8. Water Uptake Study

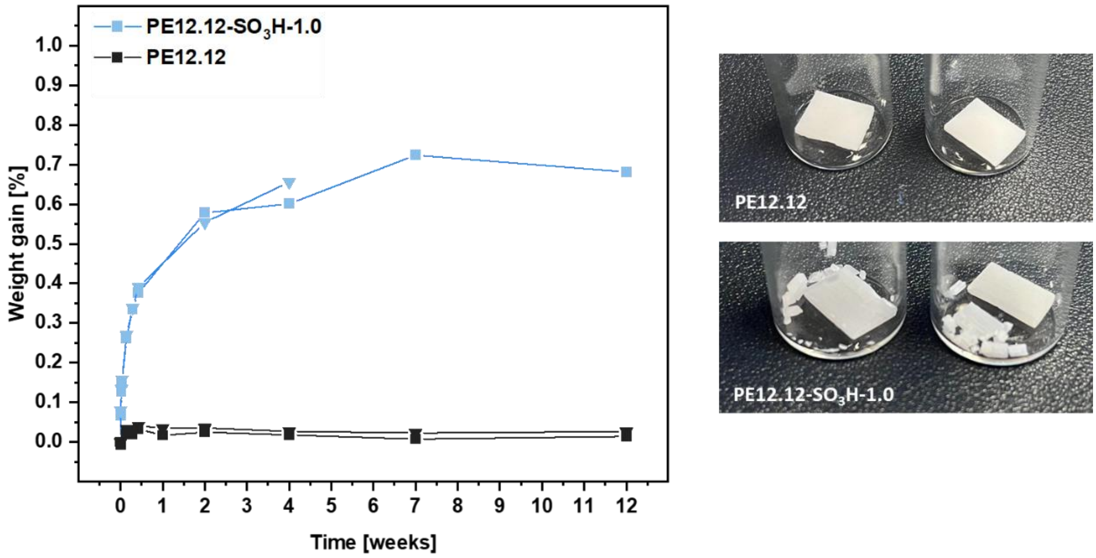

Figure S29 Left: Weight gain of PE12.12 and PE12.12-SO$_3$H-1.0 after storage in water for 12 weeks. Duplicates were investigated. In case of PE12.12-SO$_3$H-1.0, one of the samples embrittled significantly after 4 weeks and could not further be weighed but was incubated further. Right: Images of dried samples after 12 weeks.

Table S13 Degrees of polymerization as determined from ^{1}H NMR spectra of samples in the water uptake study of PE12.12, PE12.12-SO$_3$H-1.0 and PE18.18, PE18.18-SO$_3$H. Values before the study and after 10 and 12 weeks, respectively, of exposure to water are listed.

	DP_n 0 weeks	DP_n 12 weeks		DP_n 0 weeks	DP_n 10 weeks
**PE12.12**	98	101	**PE18.18**	68	64
			**PE18.18-SO$_3$H-0.5**	74	39
			**PE18.18-SO$_3$H-0.8**	94	33
**PE12.12-SO$_3$H-1.0**	70	28	**PE18.18-SO$_3$H-1.0**	118	38

3.9. ^{1}H NMR Spectra of Polymers PE12.12-SO$_3$H and PE18.18-SO$_3$H

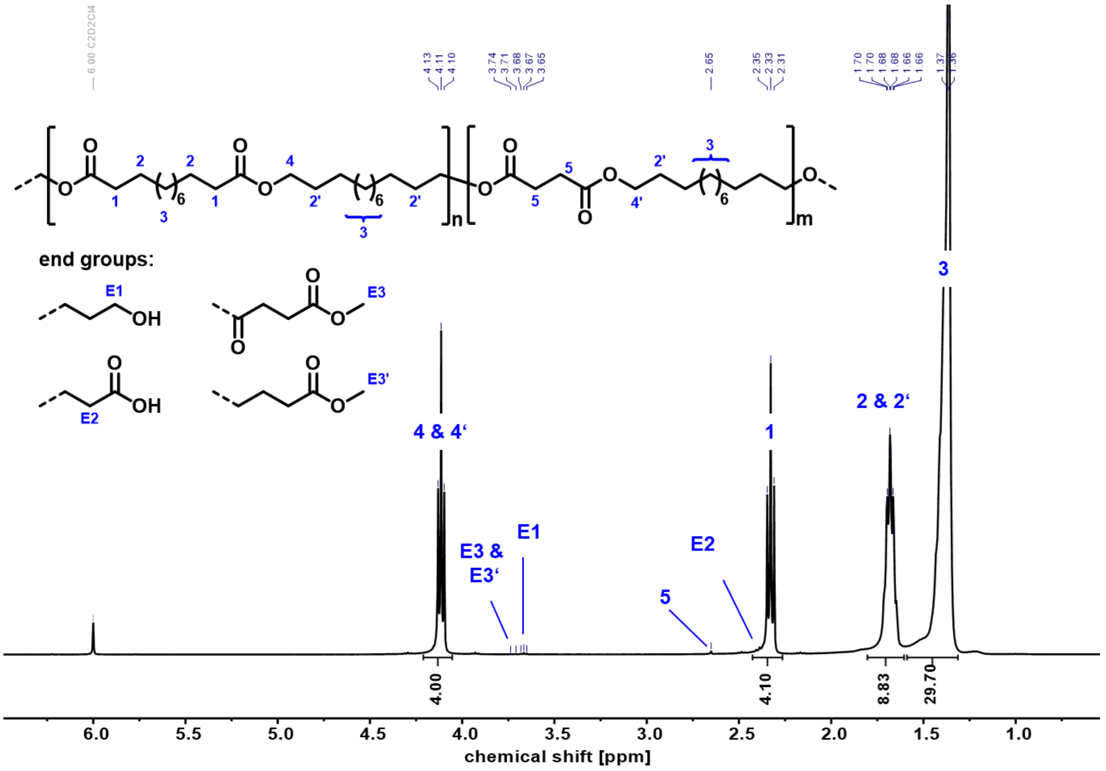

Figure S30 Full ^{1}H NMR spectrum (400 MHz, 383 K, $C_2D_2Cl_4$) of **PE12.12** containing 1.0 mol% of unfunctionalized succinate as reference polyester.

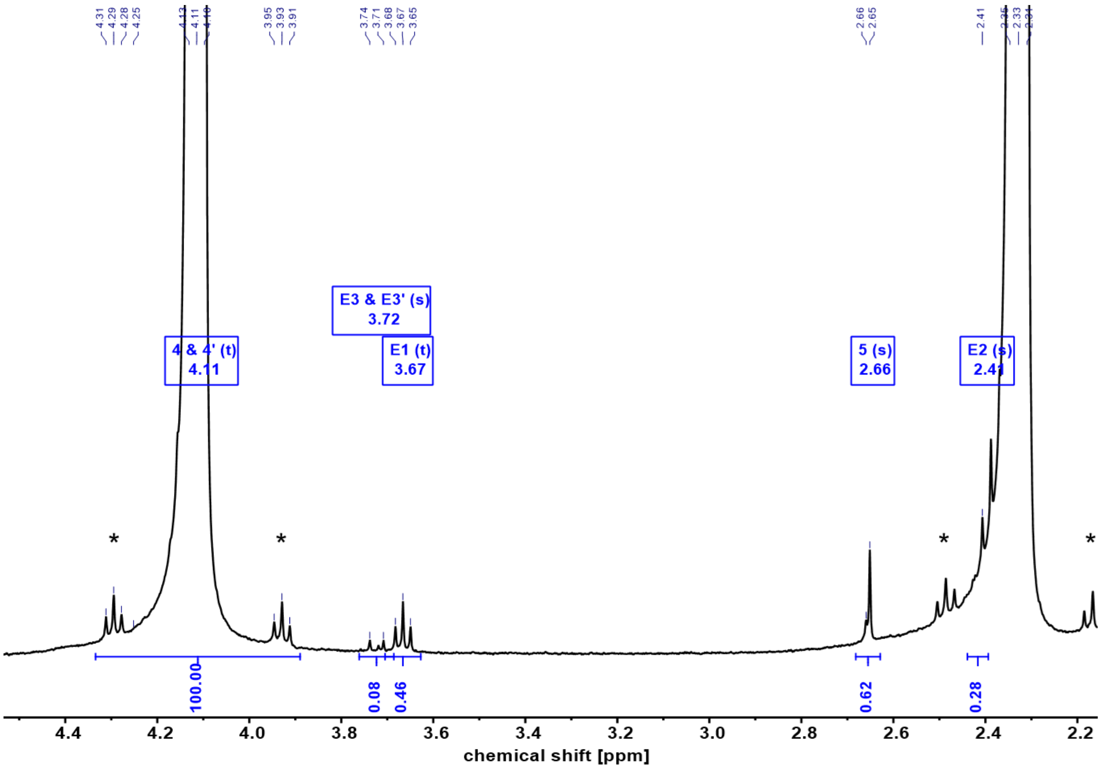

Figure S31 Detailed section of the ^{1}H NMR spectrum (400 MHz, 383 K, $C_2D_2Cl_4$) of **PE12.12** with integrals of end groups (* = ^{13}C-coupled satellites) and of the succinate unit. Assignment of signals according to **Figure S30**.

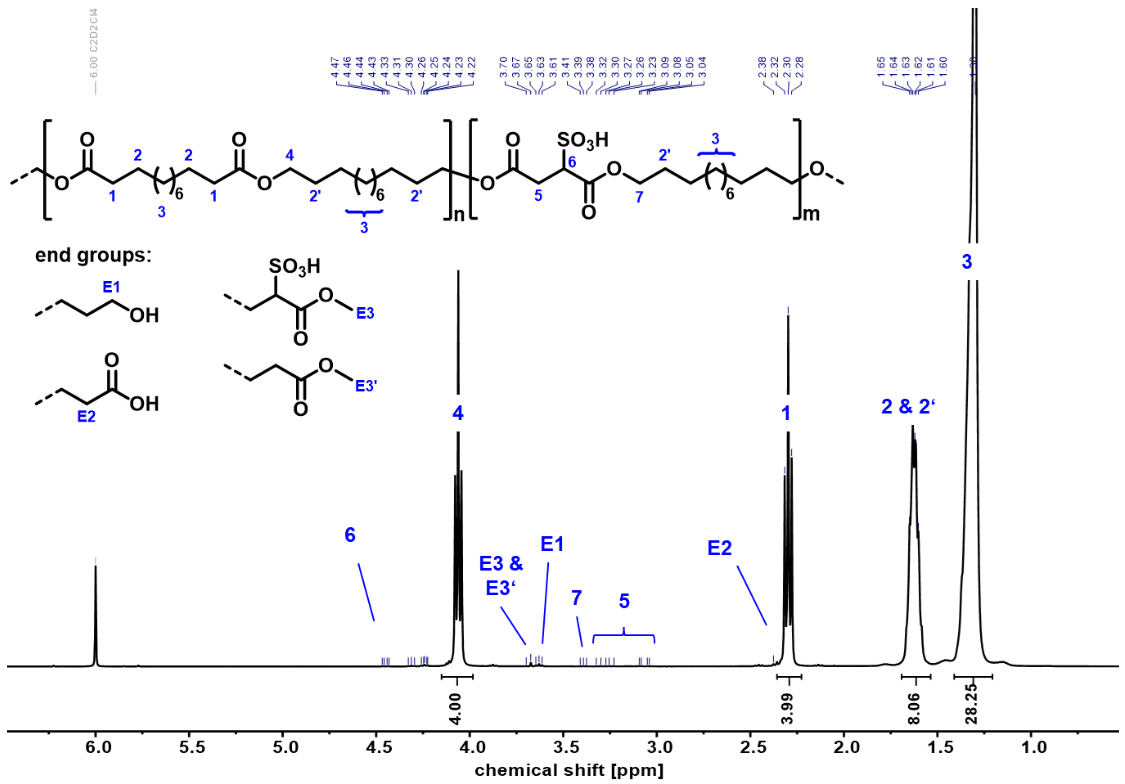

Figure S32 ^{1}H NMR spectrum (400 MHz, 323 K, $C_2D_2Cl_4$) of **PE12.12-SO$_3$H-1.0**. Note that protons 5 located next to the -SO_3H group are diastereotopic and that the -SO_3H group can be oriented either as shown, or the repeat unit can be oppositely arranged.

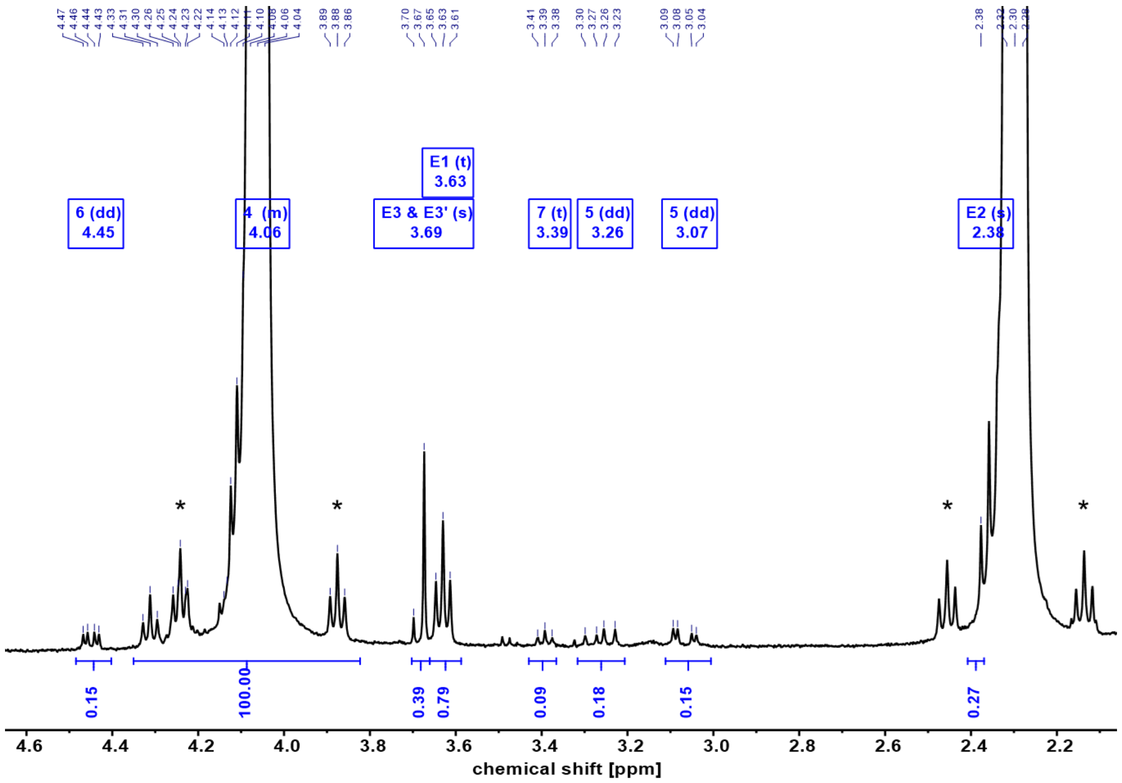

Figure S33 Detailed of the ^{1}H NMR spectrum (400 MHz, 323 K, $C_2D_2Cl_4$) of **PE12.12-SO$_3$H-1.0** with integrals of end groups as well as protons located close to the -SO_3H group (* = ^{13}C-coupled satellites). Assignment of signals according to **Figure S32**.

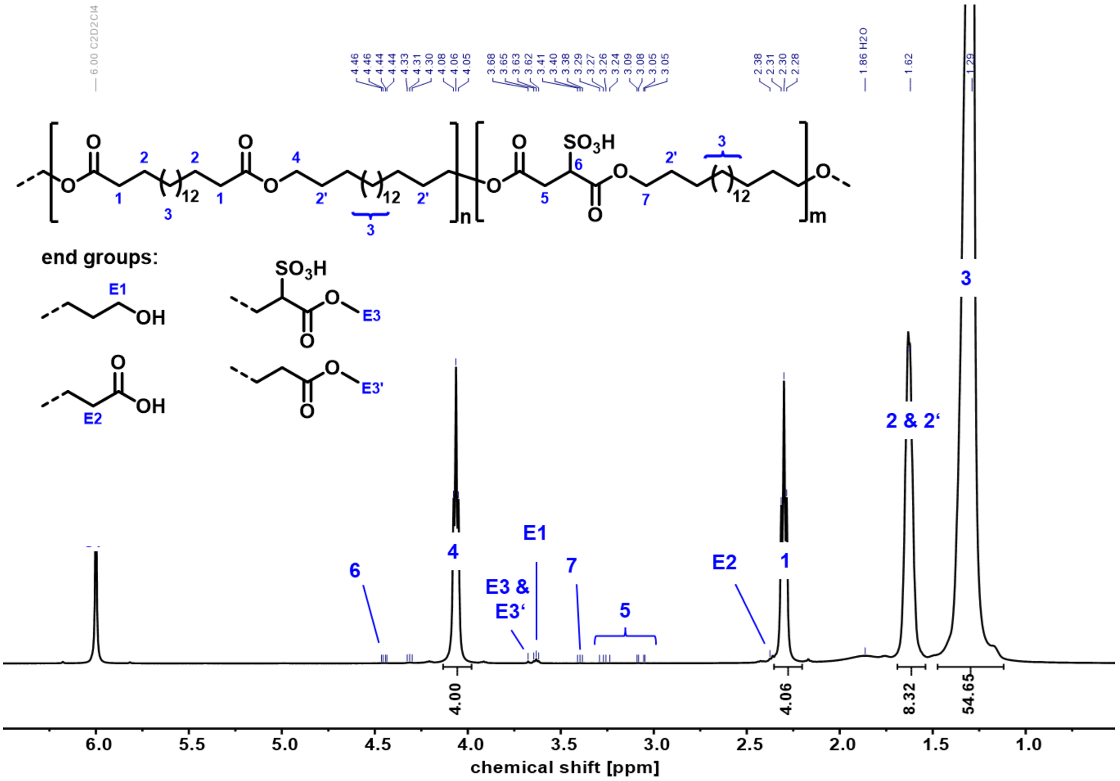

Figure S34 ^{1}H NMR spectrum (500 MHz, 323 K, $C_2D_2Cl_4$) of **PE18.18-SO$_3$H-0.8**. Note that protons 5 located next to the -SO_3H group are diastereotopic and that the -SO_3H group can be oriented either as shown, or the repeat unit can be oppositely arranged.

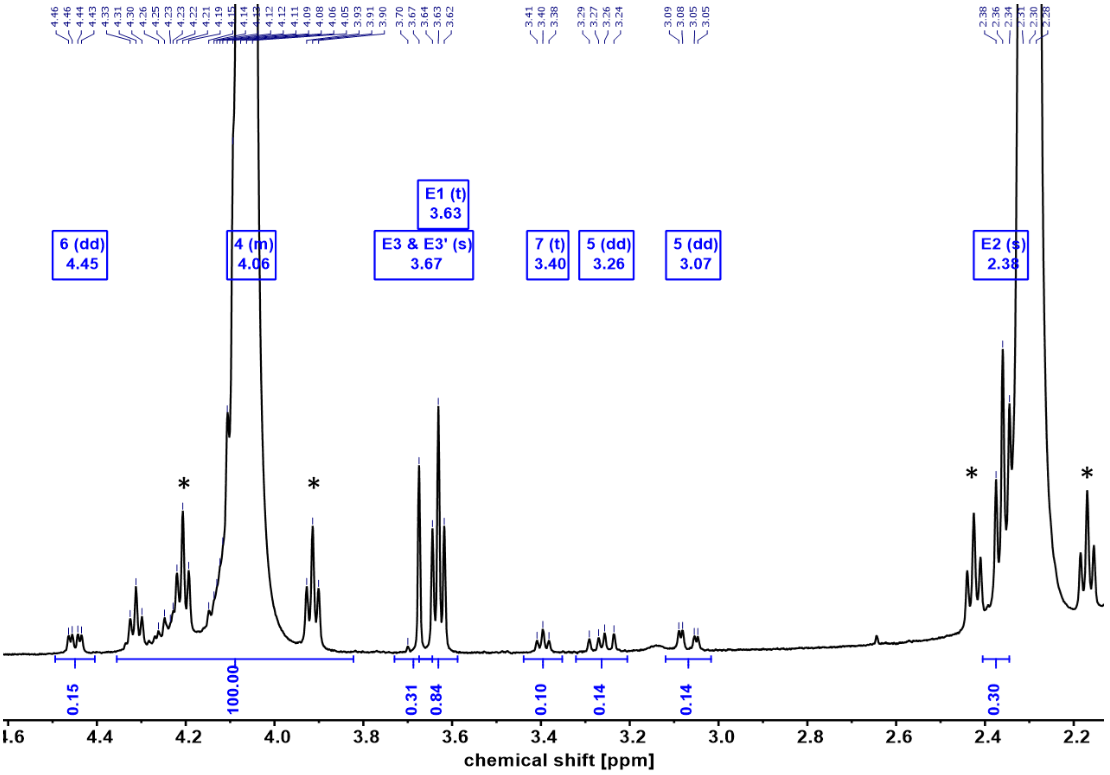

Figure S35 Detail of the ^{1}H NMR spectrum (500 MHz, 323 K, $C_2D_2Cl_4$) of **PE18.18-SO$_3$H-0.8** with integrals of end groups as well as protons located close to the -SO_3H group (* = ^{13}C-coupled satellites). Assignment of signals according to **Figure S34**.

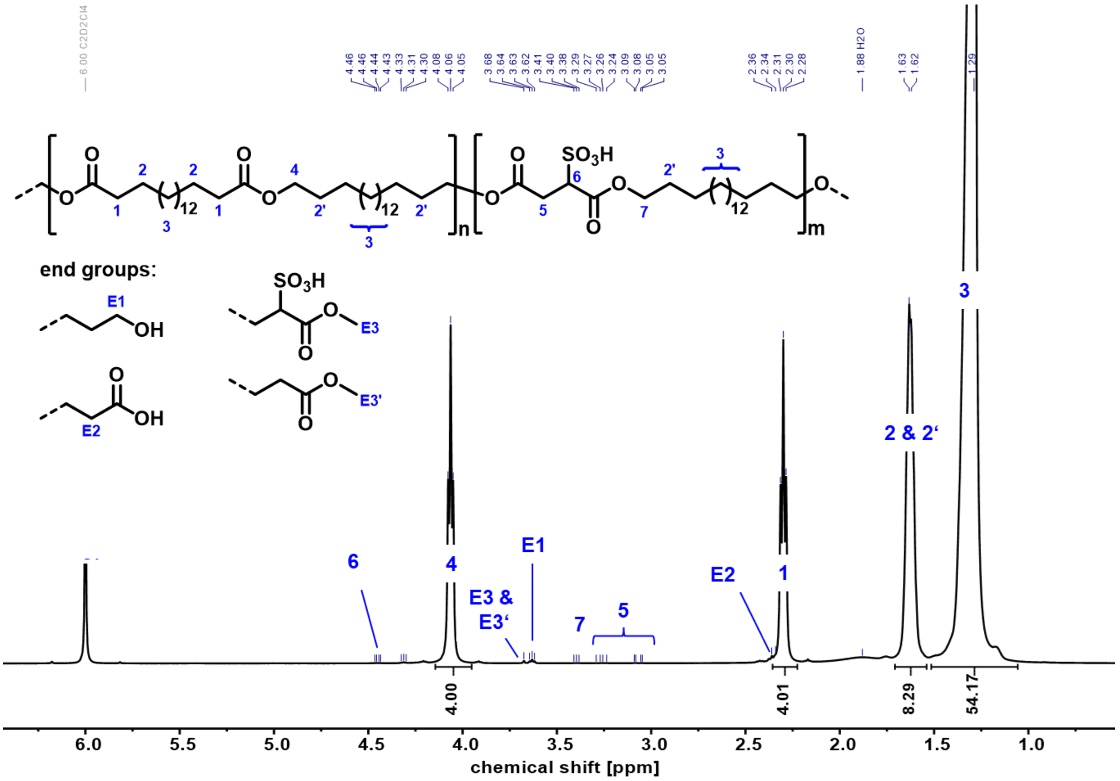

Figure S36 ^{1}H NMR spectrum (500 MHz, 323 K, $C_2D_2Cl_4$) of **PE18.18-SO3H-1.0**. Note that protons 5 located next to the -SO_3H group are diastereotopic and that the -SO_3H group can be oriented either as shown, or the repeat unit can be oppositely arranged.

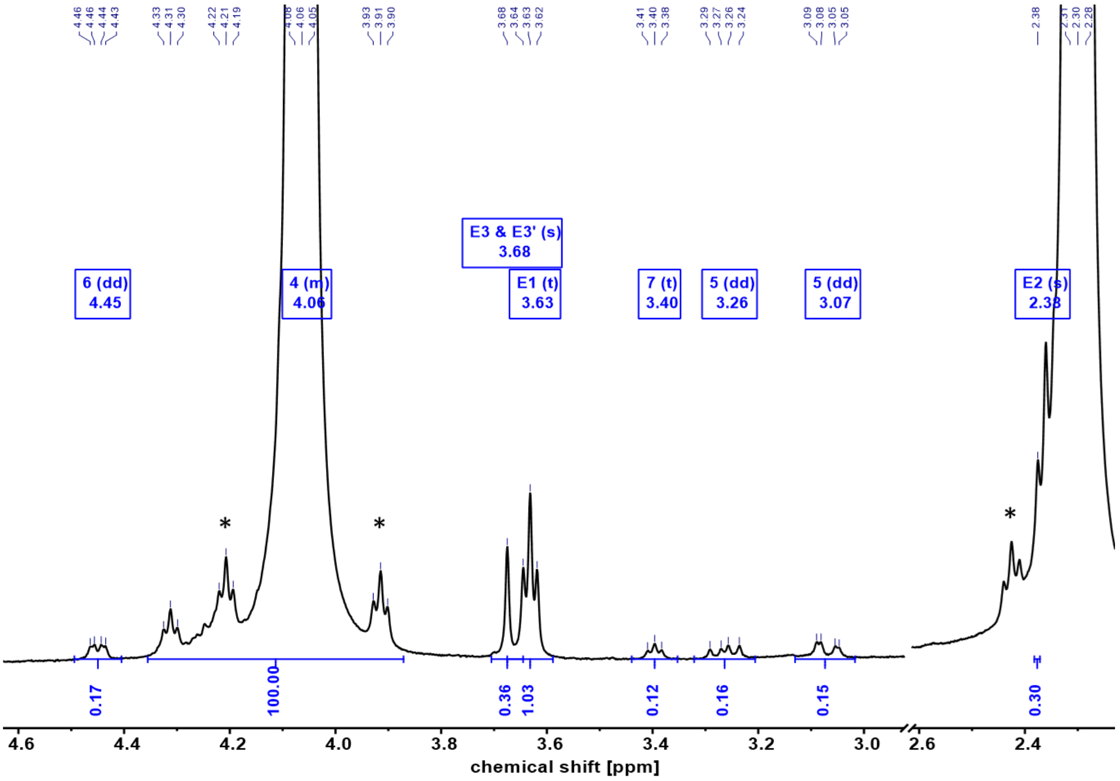

Figure S37 Detail of the ^{1}H NMR spectrum (500 MHz, 323 K, $C_2D_2Cl_4$) of **PE18.18-SO3H-1.0** with integrals of end groups as well as protons located close to the -SO_3H group (* = ^{13}C-coupled satellites). Assignment of signals according to **Figure S36**.

3.10. ^{1}H NMR Spectra of Polymers PE12.12-SO3M and PE18.18-SO3M

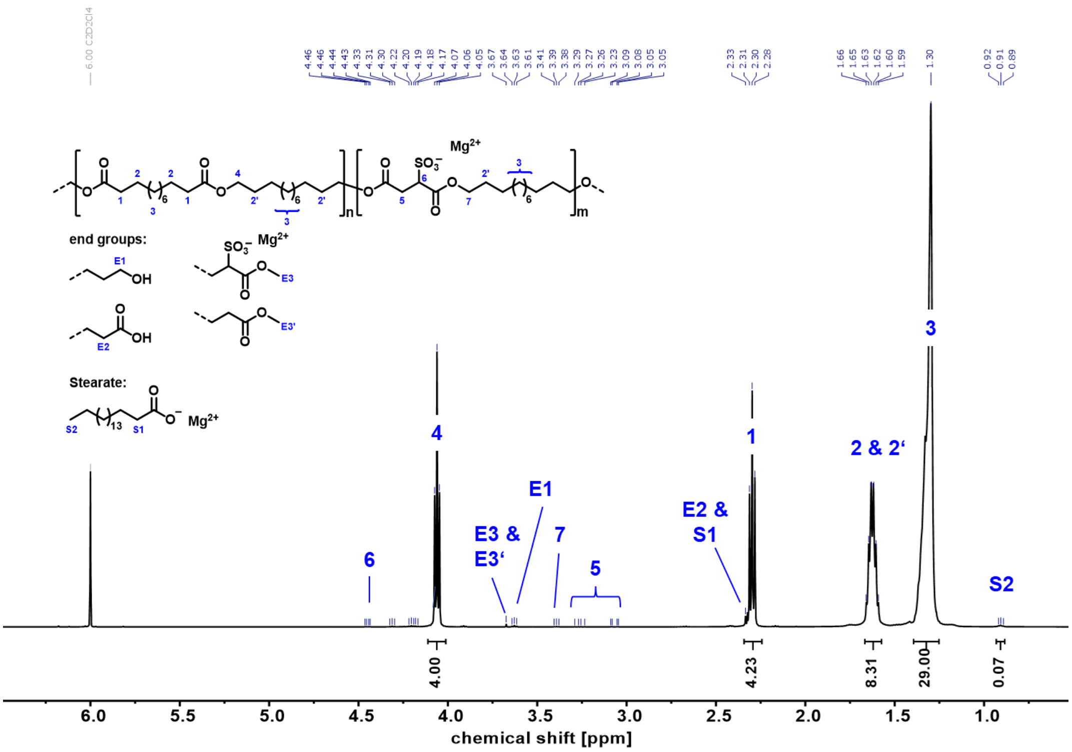

Figure S38 ^{1}H NMR spectrum (500 MHz, 323 K, $C_2D_2Cl_4$) of **PE12.12-SO3Mg**. Note that protons 5 located next to the sulfonate group are diastereotopic and that the sulfonate group can be oriented either as shown, or the repeat unit can be oppositely arranged.

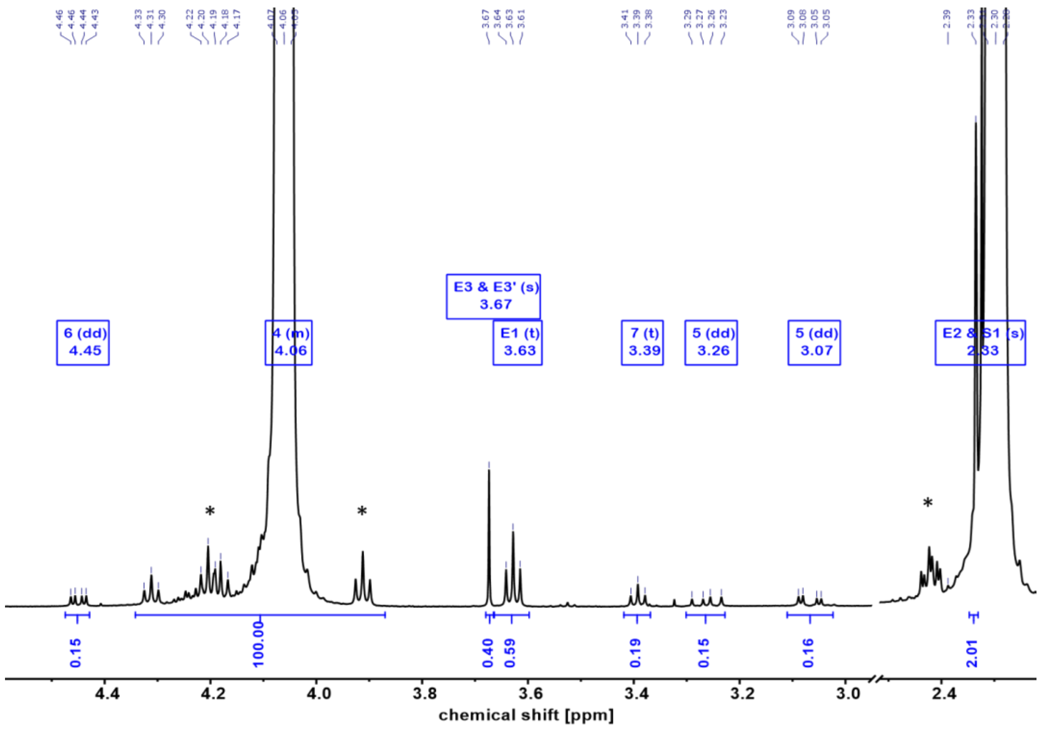

Figure S39 Detail of the ^{1}H NMR spectrum (500 MHz, 323 K, $C_2D_2Cl_4$) of **PE12.12-SO3Mg** with integrals of end groups as well as protons located close to the sulfonate group (* = ^{13}C-coupled satellites). Assignment of signals according to **Figure S38.**

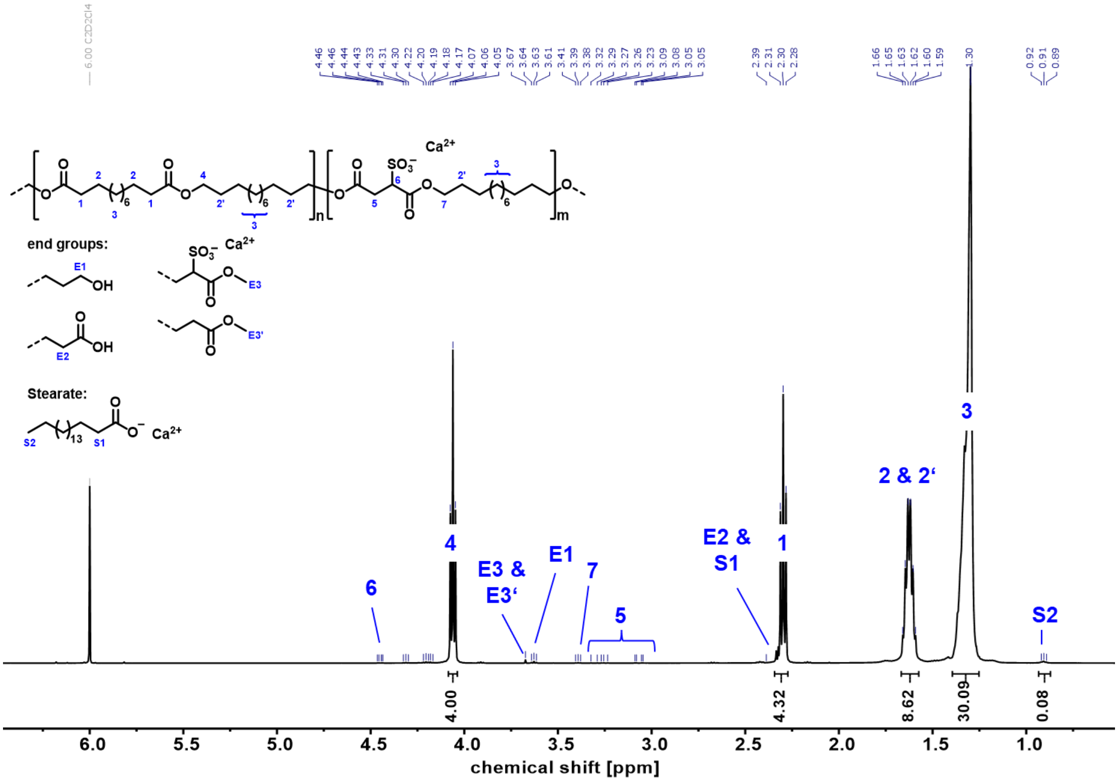

Figure S40 ^{1}H NMR spectrum (500 MHz, 323 K, $C_2D_2Cl_4$) of **PE12.12-SO$_3$Ca.** Note that protons 5 located next to the sulfonate group are diastereotopic and that the sulfonate group can be oriented either as shown, or the repeat unit can be oppositely arranged.

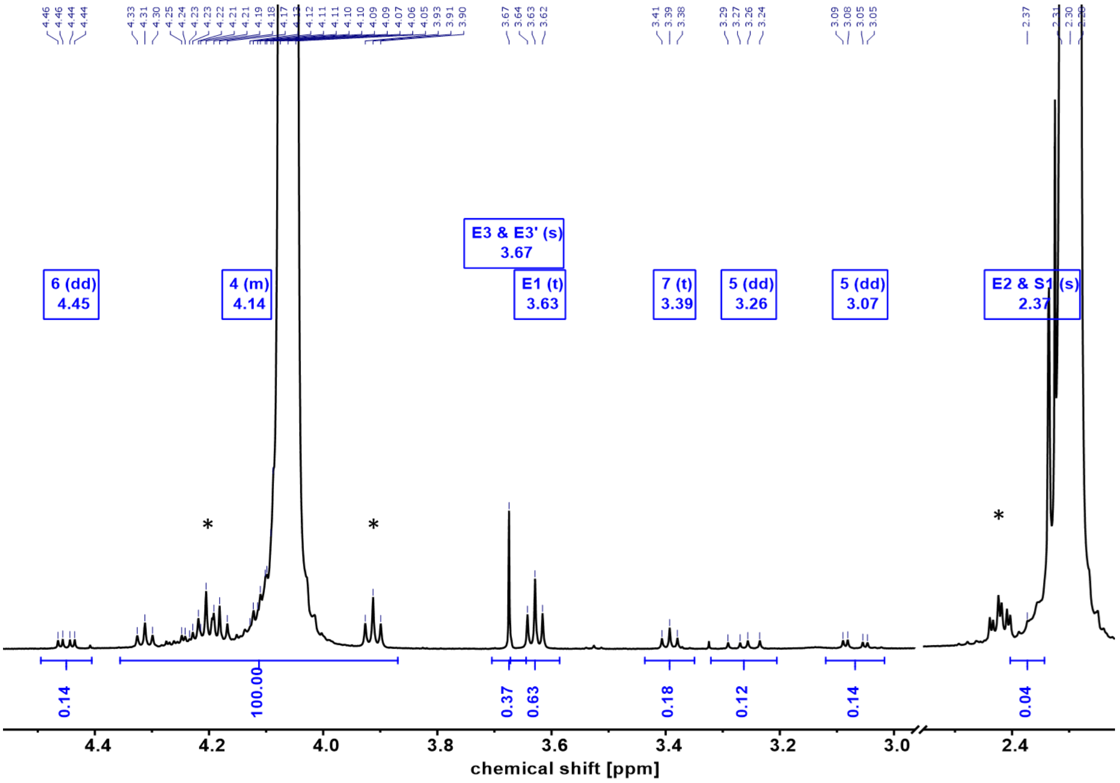

Figure S41 Detail of the ^{1}H NMR spectrum (500 MHz, 323 K, $C_2D_2Cl_4$) of **PE12.12-SO$_3$Ca** with integrals of end groups as well as protons located close to the sulfonate group (* = ^{13}C-coupled satellites). Assignment of signals according to **Figure S40.**

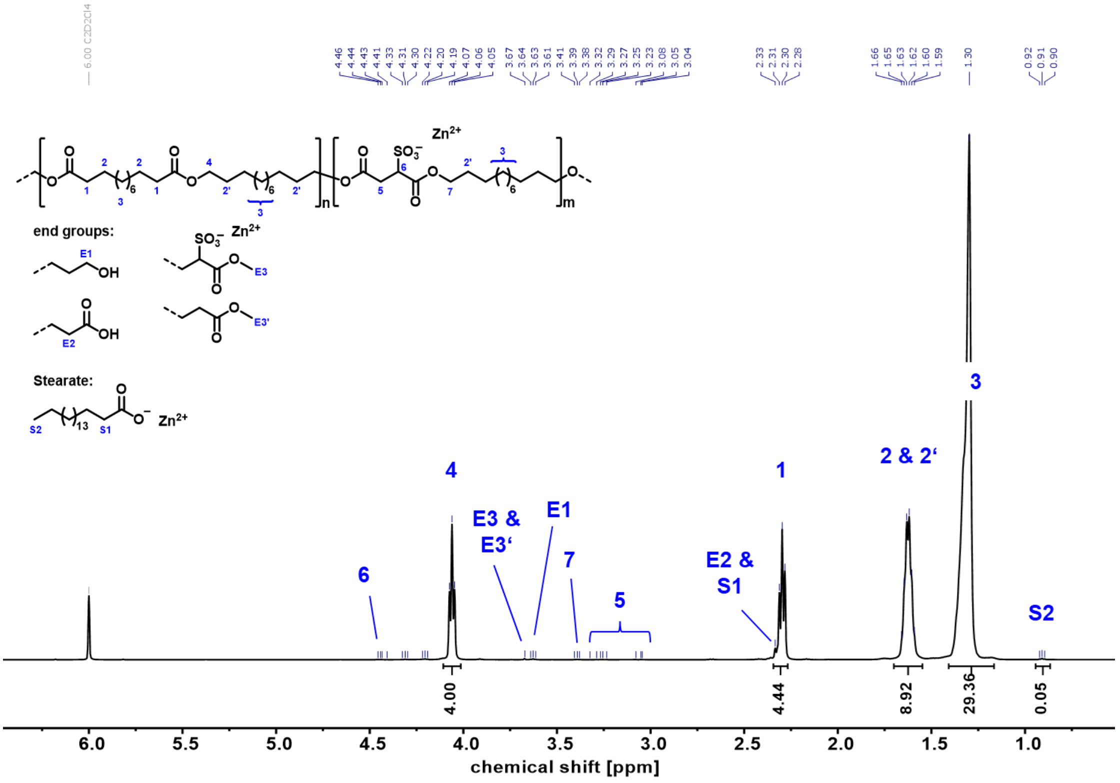

Figure S42 ^{1}H NMR spectrum (500 MHz, 323 K, $C_2D_2Cl_4$) of **PE12.12-SO$_3$Zn.** Note that protons 5 located next to the sulfonate group are diastereotopic and that the sulfonate group can be oriented either as shown, or the repeat unit can be oppositely arranged.

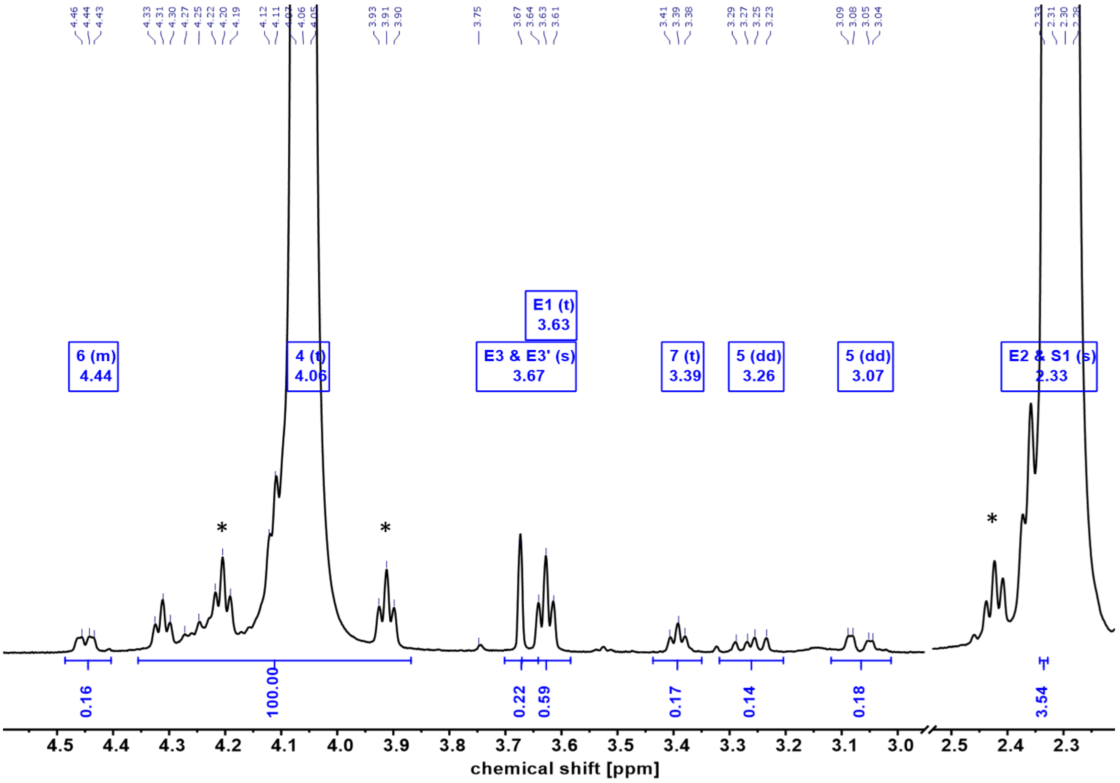

Figure S43 Detail of the ^{1}H NMR spectrum (500 MHz, 323 K, $C_2D_2Cl_4$) of **PE12.12-SO$_3$Zn** with integrals of end groups as well as protons located close to the sulfonate group (* = ^{13}C-coupled satellites). Assignment of signals according to **Figure S42**.

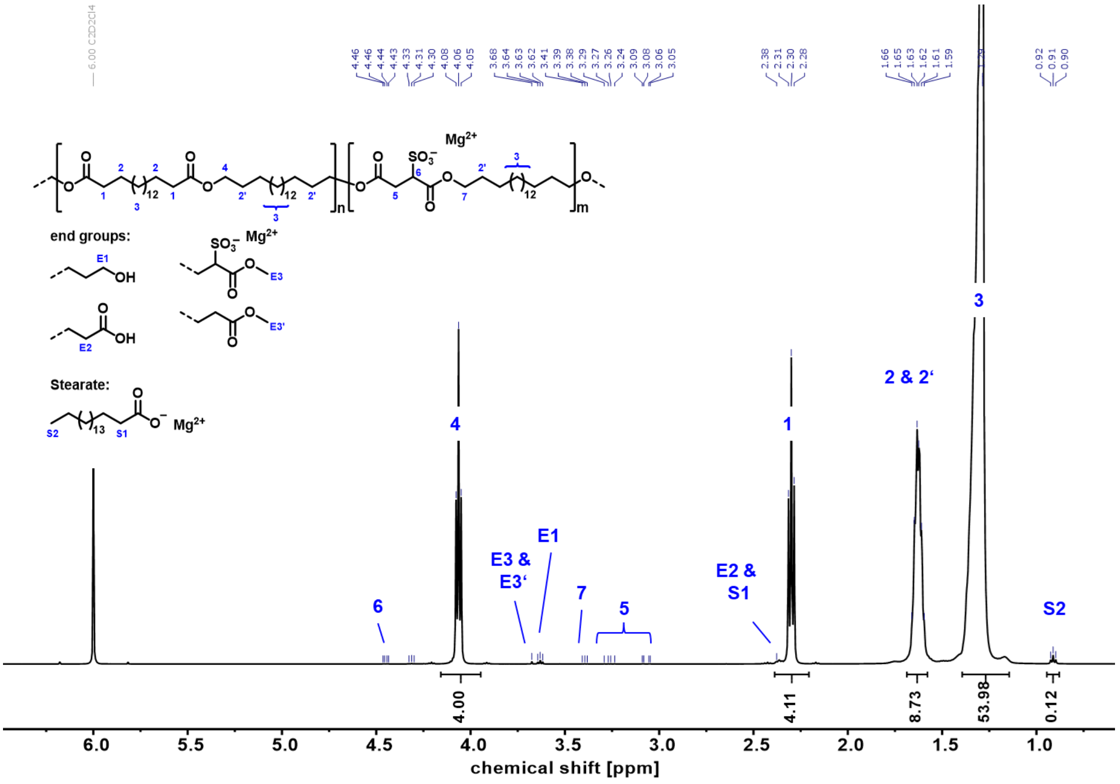

Figure S44 ^{1}H NMR spectrum (500 MHz, 323 K, $C_2D_2Cl_4$) of **PE18.18-SO$_3$Mg**. Note that protons 5 located next to the sulfonate group are diastereotopic and that the sulfonate group can be oriented either as shown, or the repeat unit can be oppositely arranged.

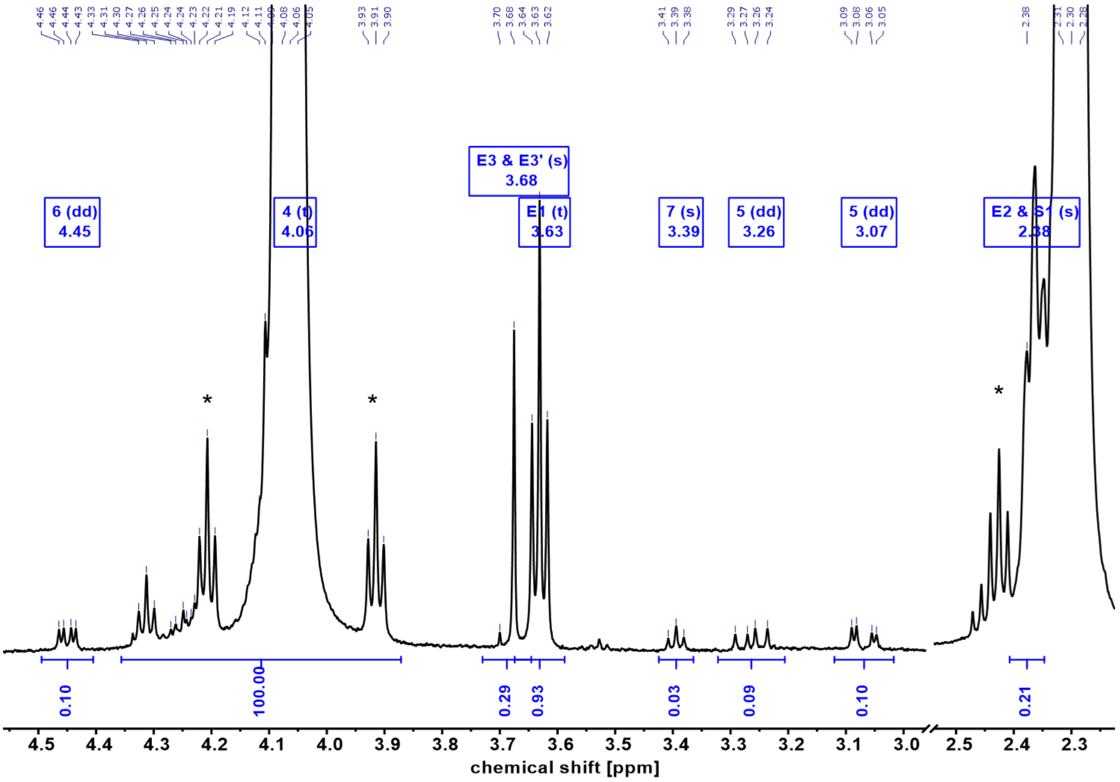

Figure S45 Detail of the ^{1}H NMR spectrum (500 MHz, 323 K, $C_2D_2Cl_4$) of **PE18.18-SO$_3$Mg** with integrals of end groups as well as protons located close to the sulfonate group (* = ^{13}C-coupled satellites). Assignment of signals according to **Figure S44**.

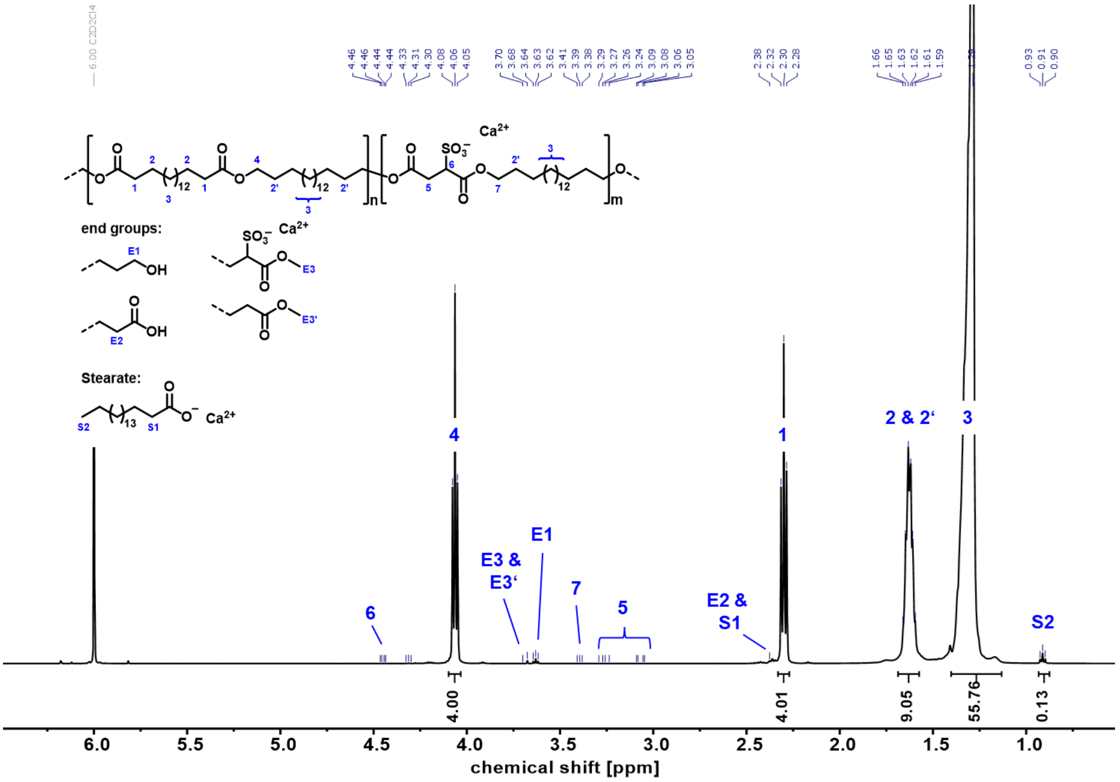

Figure S46 ^{1}H NMR spectrum (500 MHz, 323 K, $C_2D_2Cl_4$) of **PE18.18-SO_3Ca**. Note that protons 5 located next to the sulfonate group are diastereotopic and that the sulfonate group can be oriented either as shown, or the repeat unit can be oppositely arranged.

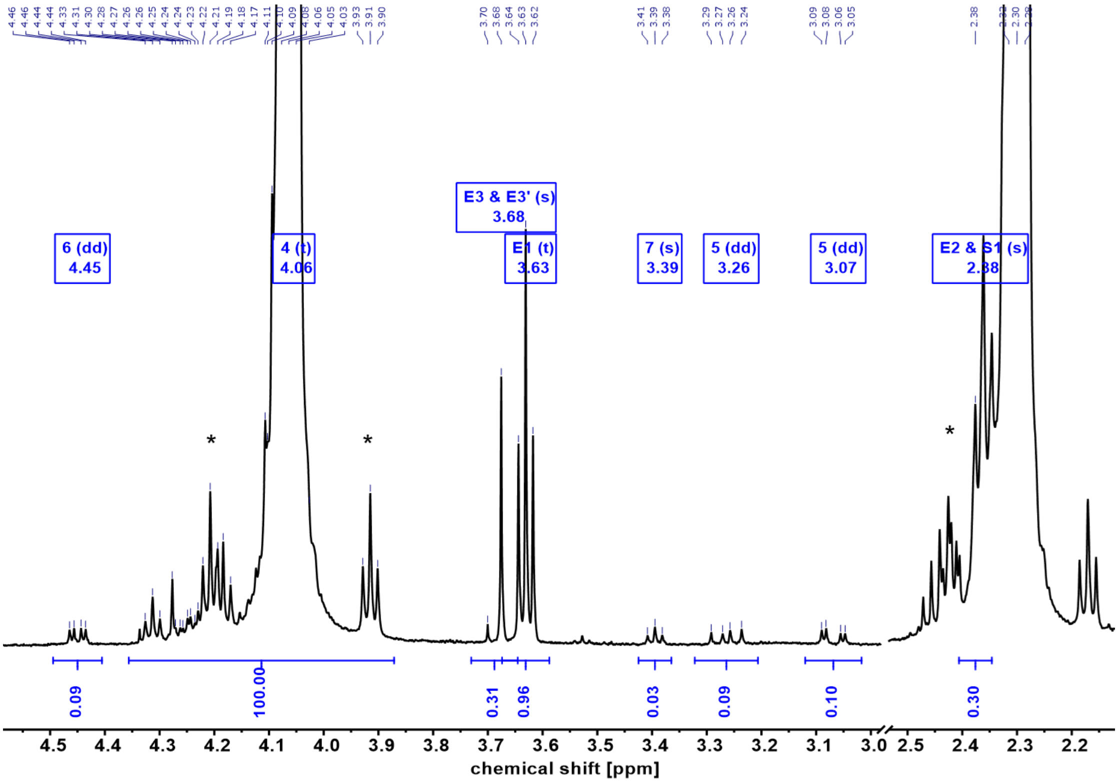

Figure S47 Detail of the ^{1}H NMR spectrum (500 MHz, 323 K, $C_2D_2Cl_4$) of **PE18.18-SO_3Ca** with integrals of end groups as well as protons located close to the sulfonate group (* = ^{13}C-coupled satellites). Assignment of signals according to **Figure S46**.

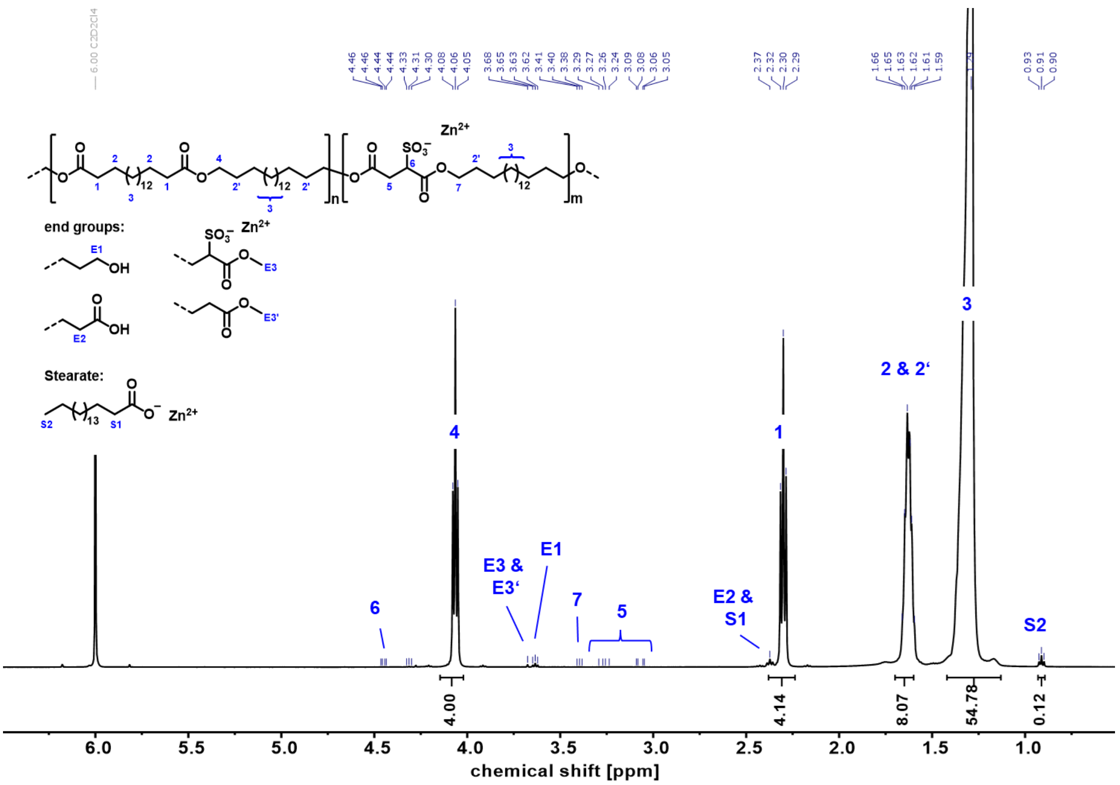

Figure S48 ^{1}H NMR spectrum (500 MHz, 323 K, $C_2D_2Cl_4$) of **PE18.18-SO$_3$Zn**. Note that protons 5 located next to the sulfonate group are diastereotopic and that the sulfonate group can be oriented either as shown, or the repeat unit can be oppositely arranged.

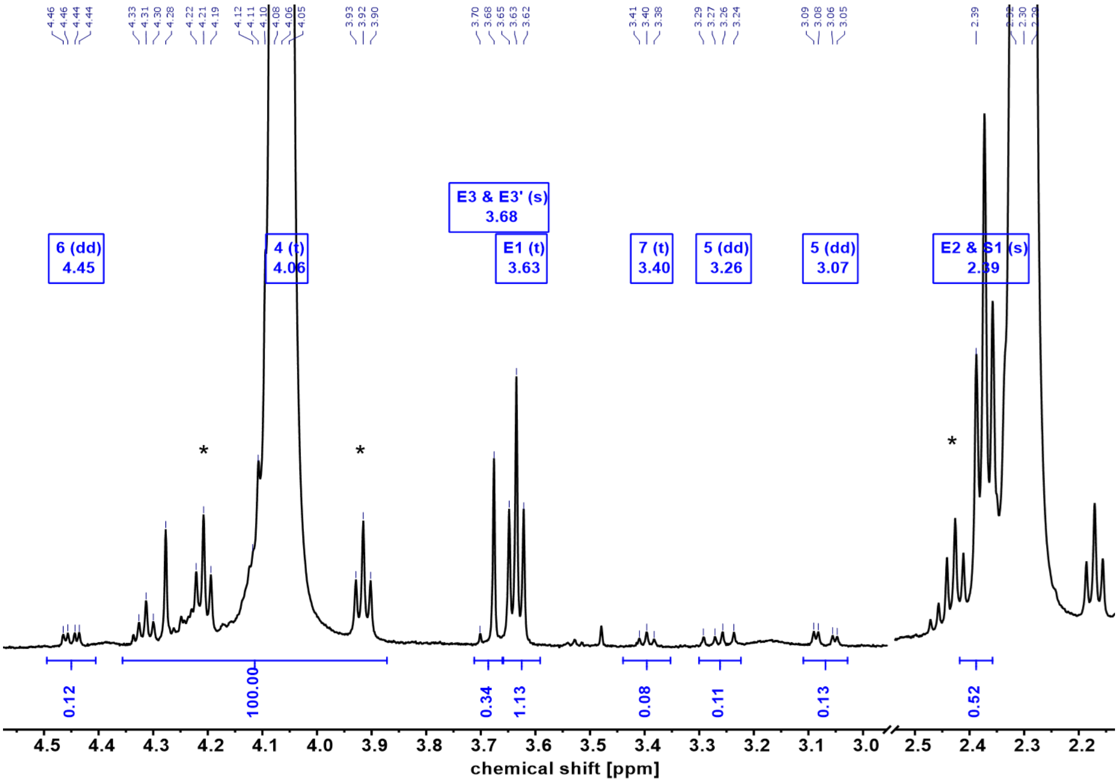

Figure S49 Detail of the ^{1}H NMR spectrum (500 MHz, 323 K, $C_2D_2Cl_4$) of **PE18.18-SO$_3$Zn** with integrals of end groups as well as protons located close to the sulfonate group (* = ^{13}C-coupled satellites). Assignment of signals according to **Figure S48**.

3.11. ¹H NMR Spectra of Composite Polyesters PE12.12-M$_{stearate}$

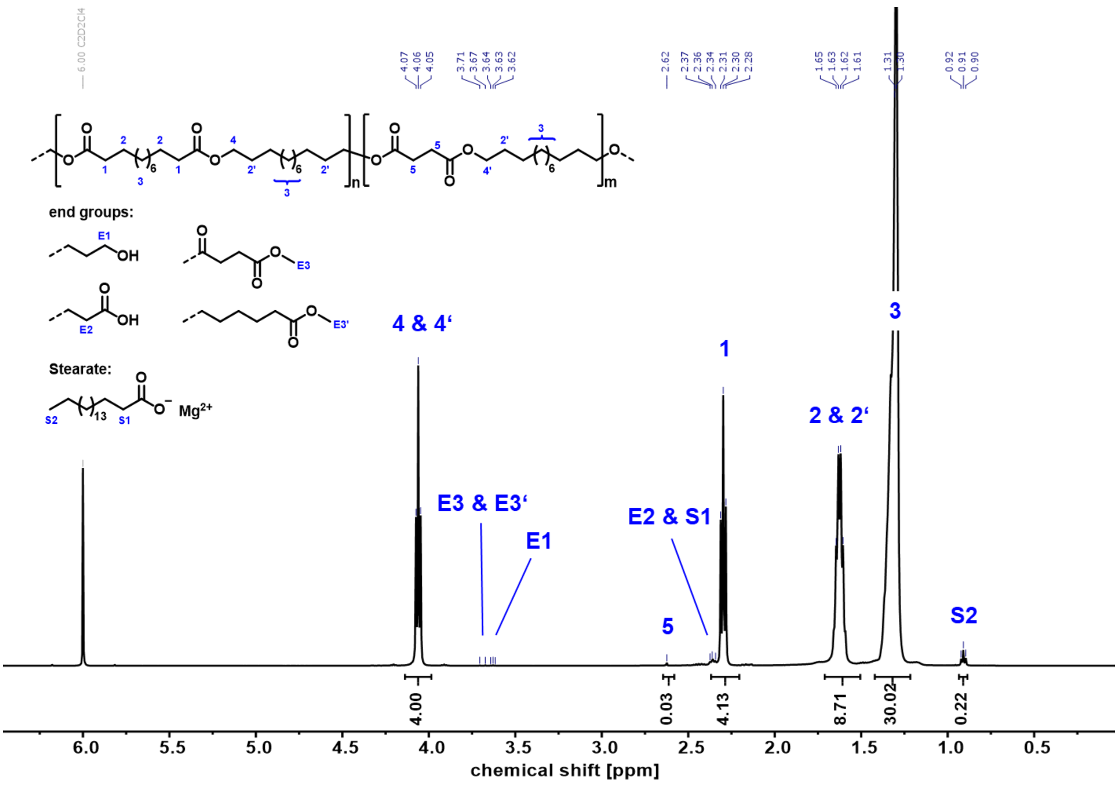

Figure S50 ¹H NMR spectrum (500 MHz, 323 K, $C_2D_2Cl_4$) of **PE12.12-Mg$_{stearate}$**.

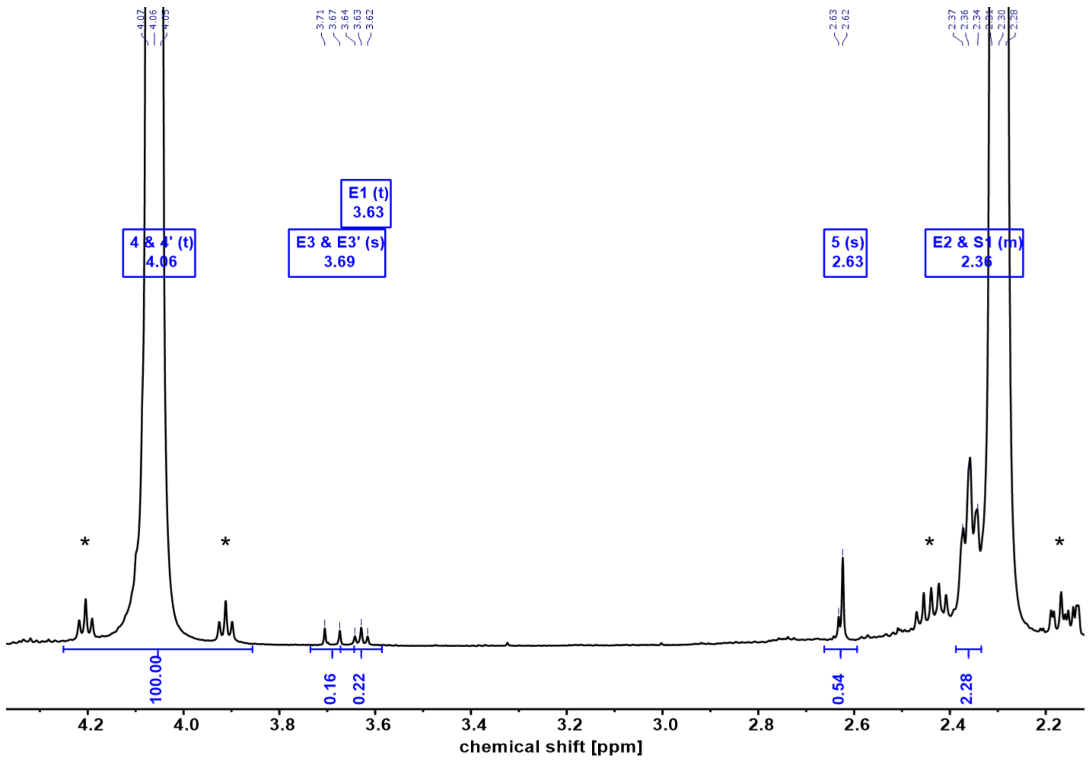

Figure S51 Detail of the ¹H NMR spectrum (500 MHz, 323 K, $C_2D_2Cl_4$) of **PE12.12-Mg$_{stearate}$** with integrals of end groups, the succinate unit and excess stearate (* = ^{13}C-coupled satellites). Assignment of signals according to **Figure S50**.

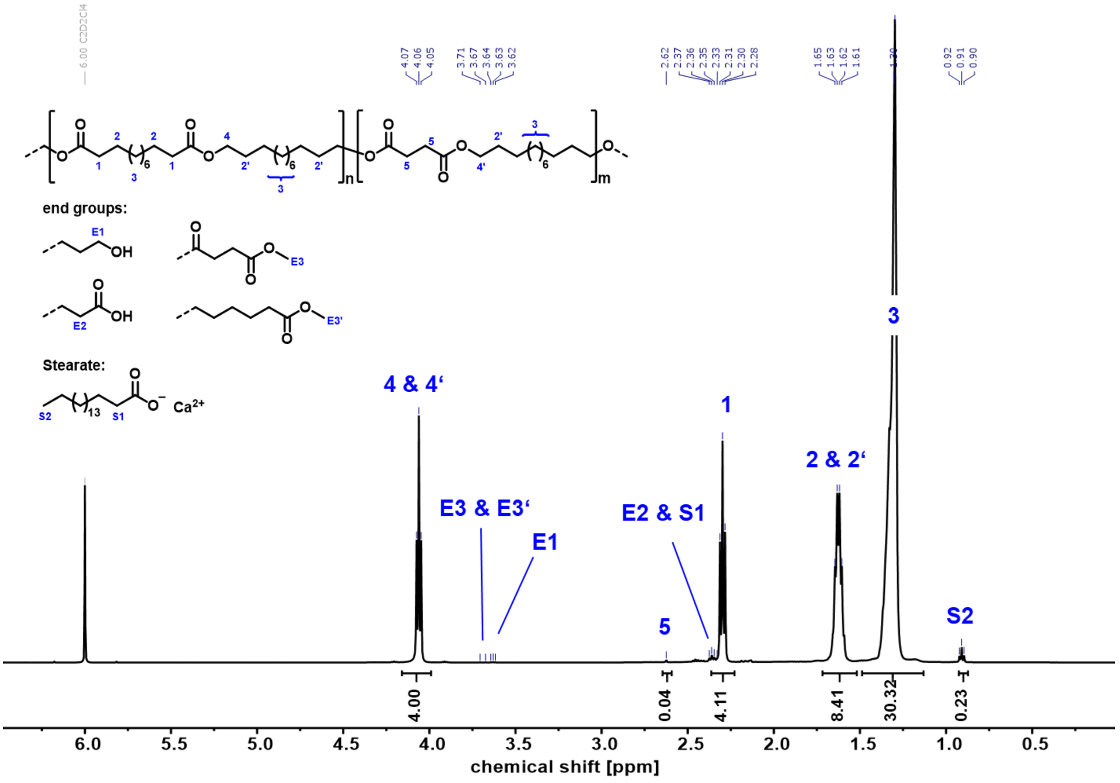

Figure S52 ^{1}H NMR spectrum (500 MHz, 323 K, $C_2D_2Cl_4$) of **PE12.12-Ca$_{stearate}$**.

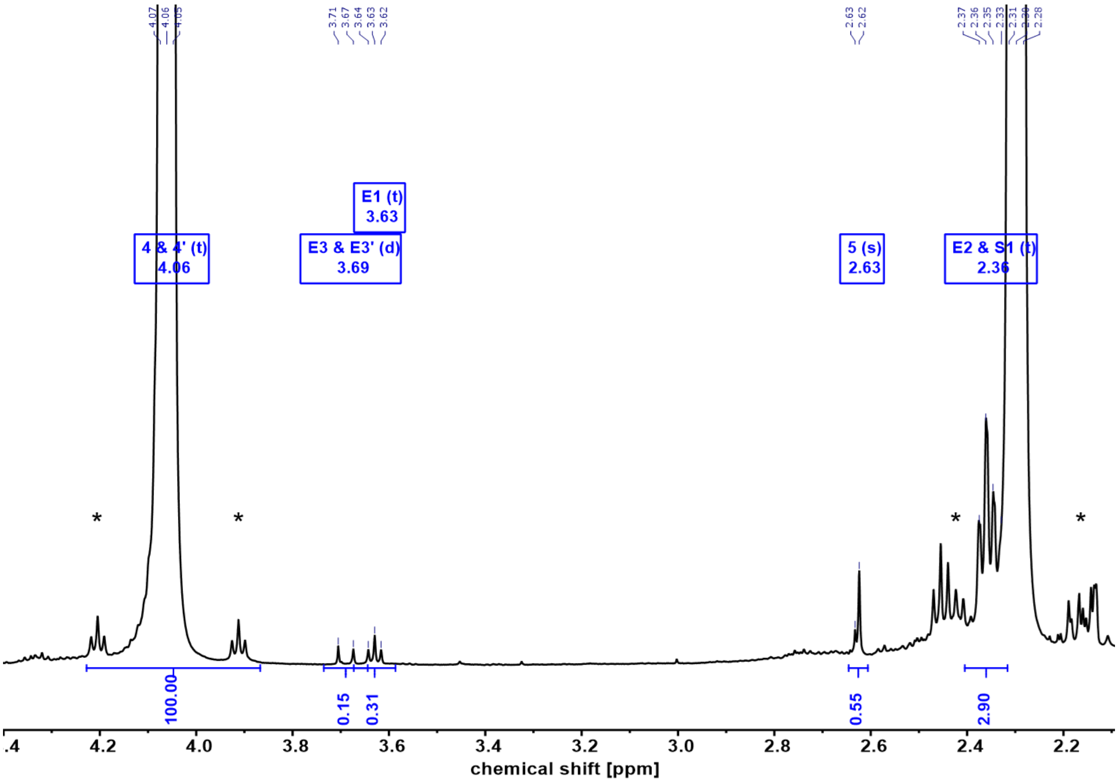

Figure S53 Detail of the ^{1}H NMR spectrum (500 MHz, 323 K, $C_2D_2Cl_4$) of **PE12.12-Ca$_{stearate}$** with integrals of end groups, the succinate unit and excess stearate (* = ^{13}C-coupled satellites). Assignment of signals according to **Figure S52**.

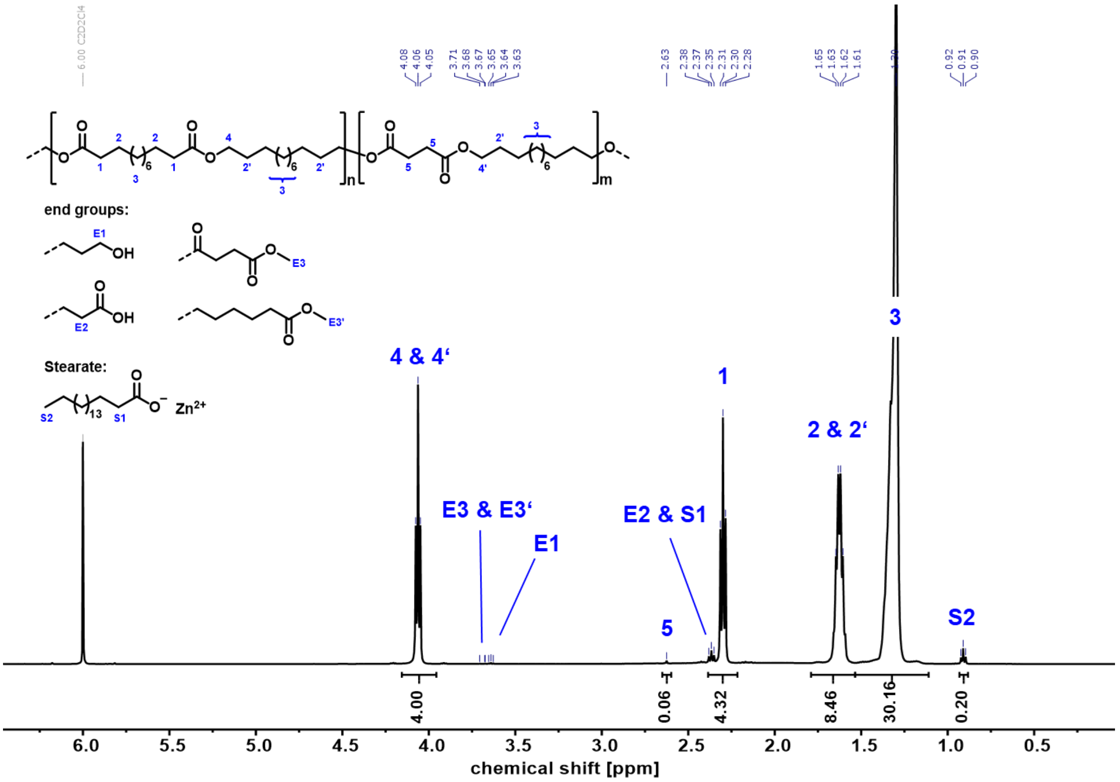

Figure S54 ^{1}H NMR spectrum (500 MHz, 323 K, $C_2D_2Cl_4$) of **PE12.12-Zn$_{stearate}$**.

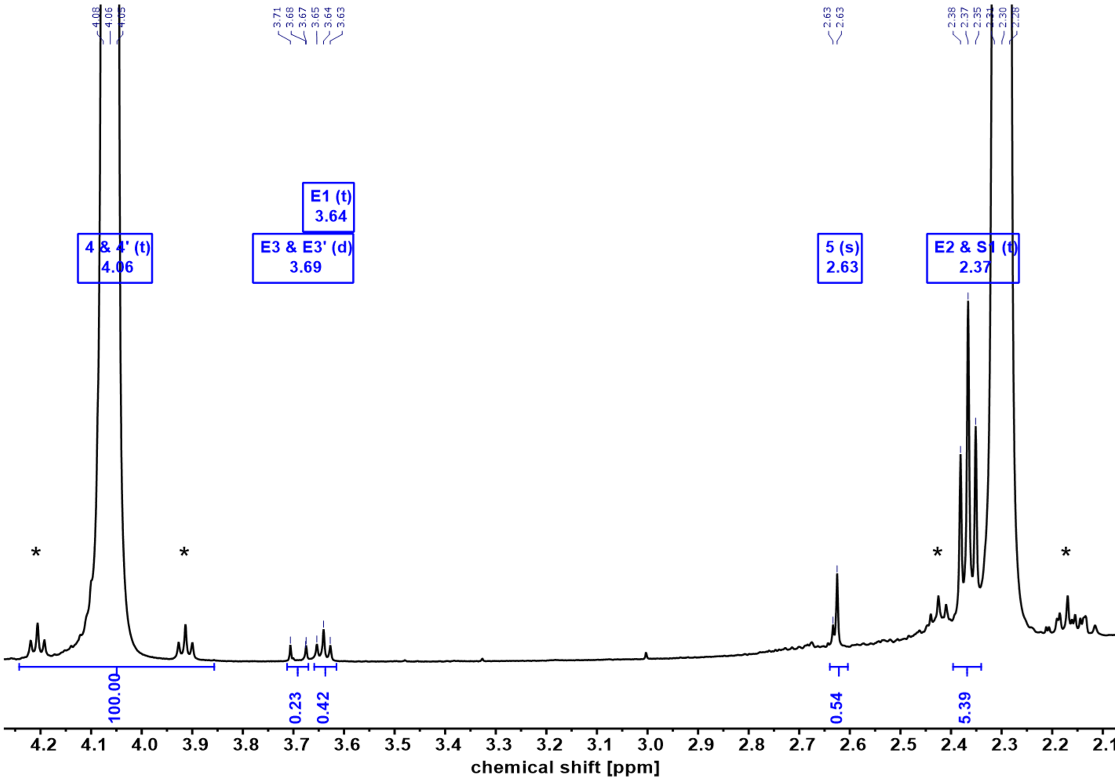

Figure S55 Detail of the ^{1}H NMR spectrum (500 MHz, 323 K, $C_2D_2Cl_4$) of **PE12.12-Zn$_{stearate}$** with integrals of end groups, the succinate unit and excess stearate (* = ^{13}C-coupled satellites). Assignment of signals according to **Figure S54**.

3.12. ^{1}H NMR Spectrum of Recycling to Monomer

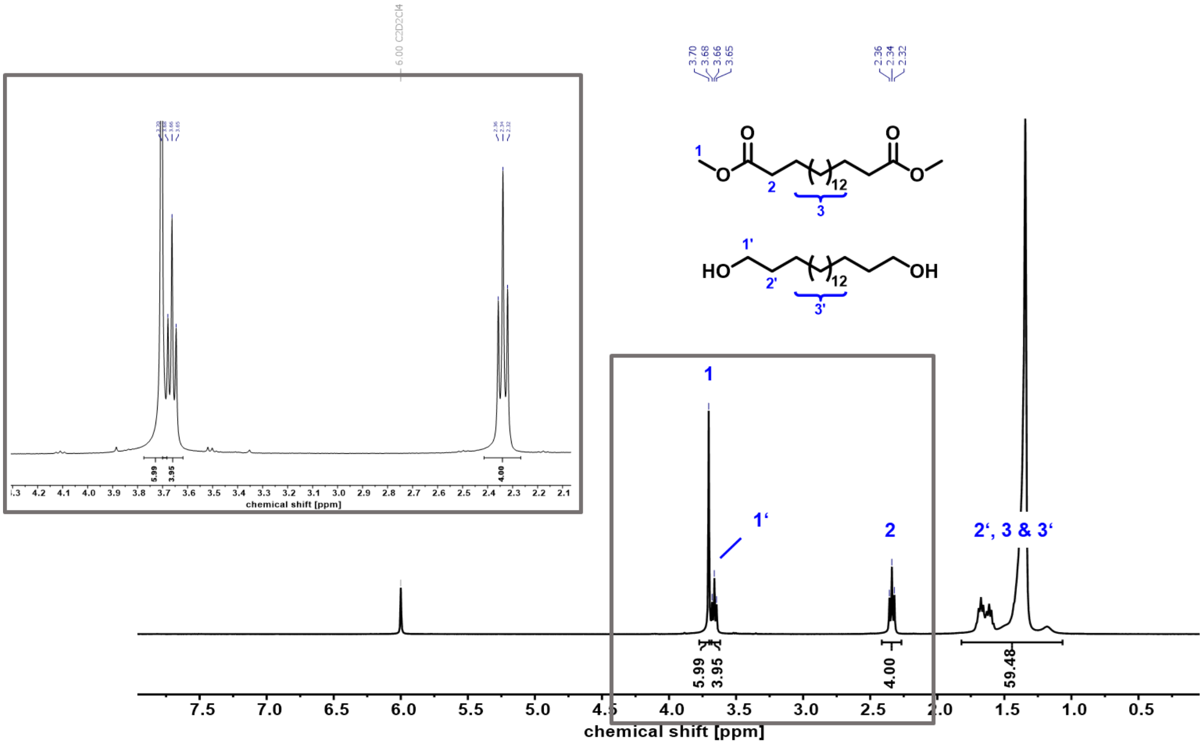

Figure S56 ^{1}H NMR spectrum (400 MHz, 383 K, $C_2D_2Cl_4$) of monomer regenerated from PE18.18-SO_3H-1.0. The product mixture contains C_{18}-diol and C_{18}-diester in a ratio of 1:0.99 as determined from the ratio of signal 2 (triplet, 2.34 ppm) and 1' (triplet at 3.66 ppm). The inset shows the grey marked region in closer detail and highlights the purity of the monomer mixture.

4. References

1. Rank, C.; Yan, L.; Mecking, S.; Winey, K. I., Periodic Polyethylene Sulfonates from Polyesterification: Bulk and Nanoparticle Morphologies and Ionic Conductivities. *Macromolecules* **2019,** *52* (21), 8466-8475.
2. Ortmann, P.; Heckler, I.; Mecking, S., Physical properties and hydrolytic degradability of polyethylene-like polyacetals and polycarbonates. *Green Chemistry* **2014,** *16* (4), 1816-1827.
3. Wu, S.; Yang, H.; Huang, S.; Chen, Q., Relationship between Reaction Kinetics and Chain Dynamics of Vitrimers Based on Dioxaborolane Metathesis. *Macromolecules* **2020,** *53* (4), 1180-1190.
4. Odenwald, L.; Wimmer, F. P.; Mast, N. K.; Schußmann, M. G.; Wilhelm, M.; Mecking, S., Molecularly Defined Polyolefin Vitrimers from Catalytic Insertion Polymerization. *Journal of the American Chemical Society* **2022,** *144* (29), 13226-13233.
